# The Impact of Affective Information on Working Memory: A Pair of Meta-Analytic Reviews of Behavioral and Neuroimaging Evidence

**DOI:** 10.1037/bul0000193

**Published:** 2019-04-25

**Authors:** Susanne Schweizer, Ajay B. Satpute, Shir Atzil, Andy P. Field, Caitlin Hitchcock, Melissa Black, Lisa Feldman Barrett, Tim Dalgleish

**Affiliations:** 1 Institute of Cognitive Neuroscience, University College London; 2 Department of Psychology, Northeastern University; 3 Department of Psychology, Hebrew University of Jerusalem; 4 School of Psychology, University of Sussex; 5 Medical Research Council, Cognition and Brain Sciences Unit, University of Cambridge; 6 Medical Research Council, Cognition and Brain Sciences Unit, University of Cambridge and Cambridgeshire and Peterborough NHS Foundation Trust, Cambridge, United Kingdom; 7 Athinoula A. Martinos Center for Biomedical Imaging, Massachusetts General Hospital, Boston, Massachusetts, and Department of Psychology, Northeastern University; 8 Medical Research Council, Cognition and Brain Sciences Unit, University of Cambridge and Cambridgeshire and Peterborough NHS Foundation Trust, Cambridge, United Kingdom

**Keywords:** working memory, emotion, mental health, frontoparietal control network, salience network

## Abstract

Everyday life is defined by goal states that are continuously reprioritized based on available, often affective information. To pursue these goals, individuals need to process and maintain goal-relevant information, while ignoring potentially salient information that distracts resources from these goals. Empirically, this ability has typically been operationalized as working memory (WM) capacity. A growing body of research is investigating the impact of information’s affective salience on WM capacity. In the present review we address this question by exploring the potential differential impact of affective compared with neutral information on WM, and the underlying neural substrates. One-hundred and 65 studies (*N* = 7,433) were included in the meta-analysis. Results showed negligible to small (*d̂* = −.07–.20) effects of affective information on behavioral measures of WM in healthy individuals (*n* = 4,936) that varied as a function of valence and task-relevance. Heterogeneity analyses were significant, demonstrating the need to identify further study-specific factors and individual differences that moderate affective WM. At the neural level (33 studies; *n* = 683), processing affective versus neutral material during WM tasks was associated with more frequent recruitment of the vlPFC, the amygdala, and the temporo-occipital cortex. In contrast to healthy individuals, across behavioral studies those suffering from mental health problems (*n* = 2,041) showed impaired WM accuracy (*d̂* = −0.21) in the presence of affective material. These findings highlight the importance of integrating behavioral and neural levels of analysis. Finally, these findings suggest that affective WM capacity may be a transdiagnostic mechanism associated with poor mental health.

## Public Significance Statement

The behavioral and neuroimaging meta-analyses showed that in psychologically healthy individuals there was limited support for behavioral working memory (WM) performance to be affected by affective information, whereas at the neural level WM in the presence of affective relative to neutral information was associated with differential recruitment of the salience network and the fronto-parietal control network. These findings highlight the importance of combining behavioral and neuroimaging research syntheses. Second, in individuals with mental health problems WM was significantly impaired by affective material. This suggests that WM performance on tasks including affective compared with neutral information may be a sensitive and transdiagnostic cognitive marker of mental health status.

Working memory^1^ (WM) constitutes a capacity-limited resource that temporally maintains and stores information (Baddeley, 2003) in the service of higher cognitive functions (fluid intelligence, for instance; Kane, Hambrick, & Conway, 2005). The vicissitudes of daily life frequently require such cognitive functions to operate in affectively laden contexts where much of the goal-relevant and goal-irrelevant information being processed has affective characteristics. Despite this, the impact of affective information on WM and the mechanisms through which that impact is realized remain poorly understood (Baddeley, 2003, 2013; Pessoa, 2009). Indeed, a consideration of WM in relation to affective phenomena has only recently attracted concerted discussion (Baddeley, 2013; Barrett, Tugade, & Engle, 2004; Okon-Singer, Hendler, Pessoa, & Shackman, 2015). Here, we review the literature and synthesize the research data comparing the impact of affective versus neutral information on WM, and the underlying neural substrates.

## WM in the Laboratory Versus WM in the Outside World

Traditionally, WM has been experimentally assessed using paradigms that require individuals to update affectively neutral information such as numbers, letters or shapes in their memory store while simultaneously trying to minimize interference from other affectively neutral irrelevant material (e.g., Conway et al., 2005; Owen, McMillan, Laird, & Bullmore, 2005). These “affect-neutral” tasks are traditionally conducted in laboratory settings allowing extreme precision in the goal-demands placed on participants. However, in real-world contexts WM is deployed in the face of ever-changing goal-demands where the information that needs to be updated and maintained in WM to meet current goals can shift rapidly. Dynamic reprioritization of active goal-states typically occurs as salient and/or novel representations are selectively attended to (Corbetta & Shulman, 2002; Klink, Jentgens, & Lorteije, 2014). Salience can be perceptual (Corbetta & Shulman, 2002) or experience-driven (e.g., affect-neutral pictures and words attract attention when they are relevant to current task-goals; Vogt, De Houwer, & Crombez, 2011; Vogt, De Houwer, Crombez, & Van Damme, 2013).

Another source of salience concerns the affective properties of encountered information. Affective significance can be conferred by: learned associations (e.g., repeated exposure to an object’s rewarding properties; Gallagher & Holland, 1994; Gottfried, O’Doherty, & Dolan, 2003; Schoenbaum & Roesch, 2005), evolutionarily transmitted predispositions, for instance species-specific survival threats (i.e., biological preparedness or “inherent goal states”; LeDoux, 2012; Mobbs, Hagan, Dalgleish, Silston, & Prévost, 2015), as well as perceiver-based categorizations and appraisals (Barrett, 2006; Scherer, Dan, & Flykt, 2006). These perceiver-based conceptual pathways have the potential to overwrite inherent or learned associations about a stimulus’ affective impact (e.g., Ochsner, Bunge, Gross, & Gabrieli, 2002). Salience attribution to affective properties is likely to have developed phylogenetically in humans as a function of threat/reward detection mechanisms (Dolan, 2002; LeDoux & Brown, 2017) and more broadly as a heuristic for accelerating goal-directed behavior (Al-Shawaf, Conroy-Beam, Asao, & Buss, 2016; Barrett, 2013).

Imagine the case of a fire alarm going off during dinner preparations which involve the maintenance of necessary cooking steps in WM. The fire alarm will immediately lead to reprioritization of the goal of cooking as entirely insignificant, while the new goal of exiting the building with kin becomes the dominant priority in the goal hierarchy. This reprioritization occurs because of the alarm’s strong learned association with danger allied with the perceiver’s appraisals of how events are likely to unfold if no action is taken (Amo et al., 2014; Gilmartin, Balderston, & Helmstetter, 2014; Moscarello & Maren, 2018).

Outside the rarefied setting of the laboratory, information processed in WM, then, is evaluated in terms of its relative facilitation versus interference of the pursuit of current goal-states (Barrett, 2005; Clore & Huntsinger, 2007; Fox, 2008; Power & Dalgleish, 2015), and the affective significance of encountered information has the potential to initiate the overriding of currently active goals in order to prioritize other goal-states (Barrett, 2013; Krieglmeyer, Deutsch, De Houwer, & De Raedt, 2010) due to their salience to, for example, survival (LeDoux, 2012) or self-identity (Kendzierski, Ritter, Stump, & Anglin, 2015), or other domains central to the welfare of the organism.

## Theories About the Impact of Affective Properties on WM

Despite this almost ubiquitous requirement for WM in the real world to operate in affective contexts, we currently lack a compelling unified theory of the different ways in which the processing of affective information can impact on WM. Instead, most theoretical work has focused on the role of acute or trait affective states on WM processing (for reviews, see: depression, Baddeley, 2013; mood, Mitchell & Phillips, 2007; anxiety, Moran, 2016). Other theories have offered frameworks about the impact of affective information within other domains of cognition including attention (Mather & Sutherland, 2011; Vuilleumier, 2005; Vuilleumier & Huang, 2009; Wells & Matthews, 2015) and memory (Hamann, 2001; Phelps, 2004, 2006; Talmi, 2013) and some of these theories make specific predictions regarding WM (e.g., Mather & Sutherland, 2011). Common to these diverse models is the proposal that affective properties of encountered information modulate the strength of its resultant cognitive and neural representations. This can then facilitate or impair goal-directed behavior, depending on whether the affective information is relevant to the goal at-hand or to an alternative competing goal, respectively. A theoretical framework that enshrines this common component across models is Pessoa’s *dual competition framework* (DCF; Pessoa, 2009). Specifically, the DCF proposes that affective properties of encountered information can compete for processing resources within the cognitive system either at the level of perceptual processing or at the level of executive control. Thus, at any one time, cognitive resources devoted to the processing of affective properties become temporarily unavailable to all other goal-relevant properties, thereby interfering with goal-directed behavior that depends on these other properties.

## Investigating the Impact of Affective Properties on WM in the Laboratory and the Brain Scanner

How can we evaluate with some precision the impact of affective context on WM? Prototypically, the impact of affective properties on WM is tested by populating standard experimental tasks, administered in the laboratory, with affective stimuli. One way to do this is to solicit and use personally relevant affective information from participants. However, this tends to introduce sources of variance across participants regarding stimulus attributes that are unrelated to their affective properties (e.g., word length). Researchers therefore more commonly opt for “standardized” affective stimuli that pertain to prototypical affective goals presumed to be more or less relevant to all participants (e.g., survival motives). These can include words (Bradley & Lang, 1999), faces (Tottenham et al., 2009), and other affective images (Lang, Bradley, & Cuthbert, 2008). However, the potential downside of using such standardized stimuli is that their affective significance—their positive or negative value to a healthy research participant—will usually be relatively low (Pessoa, 2009). That is, while these generic stimuli are still likely to receive some preferential processing within the cognitive system, their modulating effect on current task performance is proposed to be limited—they are given what the DCF calls *soft prioritization* (Pessoa, 2009). At the behavioral level this relatively weak impact on prioritization is likely to be both difficult to detect and replicate, as well as being subject to strong influences from study-specific factors such as WM load. To translate this to our aforementioned real-world example of preparing dinner, imagine seeing news footage about a building on fire instead of hearing an alarm go off in your building. The footage may mildly interfere with the updating of the individual cooking steps in WM (e.g., forgetting to add salt), but is unlikely to have a fundamental effect on the priority of your goal to prepare dinner.

At the neural level, however, the effects of soft prioritization of standardized affective information should be easier to assess because the neural impact of the processing of affective information will be detectable even in situations where there has been no marked effect on overt behavior. Affective compared with neutral stimuli are proposed to have stronger perceptual representations in the brain’s visual cortices (Vuilleumier, 2005) and other sensory cortices for nonvisual stimuli (Satpute et al., 2015). This increased strength of representation is in part proposed to be a function of amygdalergic projections to cortical sensory areas (Amaral, Behniea, & Kelly, 2003; Sah, Faber, Lopez De Armentia, & Power, 2003) and has the potential to modulate executive competition by prioritizing attention toward affective compared with neutral stimuli. A second neural route through which executive competition can be impacted as a function of a stimulus’ affective significance is through the direct processing of affective information in the fronto-parietal control network (Okon-Singer et al., 2015; Pessoa, 2009). This would mean that executive resources are occupied by the processing of the affective information and thus no longer available for executive control- (here, WM-) demanding activities (Eysenck, Derakshan, Santos, & Calvo, 2007). Specifically, processing of affective information includes a wide range of potential processes including but not limited to valuation/appraisal of affective material with neural substrates distributed across the prefrontal cortex including a hub in the orbitofrontal cortex (Dixon, Thiruchselvam, Todd, & Christoff, 2017), and affect regulation involving multiple regions in the fronto-parietal control network including the lateral as well as the medial prefrontal and parietal cortices (Buhle et al., 2014; Etkin, Büchel, & Gross, 2015). Moreover, affective distractors and targets are likely to engage both overlapping and separate components of the frontoparietal control network (Dolcos, Katsumi, Denkova, & Dolcos, 2017). In sum, then, perceptual competition from affective (relative to neutral) material during the performance of a WM task should be associated with increased neural activation within the visual cortex (for affective visual stimuli) as well as within the brain’s “salience network” (Seeley et al., 2007; cf. ventral attention; Corbetta & Shulman, 2002), including the amygdala (Barrett & Satpute, 2013). Executive competition should also be reflected in augmented activation of the salience network and additionally with enhanced recruitment of the fronto-parietal control network (Pessoa, 2008, 2009).

This analysis suggests then that the behavioral and neural effects of affective stimuli on WM may be “dissociable.” It is hypothesized that there will small behavioral effects, because the stimuli prototypically used in the laboratory ultimately have low affective significance and only attract soft prioritization, allied to clear neural effects representing the analysis of the stimuli’s affective significant in preparation for any prioritization in the domain of behavior. A growing body of literature suggests that the impact of affective material on WM performance vary depending on the stimuli’s task-relevance (i.e., opposing effects of task-relevant material vs. task-irrelevant distractors) and in some cases the stimuli’s affective valence (Dolcos et al., 2017; Okon-Singer et al., 2015; Pessoa, 2009).

### Task-Relevance

Preferential allocation of perceptual and executive processing resources to task-relevant affective stimuli is proposed to improve behavioral performance on the task at hand.^2^ This affective enhancement effect is well-established in the long-term memory literature (for reviews of laboratory and neuroimaging studies, see Buchanan & Adolphs, 2002; Hamann, 2001; LaBar & Cabeza, 2006; Phelps, 2004) with individuals remembering affective information and events better compared with neutral information. Similarly, research on “emotional attention” shows reliable affective processing biases with individuals being faster to detect affective information in visual searches (Vuilleumier & Huang, 2009). Evidence from behavioral research on WM in healthy individuals appears more mixed with some studies showing an enhancement of WM for affective compared with neutral information (e.g., Xie et al., 2017), others showing no effect (e.g., Grissmann, Faller, Scharinger, Spüler, & Gerjets, 2017; M. Li et al., 2018; Nejati, Salehinejad, & Sabayee, 2018), WM impairment (e.g., Garrison & Schmeichel, 2018; Hur, Iordan, Dolcos, & Berenbaum, 2017; Tamm, Kreegipuu, Harro, & Cowan, 2017; Yoon, Kutz, LeMoult, & Joormann, 2017), or complex interactions with task-design features (e.g., trial type; Levens, Armstrong, Orejuela-Dávila, & Alverio, 2017; Quinlan, Yue, & Cohen, 2017). Meta-analytic synthesis of the relevant evidence is therefore required to elucidate the potential impact(s) of affective memoranda on WM.

Greater perceptual- and executive-level prioritization of task-irrelevant (henceforth, distractors) affective, relative to neutral, stimuli is hypothesized to impair behavioral WM performance (e.g., Derakshan & Eysenck, 2009; Okon-Singer et al., 2015; Pessoa, 2009). This is in line with evidence from tasks assessing executive control in processes other than WM (e.g., dichotic listening tasks, modified Stroop tasks or spatial attention tasks; Schupp, Flaisch, Stockburger, & Junghöfer, 2006; Yiend, 2010). The literature on the impact of affective distractors on WM performance again is mixed, showing no effect (Jenness et al., 2018) or impaired behavioral (Ladouceur, Schlund, & Segreti, 2018; Stout, Shackman, Pedersen, Miskovich, & Larson, 2017; Tollenaar, Ruissen, Elzinga, & de Bruijn, 2017; Wingert, Blais, Ball, & Brewer, 2018) performance.

At the neural level, the inferior PFC is considered critical to selecting task-relevant targets and inhibiting responses and attention to task-irrelevant distractors (Aron, Robbins, & Poldrack, 2004; Miller & Cohen, 2001). However, recent reviews of the literature on the neural substrates of affect-cognition interactions suggest that the inhibition of attention and responses to, as well as the regulation of, affective distractors may recruit a wider network in the ventral stream of the fronto-parietal control network (Iordan & Dolcos, 2017; Okon-Singer et al., 2015), this includes the inferior PFC but is not limited to it. WM tasks performed in the presence of affective distractors have similarly shown greater recruitment of the ventral PFC (e.g., Dolcos & McCarthy, 2006), though some studies have also shown the involvement of more dorsal and medial parts of the frontoparietal control network (García-Pacios, Garcés, Del Río, & Maestú, 2015, 2017). The current neuroimaging meta-analysis allows us to investigate the relative contributions of these different brain regions to the interference from affective compared with neutral distractors.

### Valence

The vast majority of the experimental literature on WM in affective contexts, to-date, focuses on the impact of negatively valenced information (usually threat-related). However, comparable theoretical arguments to those articulated above can be made for the effects of positive stimuli, neutral stimuli with high-arousal associations (Mourão-Miranda et al., 2003), and novel stimuli. Except in some circumstances (e.g., erotic stimuli, see below) the positive stimuli prototypically used in laboratory studies are considered very low in affective significance (Pereira et al., 2006; Pessoa, 2009) and are thus unlikely to elicit robust behavioral effects let alone reprioritize current goals. Studies on the temporal course of peripheral physiological responses to affective information in laboratory contexts support this notion with responses to negative material being faster (N. K. Smith, Cacioppo, Larsen, & Chartrand, 2003)^3^ and more protracted than for positive stimuli (Brosschot & Thayer, 2003; Taylor, 1991). Similarly, while both pleasant and unpleasant stimuli engage overlapping parts of the brain’s salience network, neural responses are nevertheless less reliable for pleasant than unpleasant stimuli in the amygdala and insula (Lindquist, Satpute, Wager, Weber, & Barrett, 2016). Evidence from WM appears to show a comparable pattern, with positive stimuli having a lower impact on performance compared with negative stimuli, though the effect of valence may be stronger for WM reaction time (RT) data compared with accuracy data (e.g., Colligan & Koven, 2015). Furthermore, there may be developmental differences with performance being more affected by rewarding stimuli in adolescence (Cromheeke & Mueller, 2016). The reviewed work then suggests that the impact of affective material as evaluated in laboratory WM tasks will be greater for negative compared with positive material.

## The Impact of Affective Information on WM Beyond Young Psychologically Healthy Adults

Theoretically, stimuli high in affective significance are proposed to have pronounced effects on behavioral performance through the recruitment of common executive control resources in the service of processing these affectively laden stimuli—consider our real-world fire alarm example. Pessoa (2009) terms this *hard prioritization*. Such hard prioritization is difficult to investigate in the laboratory with psychologically healthy individuals as the standardized stimuli used in such studies, as discussed above, are low in affective significance. Indeed, to our knowledge, no study has systematically modulated stimuli’s affective significance to investigate the nature of the relationship between affective significance and WM performance.

However, one way that prioritization can be investigated experimentally is to work with populations—such as samples characterized by mental health difficulties—for whom standardized stimuli are evaluated as relatively high in affective significance. Affective information, it is proposed, gains harder prioritization in individuals suffering from mental health problems because it is critical to the individual’s perpetually activated affect-related concerns. That is, many mental health difficulties (including mood and anxiety disorders, schizophrenia, and attention deficit and hyperactivity disorder; Aleman & Kahn, 2005; Bar-Haim, Lamy, Pergamin, Bakermans-Kranenburg, & van IJzendoorn, 2007; Cubillo, Halari, Smith, Taylor, & Rubia, 2012; Gotlib & Joormann, 2010; Mathews & MacLeod, 2005) are associated with: preferential processing of affective, particularly negative, information; slowed disengagement from affective information (e.g., depression; Koster, De Lissnyder, Derakshan, & De Raedt, 2011); and maladaptive regulation of affective material (Aldao, Nolen-Hoeksema, & Schweizer, 2010). The impact of affective material on WM then is likely to be increased in individuals suffering from mental health problems compared with healthy controls. This is also likely to be the case for affectively positive stimuli. For example, individuals with eating-related mental health (Wagner et al., 2015) and physiological weight-related problems (Boutelle et al., 2014) show increased activation of the amygdala in response to standardized food-related stimuli compared with healthy individuals.

Related to this, another possible moderator of perceived affective significance is age. There is a wealth of evidence that for older adults positive stimuli may carry greater affective significance due to the age-related positivity effect—the finding that in older-age individuals preferentially process positive information across a range of cognitive domains and stimulus types (Carstensen, 2006; Mather & Carstensen, 2005). Comparing WM performance for affective information across age then may reveal dissociable effects for positive and negative stimuli.

To summarize, broadly speaking the effects of affective material on WM processing are hypothesized to vary as a function of the material’s affective significance, valence, and task-relevance. Furthermore, the behavioral and neural levels of analysis are predicted to show dissociable effects for prototypical studies involving standardized stimuli with low-affective significance administered to unselected or psychologically healthy populations. In the sections that follow, we outline specific hypotheses within each of these sets of circumstances before reviewing the relevant data.

## The Present Reviews

The primary aim of the current reviews was to evaluate both the behavioral impact of affective information on WM performance and the neural substrates of those putative effects, through a pair of meta-analyses of the extant literatures. To this end we reviewed behavioral and functional MRI (fMRI) studies published up until February 28, 2017, that investigated the effect of affective material on WM functioning.

**Figure 1 fig1:**
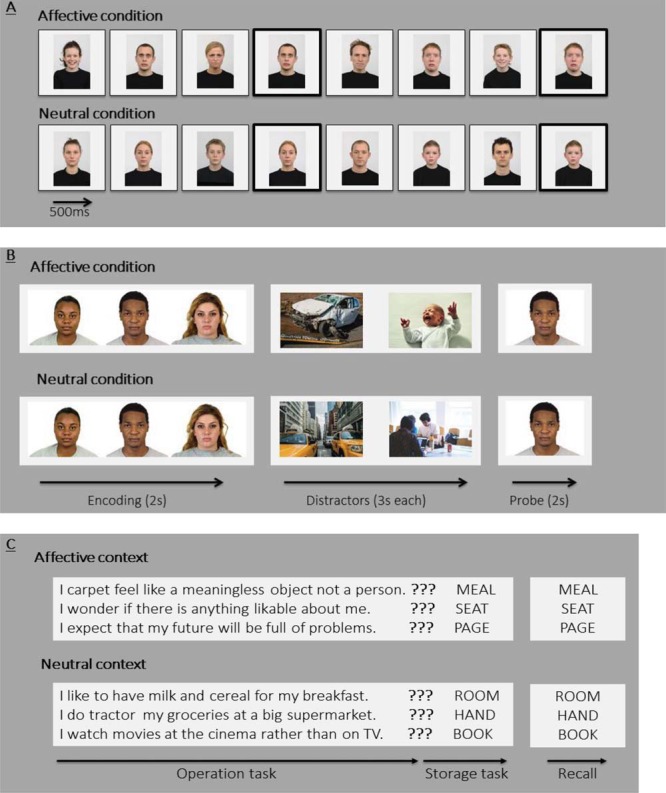
Schematics of three prototypical WM tasks, presented in affective and neutral contexts. The figure depicts three prototypical WM tasks capturing the range of paradigms included in the present meta-analyses. 1A shows an *n*-back task (where in this case *n* = 2) that requires participants to continuously update the content of their active WM representations. In the figure, trials with a bold black border indicate target trials. In the affective context the images that have to be matched across trials are negative in valence and in the neutral context the target stimuli are neutral. 1B depicts an example of a delayed-match-to-sample task. In this task, participants are required to match the emotional expression of a probe face with the expression in one of three presented memoranda. During the retention interval participants see either two negative distractor images (affective context) or two valence-neutral images (neutral context). 1C provides an illustration of a complex span task, which comprises an operation component and a storage component. The example depicts an affective reading span task where participants make judgments about the semantic accuracy of self-statements. In the affective context the first sentence requires a “no” response as it is semantically meaningless, while the other sentences are semantically correct. In the neutral context the second sentence is incorrect and the others are semantically meaningful. For the storage component participants have to recall the words in upper case that are presented at the end of each sentence. The recall happens at the end of each block, with block lengths typically varying between three and seven trials.

Guided by the definition of WM as comprising one or more storage components alongside an executive control component, the present meta-analytic work included studies employing three types of tasks as measures of WM (see Figure 1 for task schematics): (a) tasks that require continuous updating of WM content through the sequential presentation of memoranda—these include simple span tasks and *n*-back tasks (Figure 1A; Cohen et al., 1997; Owen et al., 2005); (b) delayed-match-to-sample tasks (Figure 1B) that require the recall of memoranda following a delay interval during which participants are either presented with distractors or some other form of secondary task-demand^4^ (Courtney, Petit, Maisog, Ungerleider, & Haxby, 1998; Jiang, Haxby, Martin, Ungerleider, & Parasuraman, 2000; Sawaguchi & Goldman-Rakic, 1991); and (c) complex span tasks (Figure 1C) which comprise an operation task (e.g., solving a mathematical problem) and a storage task (e.g., remembering words; Conway et al., 2005). For a given study to be included in our analyses these tasks needed to present affective stimuli as either task-relevant memoranda (targets) or task-irrelevant distractors.[Fig-anchor fig1]

## Behavioral Meta-Analysis

In line with the previous theoretical discussion, the behavioral meta-analysis examined the following hypotheses:

*Hypothesis A:* In the context of a proposed dissociation between behavioral and neural levels of analysis in psychologically healthy individuals (or unselected), for the behavioral meta-analysis we predicted at most small effects of affective, relative to neutral, material on WM performance due to affective stimuli’ *low affective significance* and the predicted *moderating and interacting effects of valence (A1) and task relevance (A2).* Specifically, we hypothesized that:

*Hypothesis A1:* Positive stimuli have a smaller effect on WM performance compared with negative stimuli, and;

*Hypothesis A2:* Affective distractors and targets have opposing effects on WM performance, with affective distractors impairing WM performance relative to neutral distractors and affective targets enhancing WM performance relative to neutral targets.

*Hypothesis B1:* Affective stimuli have a greater impact on behavioral WM performance in individuals with *mental health problems* for whom it is proposed they have greater affective significance compared with healthy individuals.

*Hypothesis B2:* The impact of positive, but not negative, stimuli, relative to neutral stimuli, on WM performance increases as a function of age in line with the *age*-related positivity bias.

## Methods for the Behavioral Meta-Analysis

### Identification of Studies for Inclusion^5^

The literature search was conducted in accordance with the Preferred Reporting Items for Systematic Reviews and Meta-Analyses (PRISMA; Moher, Liberati, Tetzlaff, & Altman, 2009) guidelines (for the PRISMA Checklist, see online supplementary materials). There was no review protocol. All searches were executed in the databases PubMed and PsycINFO with the following search parameter delimitations—publication language: English, human participants, publication date: 01.01.1900 (default in PubMed)–28.02.2017 (for the electronic search strategy please see the online supplementary materials). The search term combinations entered were: Combination I^6^ = “emotion* OR affective AND executive* function*; Combination II = “emoti* OR affective AND cogniti* function*”; Combination III = “emoti* OR affective AND working memory”; Combination IV = “emoti* OR affective AND n-back”; and Combination V = “emoti* OR affective AND delayed-match-to-sample.” In addition to the articles yielded by the database search we also checked the reference lists of those articles.

### Screening

After removing all duplicates, review articles, and theoretical papers, articles generated in the identification stage were screened. Inclusion criteria in the screening phase were: Article titles needed to refer to two separate components: (a) the word “emotional” (or synonyms thereof) or a “mental health disorder,” as well as (b) the word “cognitive” or terms referring to “executive functioning.” Abstracts needed to mention the use of one or more memory tasks or refer to executive functioning tasks. This led to a set of full-text articles, which were assessed in the final step.

### Eligibility

Eligibility was assessed by checking the full-text articles for the following components: (a) They needed to report at least one empirical study in humans. (b) The studies also had to report accuracy performance and/or RTs on a measure of WM, which required the recall of affective and neutral task-relevant memoranda in WM, or contained affective and neutral task-irrelevant distractors which had to be ignored. If these data were not reported in the paper authors were contacted with a request for these data (denoted with data request [DR] in Table 1; studies that met all inclusion criteria but for which no data was made available are reported in the relevant online supplementary materials section). Studies that used mood induction or naturally occurring mood states (e.g., mania) as an emotion manipulation were excluded as this was beyond the scope of the present reviews.[Table-anchor tbl1]

To ensure that the search was performed in accordance to the search strategy outlined above 30% of all hits at the screening stage were checked by CH and MB in addition to the first author who completed the search for all entries. Interrater agreement was 89%. All conflicts for this stage were resolved in discussion between the first author and the two additional raters. Finally, all full text studies included in the final stage were checked by the twos additional raters. For this stage there was 100% independent interrater agreement.

### Analytic Approach

#### Behavioral analyses

##### Publication bias

The presence of publication biases (Rothstein, Sutton, & Borenstein, 2005) was tested in two steps. An approximation for multilevel analyses of the standard regression test (Egger, Davey Smith, Schneider, & Minder, 1997) examined whether the standard errors were significant predictors of the observed effect sizes. Considering that publication bias can vary as a function of study characteristics (Coburn & Vevea, 2015) and that we investigated several such characteristics as moderators of interest the regression analysis was supplemented with a regression test for multilevel implementation, where study variance is included as a moderator before running the rank correlation test (thereby approximating the Egger test for multilevel data).

##### Effect sizes

Effect sizes (Cohen’s *d̂*) were calculated using the *escalc* function in the *metafor* (Version 1.9–5; Viechtbauer, 2010) software package in R (Version 2.15.0; R Core Team, 2013) by dividing the mean difference of WM performance/response time in an affective versus neutral context by the unbiased estimates of the sampling variance. Unbiased estimates of the sampling variance are computed by applying a correction to the pooled standard deviation to correct for a slight positive bias within the standard error function (for a detailed discussion of the positive bias see: Hedges, 1982, p. 492; 1989).

##### Hypothesis testing

To test our hypotheses investigating the effects of affective context on WM performance (accuracy and response time) we conducted a random effects model analysis on the effect sizes of studies that directly compared WM in affective versus neutral contexts. This analysis was based on the premise that differences in methods and samples across the studies included in the meta-analysis would introduce variance (heterogeneity) among the true effects, which could be incorporated into the study weights (Hedges & Vevea, 1998).

The predicted moderating effects of task-relevance (target vs. distractor) and valence (positive vs. negative) were tested in the sample of healthy participants. Affective significance as a function of study population (healthy individuals vs. individuals suffering from psychopathology^7^) was investigated in the total sample. The association of WM and age was investigated with correlation analyses. All hypothesized moderator effects were investigated in a series of planned moderation analyses using multilevel models in which effect size (Level 1) is nested within the study (Level 2) estimated using the rma.mv() function in the *metafor* package (Version 1.9–5; Viechtbauer, 2010). This approach enabled the models to include multiple effect sizes from the same study. We fitted a random intercepts model, allowing effects sizes to be free to vary across studies. We chose to apply multivariate random- and mixed-effects models, because fixed-effects models have been shown to be too liberal, overestimating true effect sizes (Field, 2003; Hunter & Schmidt, 2000), whereas random-effects models are thought to provide better estimates of the true effect investigated (Schmidt, Oh, & Hayes, 2009). It should be noted, however, that random-effects models with relatively small sample sizes provide only approximations of the true effect (Schmidt et al., 2009).

All analyses were performed twice, once with WM accuracy as the outcome measure and once with WM RT.

## Results of the Behavioral Meta-Analysis

**Figure 2 fig2:**
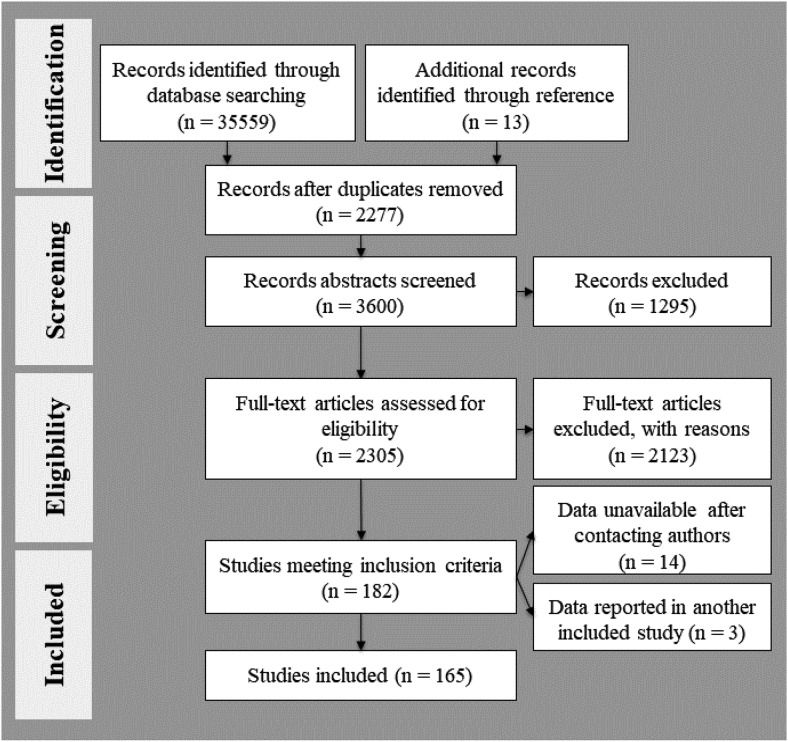
PRISMA flow-diagram for the behavioral meta-analysis. Reasons for exclusion are detailed in the online supplementary materials. For those meeting inclusion, data were unavailable due to departmental, personnel move, or data storage issues (de Almeida et al., 2012; DeYoung, Shamosh, Green, Braver, & Gray, 2009; Lindström & Bohlin, 2012; Maat et al., 2014; Mirabolfathi, Moradi, & Bakhtiari, 2016) and we did not receive replies from the following authors (Chen, Feng, Wang, Su, & Zhang, 2016; Diwadkar et al., 2012; Fan, Hsu, & Cheng, 2013; Gotoh, 2008; Liu, Wang, Wang, & Jiang, 2016; Luo et al., 2014; Mackay et al., 2004; Pecchinenda & Heil, 2007; Shi, Gao, & Zhou, 2015). The following publications were included as part of other citations included in the analysis (García-Pacios, Garcés, Del Río, & Maestú, 2017; Krause-Utz, Elzinga, Oei, Paret et al., 2014; Luksys et al., 2014).

Figure 2 provides a schematic overview of the search results for the behavioral meta-analysis (PRISMA flow diagram; Moher et al., 2009). One-hundred and 65 data sets were included in the present meta-analyses. Table 1 provides a list of the included studies together with an overview of their task designs, participant samples, task-relevance and valence of the included affective stimuli, and whether the studies reported functional neuroimaging data.[Fig-anchor fig2]

### Publication Bias

**Figure 3 fig3:**
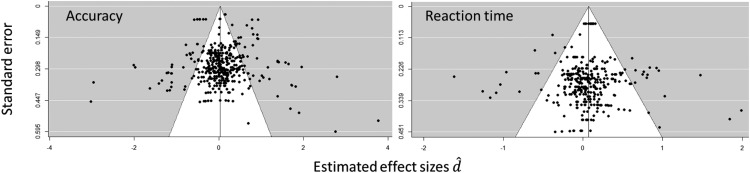
Funnel plots for studies reporting accuracy and RT for the behavioral meta-analysis. For WM accuracy the plot in the left box shows, from left to right on the *x*-axis, studies where WM performance is impaired by the presence of affective compared with neutral stimuli through to studies where WM is more accurate in the presence of affective relative to neutral stimuli. In the right-hand box the RT plot shows the distribution of effect sizes for studies showing faster response times for affective compared with neutral from left to the right of the middle line, from which point onward studies showed slowed RTs for affective compared with neutral stimuli.

The funnel plots (see Figure 3) for accuracy and RT show the distribution of the standardized mean difference (observed outcome) between accuracy and RT for affective compared with neutral WM across the standard error distribution. The regression test of the publication bias was nonsignificant for both accuracy (*z* = −1.33, *p* = .184) and RT (*z* = −1.64, *p* = .101). The regression test for multilevel implementation was also non-significant for both accuracy (Kendall’s τ = −0.02, *p* = .523) and reaction time (Kendall’s τ = −0.02, *p* = .518), which suggests that there was no significant publication bias in the set of studies included in the meta-analytic review.[Fig-anchor fig3]

### Overall Effect of Affective Context on WM in Psychologically Healthy Individuals (Hypothesis A)

Consistent with Hypothesis A, although the multivariate random effects analysis in healthy individuals showed that RTs for affective compared with neutral material in WM were significantly slowed, the effect size was of trivial magnitude *k* = 317, *d̂* = 0.07, 95% CI [0.03, .12], *SEM* = 0.02, *p* = .002. Furthermore, there was no significant effect of affective information on WM accuracy, *k* = 391, *d̂* = 0.03, 95% CI [−0.05, 0.12], *SEM* = 0.04, *p* = .438. In addition to the effect sizes, we report the omnibus *Q*-tests of heterogeneity, because the statistic is less disposed to Type I errors than other tests of heterogeneity (Viechtbauer, 2007). The estimated heterogeneities in the overall effect sizes accounted for by the differential effect of affective compared with neutral material on both WM accuracy, *Q*(390) = 2609.79, *p* ≤ .0001, σ^2^ = 0.21; and RT, *Q*(311) = 529.19, *p* ≤ .0001, σ^2^ = 0.02, were significant. That is, for both WM accuracy and RT a significant amount of variance is likely to be accounted for by variations in study-specific factors.

The moderating effects of valence (Hypothesis A1), task-relevance (Hypothesis A2), and mental health status (Hypothesis B1) and age (Hypothesis B2) are tested below. However, given the substantial amount of heterogeneity in the results we additionally explored the potentially moderating effects of emotion-type (fear, anger, sad, happy) and WM task load. Differential influences of emotion-type might partially account for the heterogeneity in the results because threat-related stimuli might be more arousing, and thus impact WM, more compared with sadness-related stimuli (Saxton, Myhre, Siyaguna, & Rokke, 2018; Vuilleumier, 2002). The rationale for WM load as an additional moderator is that it has been shown to influence attentional control (Lavie, Hirst, de Fockert, & Viding, 2004). The load theory of selective attention and cognitive control (Lavie et al., 2004) would suggest that the impact of affective relative to neutral material is greatest for lower levels of WM load. The results showed that WM RT, but not WM accuracy, was moderated by emotion type. For RT the moderating effect of emotion type reflected the valence effect (Hypothesis A1) reported below, with RTs being faster in the context of happiness-related versus neutral stimuli, whereas all negative emotions were associated with relatively slower RTs (see SM6 for a full set of statistics and results). For WM load there was no main effect of load on either WM accuracy or RT, *p*’s > .648. WM load did, however, interact with task-relevance, indicating that WM accuracy (not RT) for task-relevant affective, relative to neutral, targets improved across load, *r*(130) = .24, 95% CI [.07, .39], *p* = .006 (see SM6 for a full set of statistics and results).

### Effects of Task-Relevance and Valence on WM Performance in Psychologically Healthy Individuals (Hypotheses A1 and A2 and Their Interaction)

For WM accuracy there were significant moderating effects of valence, *k* = 385, *d̂* = 0.14, 95% CI [0.10, 0.19], *SEM* = 0.02, *p* ≤ .0001, *Q*_*M*_(1) = 38.29, *p* ≤ .0001; and task-relevance, *k* = 391, *d̂* = −0.24, 95% CI [−0.40, −0.07], *SEM* = 0.08, *p* = .004, *Q*_*M*_(1) = 8.15, *p* = .004. The valence effect was due to positive stimuli, *k* = 117, *d̂* = 0.12, *p* = .02, having a greater enhancement effect on WM accuracy compared with negative stimuli, *k* = 268, *d̂* = 0.04, *p* = .38 (see Table S2 for full statistics). The effect of task-relevance was due to task-relevant affective targets, *k* = 257, *d̂* = 0.08, *p* = .15, improving WM performance and task-irrelevant affective distractors impairing performance, *k* = 134, *d̂* = −0.04, *p* = .55, though both effects considered alone were trivial in magnitude and neither was significant (Table S2).

These main effects on WM accuracy were qualified by a significant interaction of valence and task-relevance, *k* = 385, *d̂* = −0.52, 95% CI [−0.70, −0.33], *SEM* = 0.09, *p* ≤ .0001, *Q*_*M*_(3) = 78.62, *p* ≤ .0001. Univariate analyses (see supplemental results, SM7, for the moderating effect of valence in task-relevant and task-irrelevant stimuli separately) revealed that task-relevant targets improved WM irrespective of valence (Table 2). In contrast, negative and positive task-irrelevant distractors had opposing effects with positive distractors improving, and negative distractors impairing, performance (Table 2). However, neither of these separate effects in the context task-irrelevant distractors was significant alone.[Table-anchor tbl2]

For RT neither main effects were significant, *p* ≥ .20. Unlike WM accuracy there was no significant heterogeneity, *Q*_*M*_(3) = 6.80, *p* = .079. However, there was a significant interaction of valence and task relevance, *k* = 309, *d̂* = −0.16, 95%CI [−0.30, .03], *SEM* = 0.07, *p* = .023. Univariate analyses showed a significantly moderating effect of valence only for targets not task-irrelevant distractors (SM7). The significant effect in targets was due to significantly slowed WM RT for negative targets, which was not observed for positive targets, which showed a non-significant speeding effect (Table 2).

### Variations in Affective Significance as a Function of Mental Health Status (Hypothesis B1)

As a test of affective significance—the difference between the predicted hard prioritization afforded highly significant material versus soft prioritization (Pessoa, 2009)—we hypothesized (Hypothesis B1) that, overall, affective information will have a greater behavioral impact on WM processing in individuals suffering from mental health problems compared with psychologically healthy individuals.

**Figure 4 fig4:**
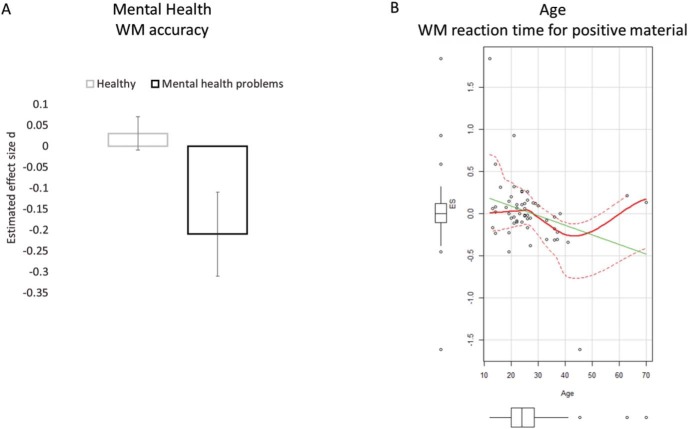
Affective significance across mental health status (A) and age (B). (A) The left panel depicts the effect sizes (*d̂*) of the difference between WM accuracy for affective compared with neutral stimuli in healthy individuals (light gray) and those suffering from mental health problems (black). (B) The right panel illustrates the association between ES = the effects size of the difference in WM RT for positive relative to neutral stimuli and age.

In line with Hypothesis B1, results showed that WM accuracy was significantly more impaired by affective material in those experiencing mental health problems compared with healthy individuals, *k* = 505, *d̂* = −0.17, 95% CI [−0.26, −0.09], *SEM* = 0.04, *p* ≤ .0001, *Q*_*M*_(1) = 17.49, *p* ≤ .0001 (Figure 4A) with affective stimuli having the predicted larger effect on WM performance in individuals suffering from mental health difficulties, *k* = 114, *d̂* = −0.21, 95% CI [−0.42, −0.01], *SEM* = 0.10, *p* = .041, *Q*_*M*_(113) = 790.96, *p* ≤ .0001, compared with healthy individuals (see results for Hypothesis A for a characterization of healthy performance).[Fig-anchor fig4]

As in the healthy individuals (see results for Hypotheses A1 and A2), the effect of affective material in those with mental health problems was moderated by main effects of valence, *k* = 114, *d̂* = 0.21, 95% CI [0.06, 0.36], *SEM* = 0.08, *p* = .006, *Q*_*M*_(1) = 7.71, *p* = .006; and task-relevance, *k* = 114, *d̂* = −0.59, 95% CI [−0.92, −0.27], *SEM* = 0.17, *p* = .0004, *Q*_*M*_(1) = 12.70, *p* = .0004. Univariate analyses showed impairing effects on WM accuracy of similar magnitude for negative (*d̂* = −0.20) and positive (*d̂* = −0.25) material, although only in the case of negative stimuli was this statistically significant (Table S3). In individuals with mental health problems, task-irrelevant distractors (*d̂* = −0.24) showed a greater impairing effect on WM accuracy compared with task-relevant targets (*d̂* = −0.05), which did not significantly impair WM accuracy (Table S3). There were insufficient studies including positive materials across the two conditions of task-relevance to investigate the interacting effects between task-relevance and valence.

There was no effect of mental health status for WM RT, *k* = 409, *d̂* = 0.08, 95% CI [−0.06, 0.08], *SEM* = 0.04, *p* = .774, *Q*_*M*_(1) = 0.08, *p* = .774 nor was there a moderating effect of valence or task relevance, *k* = 95, *p*’s > .114.

### Variations in Affective Significance Across Age (Hypothesis B2)

A second source of variation in affective significance is age, with the age-related positivity effect in attention and memory (Scheibe & Carstensen, 2010) leading to the prediction that with increasing age individuals become better at processing positive information in WM. WM accuracy showed small positive associations with age for both negative, *r*(143) = .17, 95% CI [0.00, 0.32], *p* = .045; and positive stimuli, *r*(73) = .15, 95% CI [−0.08, 0.37], *p* = .186, with older individuals remembering more affective relative to neutral material. For WM RT there was a small to moderate size significant association with RT for positive relative to neutral stimuli decreasing across age, *r*(53) = −.31, 95% CI [−0.53, −0.05], *p* = .023. That is, older individuals were faster to respond to WM tasks when the tasks included positive relative to neutral stimuli. There was no significant association between WM RT and age for negative stimuli, *r*(96) = .13, 95% CI [−0.07, 0.32], *p* = .202.

## Interim Discussion: The Behavioral Meta-Analysis

In line with our Hypothesis A, in psychologically healthy individuals, although WM RTs in the presence of affective, relative to neutral, stimuli were significantly slower, the effect size was trivial in magnitude (*d̂* = 0.07). We also found no significant overall effect of affective material (*d̂* = 0.03) on WM accuracy. These negligible effect sizes are in line with the DCF’s assertion that the kinds of stimuli typically employed in laboratory experiments—affective words and pictures—will be afforded low affective significance and only elicit a “soft prioritization” in the system. This will result in correspondingly minimal behavioral effects, that are modulated by other study-specific and individual-differences factors beyond affective significance such as WM load, age, and the nature of the affective stimuli (e.g., words, vs. images) and interactions between them (King & Schaefer, 2011; Mano et al., 2013; Mikels, Larkin, Reuter-Lorenz, & Cartensen, 2005; Rypma & D’Esposito, 2000; Sander, Lindenberger, & Werkle-Bergner, 2012; Scheibe & Carstensen, 2010). Previous work showed opposing effects of affective distractors compared with task-relevant information on a range of cognitive processes (for a review, see Dolcos et al., 2017). In line with this work we predicted that study-specific sources of variation would be affective stimuli task-relevance and valence.

### Task-Relevance Interacts With the Valence of Affective Stimuli to Impact on WM Performance

There were small significant effects of valence and task-relevance on WM accuracy in line with our Hypotheses A1 and A2. The valence effect was due to positive stimuli enhancing WM accuracy compared with negative stimuli. The effect of task-relevance was due to task-relevant affective targets improving WM performance while task-irrelevant affective distractors impaired WM performance. Importantly, there was also a moderate to large significant interaction between task-relevance and valence on WM accuracy, with task-irrelevant positive and negative distractors having no significant effects on WM accuracy (but in opposite directions), while task-relevant affective (irrespective of valence) targets significantly improved WM performance, although effects were small. Interestingly, in the absence of accuracy effects there was a trivial to small slowing effect of negative targets on WM RT. These facilitation effects suggest that relative to neutral targets, task-relevant affective targets may confer a small advantage in terms of perceptual competition. The neuroimaging meta-analysis may further elucidate this point if affective information does show a related activation increase within the brain’s attention network.

The well-documented affective enhancement effect in long-term memory is proposed to be associated with enhanced early encoding of the affective memory trace, which is then consolidated over time (Murty, Ritchey, Adcock, & LaBar, 2010). The mediation model of emotional memory (Talmi, Schimmack, Paterson, & Moscovitch, 2007) argues that the mnemonic enhancement effect is the product of three types of interrelated and interacting processes: first, the above noted prioritizing of affective information within the context of limited attentional resources (Pourtois, Schettino, & Vuilleumier, 2013; Vuilleumier, 2005); second distinctiveness, the notion that encoding of affective information is prioritized because affective relative to neutral information stands out (cf. the notion of “impact”; Ewbank, Barnard, Croucher, Ramponi, & Calder, 2009); and finally, shared thematic links (organization), which Talmi, Schimmack, Paterson, and Moscovitch (2007) argue are more easily formed between affective compared with neutral information further assisting memory encoding. These processes could similarly account for the small affective advantage observed for task-relevant affective material here in WM and could usefully be systematically explored in future research.

The small, nonsignificant enhancing effect (*d̂* = 0.20) of positive distractors and the negligible impairing effect of negative distractors (lower accuracy *d̂* = −0.07, slowed WM RT *d̂* = 0.11) suggest that competition for perceptual or executive resources from affective distractors do not markedly affect WM performance over and above that of neutral distractors. However, the nonsignificant enhancement effect of positive distractors needs to be considered in the context of the small number of effect sizes (*k* = 28) from 13 studies contributing to this effect. If this small enhancing effect does replicate across a larger number of future studies, it could arguably be interpreted as reflecting the motivational impact of positive information (H. Yang, Yang, & Isen, 2013). Specifically, one could argue that positive stimuli related to reward and motivations of affiliation may focus executive processes due to the increased—relative to neutral—motivational salience of the context in which WM is engaged (Stussi, Pourtois, & Sander, 2018). To further explore the role of motivational salience in WM and executive control more broadly, careful consideration should be given to the nature of the positive and negative stimuli used in research. The type of stimuli should be theory-driven and tap into affective concerns relevant to the study population under investigation (e.g., social stimuli in adolescence; Mueller, Cromheeke, Siugzdaite, & Boehler, 2017; or negative self-referential processing in depression Schweizer et al., 2018) and the construct under investigation (e.g., survival relevance; Lindström & Bohlin, 2012).

### Affective Significance

We hypothesized that the effect of affective stimuli on WM performance would vary as a function of their affective significance. Affective significance was proposed to vary as a function of both mental health status (Hypothesis B1) and age (Hypothesis B2).

#### Mental health status

Supporting Hypothesis B1, we found a significantly greater effect of affective relative to neutral material on WM accuracy in individuals suffering from mental health problems (*d̂* = −0.21) compared with healthy individuals (*d̂* = 0.03). In individuals suffering from mental health problems performance was impaired relative to neutral by both negative (*d̂* = −0.20) and positive stimuli (*d̂* = −0.25). Though the effect was significant only for negative stimuli. The lack of significance for positive material may reflect a power issue as only 26 effect sizes were included. Showing that both positive and negative information have an effect of similar magnitude highlights the importance of recent developments toward the investigation of hedonic processing and reward learning in individuals with psychopathology (e.g., Admon & Pizzagalli, 2015; Husain & Roiser, 2018) to complement research into the processing of negative information. The relatively greater impairment in WM performance for affective relative to neutral material is remarkable considering that this is over and above the substantial impairments in performance on affectively neutral task measures of executive functions (including WM) found in most types of mental health problems. For example, compared with healthy individuals those with depression (*d̂* = 0.32–0.97; Snyder, 2013), attention-deficit and hyperactivity disorder (*d̂* = 0.60–0.89; Boonstra, Oosterlaan, Sergeant, & Buitelaar, 2005), and posttraumatic stress disorder (*d̂* = 0.46–0.62; Scott et al., 2015) show moderate to large impairments in executive functioning in tasks populated with neutral material.

In individuals suffering from mental health problems there was a significant effect of task-relevance, with task-irrelevant distractors having a greater impairing impact (*d̂* = −0.24) compared with task-relevant targets (*d̂* = −0.05). This effect of task relevance in those with mental health difficulties is in line with theories emphasizing the importance of attentional control with respect to cognitive vulnerabilities to mental health problems. Reduced inhibition of negative, especially threat-related, information in anxiety is likely to account for increased attentional resources drawn to the affective distractors that become unavailable to task-relevant processing (Bar-Haim et al., 2007; Derakshan & Eysenck, 2009; Reinholdt-Dunne, Mogg, & Bradley, 2013). In depression, the inability to disengage attention from affective distractors may similarly limit the attentional resources available to processing task-relevant information in WM (De Raedt & Koster, 2010; Everaert, Koster, & Derakshan, 2012). Affective WM tasks then may be sensitive transdiagnostically to individual differences in mental health status. While attentional control capacity has been shown to be predictive of the onset of depressive and anxiety symptoms prospectively (Kertz, Belden, Tillman, & Luby, 2016), little is known about the development of attentional control over affective information specifically (Peterson & Welsh, 2014; Prencipe et al., 2011), which may identify those at risk for mental health problems across a range of disorders.

##### Disorder-specific variation

A cautionary note is warranted when interpreting these findings of course because, as with psychologically healthy individuals, tests of heterogeneity for all of these effects in individuals with mental health problems were significant. It is worth rehearsing two caveats related to using mental health status as a proxy for affective significance that may partly account for this heterogeneity. First, the status “mental health” here included a wide range of mental health problems (including schizophrenia, attention deficit and hyperactivity disorder, depression, and posttraumatic stress disorder). Second, the impact of affective material is potentially and likely not uniform across this diversity of mental health conditions or across other syndrome-specific sources of variation such as phase of the syndrome (e.g., acute vs. remitted), although these remain empirical questions.

#### Age

The effects discussed above were limited to WM accuracy. The moderating effect of age, however, was strongest on WM RTs with effects on accuracy being trivial-to-small and unreliable. The RT results showed that, with increasing age, individuals respond more quickly on WM tasks in the context of positive information (*r* = −.31) with this effect being nonsignificant in the reverse direction in the context of negative information (*r* = .15). This is in line with the positivity effect that characterizes socioemotional selectivity theory (Carstensen, 2006), whereby older adults preferentially process positive information due to age-related motivational shifts (Mather, 2016; Mather & Carstensen, 2005). Kensinger (2008) interestingly showed that the age-related positivity effect may be particularly marked for low arousing material, whereas items high in arousal hijack attentional resources irrespective of valence. This argument is also in line with Labouvie-Vief, Grühn, and Studer’s (2010) equilibrium model, which argues that with increasing age the spectrum of acceptable emotions shrinks, in particular for negative emotions. As noted above, with one exception, all of the positive stimuli included in the current meta-analysis are arguably low in arousal and may therefore be particularly sensitive to the age-related positivity effect.

We turn next to the neuroimaging review and revisit the results of this behavioral meta-analysis in the General Discussion in light of the results of imaging data synthesis.

### Neuroimaging Meta-Analysis

**Figure 5 fig5:**
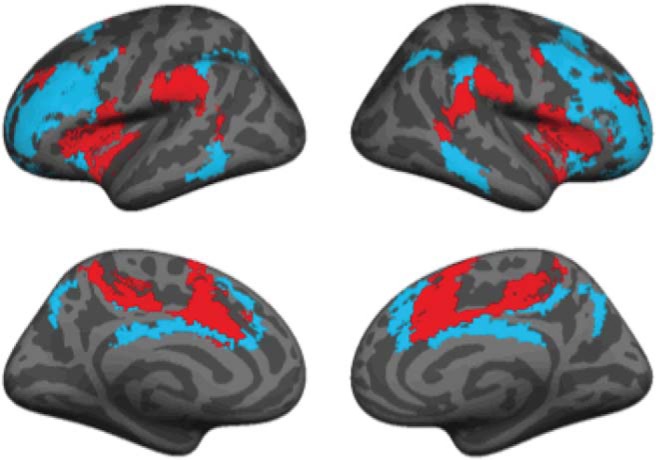
Fronto-parietal control network (blue) and salience network (red).

Functional neuroimaging studies and research in lesion patients have provided good evidence for the involvement of a fronto-parietal control network (Figure 5) in WM and other higher-order cognitive functions such as fluid intelligence (for reviews, see Duncan, 2006, 2010; Nee et al., 2013). Specifically, neural models of WM capacity have implicated this network in the active maintenance of representations and goal-states in WM, and in the control of task-related attention (Constantinidis & Klingberg, 2016; Duncan & Owen, 2000; Miller, 2000; Miller & Cohen, 2001; Nee et al., 2013; Nee, Wager, & Jonides, 2007; Owen et al., 2005; Postle, 2016). The major nodes of the frontoparietal control network include the bilateral dorsolateral prefrontal cortex (dlPFC) and the inferior parietal lobe.[Fig-anchor fig5]

In addition to the fronto-parietal network (e.g., Coull, Frith, Frackowiak, & Grasby, 1996; Vincent, Kahn, Snyder, Raichle, & Buckner, 2008), WM, especially in the presence of affective information, may recruit portions of the so-called salience network and ventral attention network (Barrett & Satpute, 2013; Corbetta, Patel, & Shulman, 2008; Eckert et al., 2009; Seeley et al., 2007; see Figure 5), which include nodes in the anterior cingulate cortex (ACC), anterior insula, and amygdala (Seeley et al., 2007; Wilson-Mendenhall, Barrett, & Barsalou, 2013). Both of these networks are predicted by the theories reviewed above to be sensitive to the perceptual and executive competition created by affective (relative to neutral) stimuli in WM tasks (e.g., Pessoa, 2008, 2009).

### Neural Substrates of Affective WM Processing

The hypothesized involvement of the *amygdala* during affective WM is in line with research showing that attentional capture from affective information (e.g., Pessoa & Ungerleider, 2004) is associated with increased activation of the amygdala (LeDoux, 2012; Öhman, Flykt, & Esteves, 2001; Vuilleumier, 2005; Vuilleumier & Huang, 2009). The interactive connections of the amygdala with sensory processing regions show that these effects appear early in processing and implicate the amygdala in the biasing of processing toward affective salience at a preconscious stage (Pessoa, 2005; Phelps, 2006; Whalen & Phelps, 2009).

This prioritized processing of affective information reliably shows greater recruitment of *visual brain areas* for affective compared with neutral stimuli (Sabatinelli et al., 2011; Satpute et al., 2015), irrespective of whether the stimuli are attended or unattended (for reviews, see Tamietto & de Gelder, 2010; Vuilleumier, 2005; Vuilleumier & Huang, 2009).

The elicited affective experience in turn may engender affect-regulatory processes that recruit components from the fronto-parietal control network (for reviews, see Buhle et al., 2014; Kalisch, 2009). Models of affect regulation have implicated the ventrolateral node (i.e., inferior frontal gyrus) of the fronto-parietal control network in the regulation of affective responses as the neural substrate of processes involved in the selection of alternative semantic interpretations of the affective material, as well as of more generic inhibitory processes (Dillon & Pizzagalli, 2007; Elliott & Deakin, 2005; Ochsner, Silvers, & Buhle, 2012). Cognitive control models of emotion regulation also implicate dorsal nodes of the fronto-parietal control network, including the dlPFC, posterior PFC and inferior parietal regions, because of their likely role underpinning the directing of selective attention and the updating of WM (Ochsner et al., 2012). Given these regions’ involvement in WM per se, however, it seems unlikely that they will be more activated during the processing of affective material relative to neutral information. Indeed, we suggest below that the opposite (greater involvement for neutral over affective) may be the case.

### Neural Substrates of WM Processing of Affectively Neutral Information Relative to Affective Information

At the neural level, the theories of emotion-cognition interactions do not offer a specific prediction regarding this “reverse contrast”—the neural substrates that are recruited more frequently during WM tasks in the presence of neutral versus affective information. The DCF does however predict that, through perceptual and executive competition, affective information draws resources away from task-related processing. Consequently, it seems plausible that task-relevant brain regions should be recruited more frequently in the absence of affective material. In the case of WM, these task-related regions include the more dorsal regions of the fronto-parietal network, especially the dlPFC (Nee et al., 2013). This putative dissociation between the ventral and the dorsal streams of the fronto-parietal control network is supported by recent reviews of the neuroimaging literature showing greater involvement of the dorsal stream of the network for neutral compared with affective distractor material presented in executive functioning tasks, including WM tasks (Iordan & Dolcos, 2017; Okon-Singer et al., 2015). In contrast, and in line with our hypotheses derived from the DCF, affective distractors are associated with more frequent recruitment of the ventral stream of the network (Iordan & Dolcos, 2017; Okon-Singer et al., 2015).

To summarize, we hypothesized that:

*Hypothesis C:* Compared with the processing of neutral stimuli, the processing of affective stimuli would be associated with more frequent activation within the visual cortices, portions of the salience network, including the amygdala, and the ventrolateral prefrontal node within the fronto-parietal control network.

*Hypothesis D:* And that the reverse contrast—differential activation when processing neutral compared with affective stimuli (neutral > affective)—would be associated with more frequent neural activation within task-related regions in the dorsal components of the fronto-parietal control network.

In line with the behavioral analyses, we explored the neural correlates of affective versus neutral stimuli’s task-relevance.^8^ However, we were unable to explore the effects of valence or interactions between valence and task-relevance within the fMRI data because insufficient neuroimaging studies included these contrasts. Moreover, it was not possible to explore the neural correlates of the effects of affective significance because the few studies which reported neuroimaging data for individuals with mental health problems ranged across various disorders that arguably present with both overlapping and distinct anatomical and functional anomalies (Davidson et al., 2002; Dickstein, Bannon, Castellanos, & Milham, 2006; Elzinga & Bremner, 2002; Menon, 2011; Shenton, Dickey, Frumin, & McCarley, 2001), thus precluding useful data synthesis at this stage.

## Methods for the Imaging Meta-Analysis

### Identification and Screening of Studies

The identification and screening stages were conducted in tandem with the behavioral meta-analysis according to PRISMA guidelines.

### Eligibility

We checked the full-texts of the identified studies to ascertain whether they reported fMRI data associated with the effects of affective material on WM in healthy individuals (Table 1). To be included, studies had to report functional imaging contrasts comparing neutral and affective information. Specifically, we included the contrasts examining regions showing greater activation for neutral versus affective stimuli during WM task performance and the reverse contrast identifying regions that reported greater activation for affective versus neutral stimuli during a WM task (for reasons for exclusion see SM8). The studies had to report BOLD response data on these contrasts either across the whole brain or in specified regions of interest using normalized stereotactic spaces (i.e., Montreal Neurological Institute and Hospital [MNI] or Talairach space). For each contrast, peak activations were included that were reported in the individual studies. It should be noted here that while we tested specific anatomical hypotheses (Hypotheses C and D) about the correlates of WM tasks including affective versus neutral information, the imaging meta-analytic approach we adopted was agnostic to these hypotheses and conducted across the whole brain.

### Analytic Approach

We coded contrasts based on the affective qualities of the stimuli (e.g., affective vs. neutral, neutral vs. affective) and on the task-relevance (i.e., task-relevant targets vs. task-irrelevant distractors). Based on these codes, we computed multikernel density maps (procedures described below) that corresponded with the behavioral analysis. First, we examined the brain regions that were frequently engaged during affective versus neutral stimulus conditions (28 contrasts), and neutral versus affective stimulus conditions (19 contrasts). To investigate differential effects of affective material depending on task-relevance of the affective stimuli, multikernel density analysis (MKDA) maps were calculated separately for task-relevant affective targets (10 contrasts) and task-irrelevant affective distractors (18 contrasts).

#### Multikernel density analysis (MDKA)

The contrast maps were submitted to a MKDA, as described in detail and validated by Wager, Lindquist, and Kaplan (2007; see Kober et al., 2008; Lindquist, Wager, Kober, Bliss-Moreau, & Barrett, 2012) and implemented in Matlab software using the NeuroElf toolbox (www.neuroelf.net). A MKDA nests activation points within contrast maps and thereby limits the undue influence of studies that report many more activation points than others. Coordinates reported in Talairach space were transformed to MNI space using the “mni2tal” estimation procedure provided by M. Brett (http://imaging.mrc-cbu.cam.ac.uk/imaging/CbuImaging). An indicator map was generated for each study contrast by setting voxels in a 10-mm sphere surrounding each reported peak activation point to 1. Contrasts were weighted by the square root of the sample size. For each voxel, a point estimate of the probability of contrasts that activated the voxel was computed. To determine significance, for each comparison a Monte Carlo simulation (5,000 iterations) was performed that preserved the number of contrasts and coordinates within contrasts, but randomly assigned the coordinate locations to gray matter regions of the brain, and for a voxel-level threshold of *p* < .01, a *k-*extent cluster-level threshold was obtained to meet a whole-brain family wise error rate (FWER) statistical correction of *p* < .05.

## Results of the Neuroimaging Meta-Analysis

### Included Studies

Of the 165 studies identified in the behavioral meta-analysis, 52 studies included fMRI data. Of these, 19 were excluded from the neuroimaging meta-analysis (see supplementary results for reasons for exclusion and the PRISMA diagram in Figure S2). The final sample included 683 participants, 456 coordinates, and 63 contrasts from 33 studies (denoted with an asterisk in the column titled “Imaging” in Table 1). See Table S5 for an overview of the specific contrasts and number of peak activation points (i.e., coordinates) included across studies. The tasks included in the analyses are described in Table 1 with the most frequently used tasks in the neuroimaging studies reviewed being *n*-back (*n* = 13) and delayed-match-to-sample (*n* = 12) tasks.

### Brain Regions Consistently Engaged During Affective Compared With Neutral Stimulus Conditions in WM Tasks (Hypotheses C and D)

**Figure 6 fig6:**
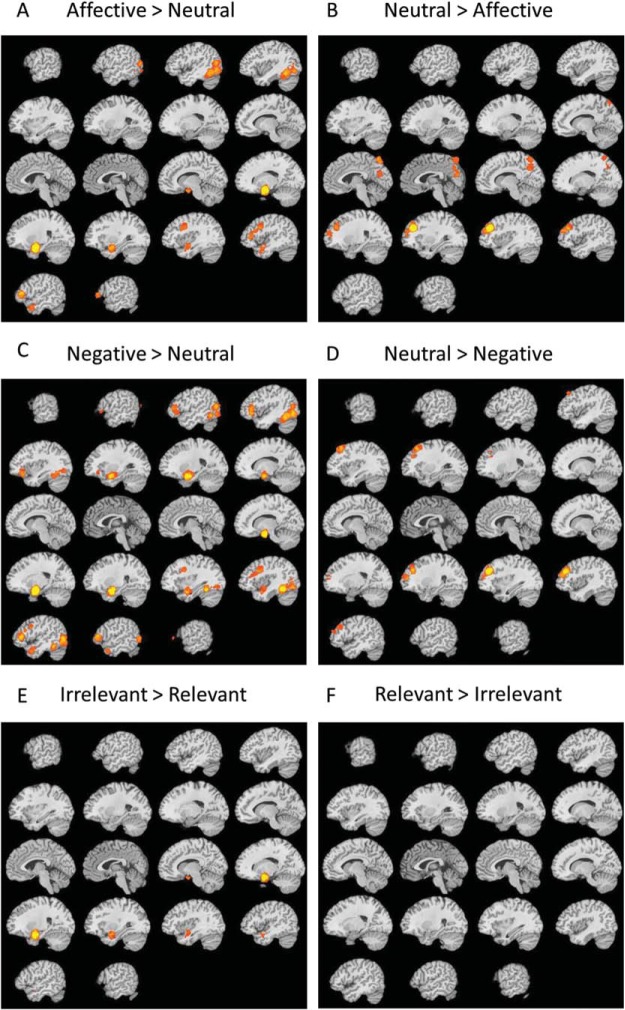
Affective stimuli as distractors or targets in WM tasks. Each panel shows brain regions that were more frequently engaged for WM contrasts comparing: (A) affective > neutral stimuli; (B) neutral > affective stimuli; (C) negative > neutral stimuli; (D) neutral > negative stimuli; (E) affective task-irrelevant distractor > affective task-relevant targets in WM tasks; and (F) task-relevant compared with irrelevant affective stimuli, for which there were no reliable activations. The color gradation in the figure indicates the frequency of recruitment of a specific region. That is, the lighter the yellow, the more frequently the region was recruited during the contrast of interest. Colored areas represent activation frequencies at *p* < .05, FWER corrected.

The neuroimaging meta-analytic results supported our first neural hypothesis (Hypothesis C) that the processing of affective stimuli during WM tasks, relative to neutral stimuli, would be associated with more frequent activation across the brain’s salience network, specifically the bilateral amygdalae, and also within the ventrolateral prefrontal cortex (Figure 6A). We also found support for our second neural hypothesis (Hypothesis D), with the contrast comparing neutral with affective stimulus material being associated with more frequent activation in brain regions commonly associated with WM task performance in the dorsal stream of the fronto-parietal control network—a large node in the right dlPFC—as well as the precuneus in the inferior parietal cortex (Figure 6B). For a full list of significant clusters comparing affective with neutral stimuli, see Table S6. Given the proposed greater affective significance of negative compared with positive stimuli (Hypothesis A1) we explored the corresponding neural effects for negative and positive stimuli separately. The contrast comparing negative with neutral stimuli (Figure 6C–D; Table 3) showed the same pattern of results as the overall affective effect. In contrast, no brain regions were significantly more recruited when comparing positive with neutral stimuli.[Fig-anchor fig6][Table-anchor tbl3]

### Task-Relevance: An Exploratory Analysis of Brain Regions More Consistently Engaged When the Affective Information Is the Task-Relevant Target Versus the Task-Irrelevant Distractor^9^

Next, we computed differences in MKDA maps to explore which neural regions were more frequently engaged during the processing of affective relative to neutral information, separately for task-relevant targets and task-irrelevant distractors. Interestingly, given the behavioral results which showed a significant effect of affective material only for task-relevant targets, the neuroimaging effects were driven by the task-irrelevant distractors. Relative to neutral distractors affective distractors more frequently activated the bilateral vlPFC, amygdalo-hippocampal complex, and left temporo-occipital lobe (including the fusiform gyrus; see Figure 6C). The reverse contrast showed greater activation in the dlPFC for neutral compared with affective distractors. For a full list of the clusters and peak activations see Table S7. There were no significant differences in MKDA maps between contrasts that included affective compared with neutral targets.

Finally, looking at only the affective conditions comparing MKDA maps for affective task-irrelevant distractors with affective task-relevant targets revealed more frequent activation of a cluster in the right temporal lobe and subcortical regions including the amygdalo-hippocampal complex, *k* = 476, maxima = .71, peak coordinate = 24/0/–24 (see Figure 6E) for task-irrelevant distractors. The reverse contrast showed no brain regions to be more significantly activated for task relevant affective target compared with irrelevant distractors.

## Interim Discussion of the Neuroimaging Meta-Analysis

The meta-analysis of the brain regions recruited during WM performance in the presence of affective compared with neutral stimuli confirmed our hypothesis (Hypothesis C) that affective stimuli would recruit regions from both the larger salience network (including the amygdalo-hippocampal complex) as well as ventral components of the fronto-parietal control network (i.e., vlPFC), in addition to regions in the temporo-occipital lobe, in particular the fusiform gyrus, a brain region involved in the processing of faces (Bressler & Menon, 2010; Ishai, 2008). In line with our second neural prediction (Hypothesis D), the MKDA map for brain regions that were more frequently activated for WM tasks performed with neutral (relative to affective) stimuli revealed two clusters in dorsal components of the fronto-parietal control network: one in the right dlPFC and a second cluster in the precuneus. Our exploratory analyses showed that contrasting regions activated more frequently in the presence of affective distractors compared with task-relevant targets yielded more frequent activation in the right amygdalo-hippocampal complex.

### Neural Substrates of Affective WM and the Dual Competition Framework

Before discussing the meta-analytic findings, a note of caution is warranted. Any interpretations of findings from neuroimaging at the level of cognitive theory are subject to the concerns surrounding reverse inference (Poldrack, 2011). In the present case, a particular contrast, for example the presence of affective compared with neutral stimulus material in WM tasks, may be associated with more frequent activation of a given brain region. At the same time, in the wider literature, a particular cognitive process (e.g., attentional capture through salience) may have been previously putatively linked to that same region in other studies. Through a process of reverse inference, evidence supporting activation of that region in the present meta-analysis could be taken to mean that that particular cognitive process is also engaged by this contrast (Poldrack, 2006). However, of course, most brain regions and networks support a multitude of cognitive functions and so any such assumptions that the implicated processes across studies or sets of studies are the same, and specific, must only be tentative. That said, it would be remiss not to interpret the present findings within the context of the wider extant literature and so we have sought an appropriate balance of informed discussion and inferential caution.

#### Affective versus neutral material

Interestingly in light of negligible behavioral effects of affective information on WM performance in psychologically healthy individuals, the neuroimaging data shows that robust effects exist at the neural level of processing. The more frequent activation evident within the amygdala and the temporo-occipital lobe including the fusiform gyrus, arguably reflect the allocation of greater processing resources toward these affective, often facial, stimuli within the included studies. The more frequent activation of the amygdala is in line with the well-documented role of the amygdala in salience processing (Adolphs, 2010; Pessoa & Adolphs, 2010; Whalen & Phelps, 2009). The involvement of the inferior temporal gyrus, which has reliably been implicated in emotion regulation (for a review, see Buhle et al., 2014) may be indicative of individuals’ affect regulatory efforts in response to affective stimuli.

Increased activation frequency observed in the vlPFC has been implicated in inhibitory processing (D’Esposito, Postle, Ballard, & Lease, 1999; E. E. Smith & Jonides, 1999; Jonides, Smith, Marshuetz, Koeppe, & Reuter-Lorenz, 1998) and has been proposed to reflect individuals capacity to cope with the greater affective responses elicited by affective relative to neutral stimuli (Denkova et al., 2010; Dolcos, Kragel, Wang, & McCarthy, 2006). Indeed, Dolcos and McCarthy (2006) showed a clear association (*r* = −.74) between vlPFC activation and individuals’ ratings of affective stimuli’s distractibility during a WM task, but not for neutral distractors *r* = .13. The vlPFC’s role in cognitive control over affective responses has also been related to the deployment of cognitively engaging affect regulatory strategies such as reappraisal (Buhle et al., 2014; Ochsner et al., 2009, 2012). The present finding may therefore also in part reflect the implicit emotion regulation of affective material that participants likely engage in when performing tasks that contain such material. These regulatory processes may be enacted more specifically through the retrieval and/or selection of relevant semantic (Badre & Wagner, 2005, 2007) or social (Satpute, Badre, & Ochsner, 2014) information.

#### Task-relevance

Our exploration of the neural substrates of a stimulus’ task-relevance revealed that task-irrelevant distractors showed greater activation frequency, relative to task-relevant targets in the vlPFC, amygdalo-hippocampal complex, and temporo-occipital complex, whereas neutral distractors recruited the dlPFC more reliably. This dissociation has been observed in reviews of the neural substrates of affective distractors included in cognitive paradigms beyond WM (Dolcos & Denkova, 2014; Iordan, Dolcos, & Dolcos, 2013) and indeed has been proposed by Dolcos and colleagues across a series of studies on the impact of affective distractors on WM (e.g., Dolcos, Diaz-Granados, Wang, & McCarthy, 2008; Dolcos & McCarthy, 2006; Iordan & Dolcos, 2017). Specifically, the review by Iordan, Dolcos, Denkova, and Dolcos (2013) noted a dissociation between what they termed a ‘cold’ dorsal executive system (including the dlPFC reported in the present meta-analysis) that was recruited for neutral over affective distractors and the “hot” ventral system that includes all the areas that showed greater activation during WM tasks, including affective compared with neutral distractors, in the present meta-analysis (i.e., vlPFC, amygdala, fusiform gyrus, and visual cortex).

More recently, Dolcos and colleagues’ dorsal executive and ventral attention systems have been linked to specific functional networks to offer a systems-level dissociation between the two (e.g., Dolcos & McCarthy, 2006; Iordan & Dolcos, 2017). In particular they highlight the dorsal executive system’s integration within the wider fronto-parietal network and the ventral attention system’s overlap with the salience network for the vlPFC and amygdala (Iordan & Dolcos, 2017). As Iordan and Dolcos (2017) note, this functional dissociation extends beyond a simplistic attribution of bottom-up processes to a ventral system and top-down executive functions to a dorsal system (cf., Pfeifer & Allen, 2012), instead emphasizing the contribution of both systems in emotion processing (e.g., showing valence specific effects in the lateral parietal cortex of the fronto-parietal network/dorsal executive system; Iordan & Dolcos, 2017; Iordan, Dolcos, & Dolcos, 2018) and control.

Finally, of the neuroimaging studies investigating the effects of task-irrelevant distractors, 60% (nine of 15) were studies including variants of the delayed-match-to-sample task (e.g., Dolcos & McCarthy, 2006). These tasks introduce a temporal latency between the distractors and memoranda (Daniel, Katz, & Robinson, 2016), which might place greater demands on executive compared with perceptual competition.

## General Discussion

Our aim with these two meta-analytic reviews was to help advance understanding of how human cognition operates in affectively laden environments, by synthesizing data on the impact of affective information on WM and the neural correlates of this effect. WM is implicated in virtually all day-to-day cognition (Barrett et al., 2004; Engle & Kane, 2004; Miyake & Shah, 1999) and much of its operation takes place in affective contexts, ranging from the overt manipulation of affective information to the performance of relatively neutral tasks in the context of affectively laden goals and plans. The studies reviewed here have tried to measure these forms of interplay by looking at WM in affective versus comparatively affect-neutral contexts within the laboratory and scanner using carefully controlled tasks.^10^ The challenge inherent in these tasks is to pursue the relatively neutral task goals while dealing with affectively laden contexts of different types as a proxy for the challenges faced in day-to-day cognition.

Our findings show that neural and behavioral data reviews and syntheses can complement each other; in this case with evidence for widespread neural engagement that arguably reflects broader cognitive engagement than the resultant behavioral data reveal (Barrett, 2009). This is a vindication of models such as Pessoa’s (2009) DCF and others (e.g., the conceptual act theory, Barrett, 2014; the model of the cognitive control of emotions, Ochsner et al., 2012) that seek to generate and integrate sets of both behavioral and neural predictions. These complementary insights from the current set of reviews further highlight the importance for future data synthesis endeavors of including, where possible, measures of behavioral performance as well as functional neuroimaging data. While this conclusion appears self-evident there is a surprising lack of meta-analytic reviews that integrate findings in this manner.

### Dissociable Behavioral and Neural Effects of Affective Information Across Task-Relevance

The dissociation between behavioral and neural findings in healthy individuals was strongest for the moderating effect of task-relevance. Interestingly, across the behavioral studies affective targets had a negligible-to-small enhancing effect on WM accuracy, whereas the effect of affective distractors was small and dependent on valence. The neuroimaging meta-analysis, however, showed that affective distractors led to more frequent recruitment of the predicted brain regions (including, amygdala, vlPFC) whereas affective targets did not. Moreover, task-irrelevant affective distractors had a greater impairing effect on WM accuracy compared with affective targets in individuals with mental health issues.

The differential behavioral and neural effects of affective stimuli on WM in healthy individuals arguably evidence the efficiency of the cognitive control system in mitigating any impact of affective information on performance. The increased recruitment of the vlPFC may reflect the organism’s effort to inhibit attention and responses toward these distractors and regulate any affective experience elicited by the distractor. This is particularly adaptive in our contemporary environments that are populated with myriads of affective distractors (e.g., phone alerts). This dissociation of behavioral and neural results observed in healthy individuals is in line with research into the interaction between affect and other types of cognition including long-term memory (Erk, von Kalckreuth, & Walter, 2010). This dissociation appears to be maintained across time (Erk et al., 2010), with behavioral memory performance for affective material being unaffected by whether individuals had been instructed to regulate their affective responses to the memoranda at encoding 12 months prior (in line with Dolcos, Labar, & Cabeza, 2005). At the neural level, however, amygdala activation during encoding of affective items that were viewed without attempts to downregulate affective experiences was stronger than amygdala activation to items encoded 12 months prior while individuals were attempting to regulate their affective responses. The reviewed evidence further suggests that it is in particular the connectivity between this vlPFC node and the amygdalo-hippocampal complex that reflects the efficacy of healthy individuals in controlling any potential interference from affective information in WM (Krause-Utz, Elzinga, Oei, Paret et al., 2014; Ladouceur et al., 2013; Ziaei, Salami, & Persson, 2017). Interestingly, Ladouceur et al. (2013) showed reduced downregulation of amygdala reactivity by the vlPFC in response to negative and positive distractors in young people with a parent suffering from bipolar disorder compared with a healthy age-matched sample. This differential pattern of neural activation across groups was observed in the absence of behavioral performance differences. Altered functional connectivity during WM performance in the presence of affective compared with neutral material may therefore constitute a sensitive marker for mental health problems before the behavioral differences that were observed in the current behavioral meta-analysis emerge. Together the studies lend support to the argument that competition for resources from affective information is being routinely resolved in the vlPFC.

In mental ill health, however, maladaptive behavioral responses and involuntary attentional engagement with affective distractors are characteristic of many disorders (e.g., anxiety disorders; Bar-Haim et al., 2007). WM performance and its neural substrates in the presence of affective distractors may therefore constitute a source of individual differences associated with mental health problems. In line with this argument, Menon’s (2011) triple neural network model of mental health proposes that weak mapping from the salience network is involved (among other things) in “[. . .] aberrant bottom-up detection of salient events, [and] aberrant control signals to other large-scale networks that facilitate access to attention and working memory resources, [. . .]” (Menon, 2011, p. 501). That is, mental health problems may be associated with particularly impaired WM performance in the presence of affective distractors due to both aberrant salience attribution to affective information at the perceptual level of competition as well as impaired control at the executive level of competition.

### Affective Significance in Mental Health and Across the Life Span

A critical prediction, although somewhat underresearched in the literature, is the impact of stimuli’s degree of affective significance on executive performance. Here we used age and mental health status as proxies for affective significance. In line with our predictions older people were faster to respond to positive material and WM performance in individuals with mental health problems was significantly impaired by affective information.

#### The changing impact of affective information on WM performance across the life span

The age results were in line with the age-related positivity effect shown in the attention and memory literature (for a meta-analytic review, see Reed, Chan, & Mikels, 2014). However, little is known about the development of WM in affective contexts from childhood through into adulthood. Of the included studies fewer than 10% (*n* = 14) were conducted in children and/or adolescents (Bertocci et al., 2014; Cromheeke & Mueller, 2016; Ladouceur et al., 2005, 2013, 2009; Mueller et al., 2015; Passarotti, Ellis, Wegbreit, Stevens, & Pavuluri, 2012, 2010, 2011; Pavuluri, Passarotti, Fitzgerald, Wegbreit, & Sweeney, 2012; Schenkel, Passarotti, Sweeney, & Pavuluri, 2012; Tavitian et al., 2014; Visu-Petra, Ţincaş, Cheie, & Benga, 2010; Z. Li et al., 2009) and there was no study of the typical development of affective WM. This is particularly surprising given that affective WM in developmental samples may provide evidence for those at risk for emotional disorders by virtue of problems with affective control capacity. Moreover, interventions that augment executive control in affective contexts may constitute efficient forms of prevention, especially when administered early in development (Wass, Porayska-Pomsta, & Johnson, 2011).

#### Pathways to competition from affective information in individuals with mental health problems

There are likely to be variations in the pathways through which the effects of affective significance create perceptual and executive competition across different mental health disorders. Arguably, differences in affective significance may exert their impact on perceptual competition in a similar way across diverse forms of psychopathology, whereas the intersection of affective significance and executive competition may rely upon different mechanisms across disorders. For example, engaging in cognitively costly emotion regulation strategies (including rumination in depression, or suppression in anxiety disorders; Aldao et al., 2010) in response to affective stimuli versus increased executive competition due to resources deployed to disambiguate affective information in schizophrenia (Kohler, Walker, Martin, Healey, & Moberg, 2010). Similarly, there are likely to be variances in the relative affective significance of the stimuli included in standard experimental paradigms across disorders. Despite these potential differences, all of the mental health disorders included in the current behavioral meta-analysis have been shown to be associated with affective dysregulation: alcohol dependence (Cheetham, Allen, Yücel, & Lubman, 2010); anxiety disorders (Cisler, Olatunji, Feldner, & Forsyth, 2010); attention-deficit and hyperactivity disorder (Graziano & Garcia, 2016; Shaw, Stringaris, Nigg, & Leibenluft, 2014); borderline personality disorder (Carpenter & Trull, 2013); mood disorders (Hofmann, Sawyer, Fang, & Asnaani, 2012; Townsend & Altshuler, 2012); obsessive–compulsive disorder (Calkins, Berman, & Wilhelm, 2013); PTSD (Frewen & Lanius, 2006); and schizophrenia (Horan, Kring, & Blanchard, 2006; Trémeau, 2006). Poor WM performance in the presence of affective material then may be a transdiagnostic marker of dysregulated affect across these disorders.

At the neural level the paucity of available studies means that the current analysis cannot speak to finer-grained questions concerning the neural substrates of the effects of affective significance across disorders. As and when further evidence emerges on affective WM from each disorder, future meta-analyses should investigate the interaction between behavioral and neuroimaging findings in these clinical populations. We currently know little about the neural substrates associated with individual differences in affective WM and potentially different pathways to interference from affective information across mental health problems. As with the posited cognitive-level pathways, the neural signatures are argued to be both overlapping and distinct across different types of mental health problems. Interestingly, the networks proposed in Menon’s (2011) triple neural network model of mental health overlap with the neural networks shown in the current meta-analysis to be associated with the effects of affective stimuli on WM performance (i.e., the salience and fronto-parietal control networks). Future research is warranted to explore the neural substrates of affective WM both within and across disorders.

### Future Directions

These behavioral and neural reviews focus on the interplay and integration between affective and cognitive processing. Here we offer some suggestions for potential next steps in this endeavor. A primary aim, we submit, should be to refine and provide empirical evaluation of neurobehavioral models of cognitive functioning in both intrinsic (e.g., affective states) and/or extrinsic (e.g., facial expression) affective contexts. Empirical support of, or challenges to, these models are currently typically offered by experimental tasks performed in laboratory settings, such as the affective WM tasks reviewed here. Empirical evidence for the influence of affective material in the real world, however, is scarce. That said, preliminary, yet critical, attempts have recently been made to embed and relate the findings from these laboratory measures to exerting affective control in everyday environments (Pe, Brose, Gotlib, & Kuppens, 2016; Pe, Raes, Koval et al., 2013; Pe, Raes, & Kuppens, 2013; Quinn & Joormann, 2015a, 2015b). For example, in an experience sampling study (*N* = 95), Pe, Koval, and Kuppens (2013) showed that affective WM updating ability predicted individuals’ ability to down-regulate high-arousal negative affective states (e.g., experiencing anger), but not low-arousal negative affective states (e.g., dysphoria). The study further showed differential associations between WM updating ability and self-reported tendencies to use rumination and reappraisal as emotion regulation strategies. These types of studies provide important insights into how executive control, as measured on laboratory tasks, may be sensitive to some but not all types of executive control over affective input in daily life. Similarly, the current analyses were limited in exploring only the impact of externally presented affective material. In daily life, however, executive control is often taxed and arguably impacted on by internally generated affective information (e.g., thoughts, memories). In a recent study, Iordan, Dolcos, and Dolcos (2018) show that autobiographical memories processed with an emotion-focus, compared with a context-focus, impair WM performance.

Inherently linked to the notion of embedding findings from tasks assessing executive control over affective information in our understanding of quotidian human cognition is the construct of emotion regulation. WM in affective contexts has been posited as central to contemporary models of emotion regulation (Etkin, Büchel, & Gross, 2015). A recent example stems from a meta-analysis, which showed that repetitive negative thinking, a maladaptive emotion regulation strategy (Ehring & Watkins, 2008; McEvoy, Mahoney, & Moulds, 2010) commonly observed in mood and anxiety disorders (Klemanski, Curtiss, McLaughlin, & Nolen-Hoeksema, 2017; Spinhoven, Drost, van Hemert, & Penninx, 2015), is selectively associated with difficulties in discarding task-irrelevant material from WM (Zetsche, Bürkner, & Schulze, 2018). Similarly, the current study showed that task-irrelevant distractors impair WM performance (*d̂* = −0.24), unlike task-relevant information (*d̂* = −0.05), in individuals with mental health problems.

Investigating the association between emotion regulation at diverse levels of analysis (from self-report to experience sampling in everyday life) and performance on affective WM tasks could advance our understanding of the role of higher-order cognitive control in emotion regulation and open new avenues for intervention (Engen & Kanske, 2013; Schweizer & Dalgleish, 2013). Preliminary studies have shown that training affective WM can improve individuals’ executive control over affective stimuli across executive functions (e.g., on an affective Stroop task; Schweizer, Hampshire, & Dalgleish, 2011) as well as their emotion regulation capacity (Schweizer et al., 2013). However, to optimize the success of such endeavors, we require mechanistic accounts of the role of cognitive control in mental health, beyond merely showing deficits in specific processes (Grahek, Everaert, Krebs, & Koster, 2018). In their important opinion article Grahek, Everaert, Krebs, and Koster (2018) propose that, in order to advance our understanding of the role of cognitive control in mental health, we require a multifaceted approach integrating the affective, cognitive and motivational domain rather than viewing them as separate processes merely interacting with each other.

Finally, all analyses showed considerable remaining heterogeneity. That is, the moderators included (i.e., valence, task-relevance, mental health status, age, emotion type, and WM load) accounted for only part of the variance in the effect of affective relative to neutral information on WM performance. Understanding the effects of other individual difference variables (e.g., factors that influence affective processing including gender and personality; Fischer, Kret, & Broekens, 2018; Hamann & Canli, 2004; Kret & De Gelder, 2012; Speed et al., 2015) not modeled in the current analyses will therefore constitute an important next step in elucidating the impact of affective information on WM performance. Characterizing the relation between these individual differences and the impact of affective information on cognition is especially relevant in the context of recent findings showing that self-relevant information may particularly tax executive resources (Dai, Rahman, Lau, Sook Kim, & Deldin, 2015; Hubbard, Hutchison, Hambrick et al., 2016; Hubbard, Hutchison, Turner et al., 2016; Iordan et al., 2018; though see Schweizer et al., 2018, Experiment 3).

## Conclusions

The present meta-analyses support theoretical proposals concerning the complex interplay between affective information and WM performance. Based on the current state of science, affective information has only a negligible effect on behavioral measures of WM in healthy individuals. At the neural level, however, processing affective versus neutral material during WM is associated with more frequent recruitment of the vlPFC, the amygdala, and the temporo-occipital cortex. The behavioral impact of affective information appears to be augmented in individuals for whom affective stimuli carry greater affective significance. Compared with healthy individuals, those suffering from mental health problems show a small and reliable impairment of WM accuracy in the presence of affective material and older adults show faster RTs in WM tasks including positive material. These findings suggest that investigating the impact of affective information on executive performance can provide an important window into the understanding of individuals’ cognitive functioning in affectively valenced everyday environments.

## Supplementary Material

10.1037/bul0000193.supp

## Figures and Tables

**Table 1 tbl1:** Included Study Samples and Task Descriptions

Author	*N*	Age	Data	Population	Task	Valence	Task-relevance	Imaging
Amir and Bomyea (2011)	32	31	RT	Generalized and social phobia	C. span	Negative	Task-relevant	
	30	29		Healthy				
Anticevic, Repovs, and Barch (2010)^DR^	21	25	Both	Healthy	DMTS	Negative	Task-irrelevant	Reported*
Anticevic, Repovs, Corlett, and Barch (2011)	24	37	Both	Healthy	DMTS	Negative	Task-irrelevant	
	28	36		Schizophrenia^1^				
Artuso, Palladino, and Ricciardelli (2012)^DR^	20	23	Both	Healthy	S. span	Negative and positive	Task-relevant	
Bakvis, Spinhoven, Putman, Zitman, and Roelofs (2010)^DR^	20	31	Accuracy	Healthy	*n*-back	Negative and positive	Task-irrelevant	
	19	35		Psychogenic seizures^2^				
Becerril and Barch (2011)^DR^	32	36	Both	Healthy	*n*-back	Negative and positive	Task-relevant	Reported*
	38	37		Schizophrenia^1^				
Beckwé, Deroost, Koster, De Lissnyder, and De Raedt (2014)	84	19	RT^e^	Healthy	S. span	Negative	Task-relevant	
Belham et al. (2013)	27	21	Both	Healthy	S. span	Negative and positive	Task-relevant	
	25	70		Healthy				
Beneventi, Barndon, Ersland, and Hugdahl (2007)^DR^	12	24	Imaging^d^	Healthy	*n*-back	Negative and positive	Task-relevant	Reported*
Berger et al. (2015)	12	78	Both	Healthy	*n*-back	Negative	Task-irrelevant	Reported
	12	74		Alzheimer’s disease^2^				
Bertocci et al. (2012)	23	32	Both	Depression^1^	*n*-back	Negative and positive	Task-relevant	Reported
	18	30		Bipolar disorder^1^				
	16	33		Healthy				
Bertocci et al. (2014)	22	14	Both	Mixed diagnoses with high emotional dysregulation^1^	*n*-back	Negative and positive	Task-relevant	Reported
	39	14		Mixed diagnoses with low emotional dysregulation^1^				
	24	13		Healthy				
Borella, Carretti, Grassi, Nucci, and Sciore (2014)	93	69	Accuracy	Healthy	C. span	Negative and positive	Task-relevant	
	63	26		Healthy				
Borg, Leroy, Favre, Laurent, and Thomas-Antérion (2011)^DR^	28	53	Accuracy	Healthy	S. span	Negative	Task-relevant	
	14	81		Alzheimer’s disease^2^				
Brunyé, Howe, Walker, and Mahoney (2013)^DR^	13	20	Both	Healthy	DMTS	Negative	Task-irrelevant	
Buratto, Pottage, Brown, Morrison, and Schaefer (2014)	40	24	Both	Healthy	*n*-back	Negative medium and high intensity	Task-irrelevant	
	40	20		Healthy				
Burhan et al. (2016)	10	73	Both	Mild cognitive impairment^2^	DMTS	Negative	Task-irrelevant	Reported
	12	66		Healthy				
Chuah et al. (2010)	24	22	Both	Healthy	DMTS	Negative	Task-irrelevant	Reported*
	24	22		Sleep-deprived^2^				
Colligan and Koven (2015)	93	19	RT	Healthy	S. span	Negative and positive	Task-relevant	
Cromheeke, Herpoel, and Mueller (2014)	17	20	Both	Childhood abuse^1,a^	DMTS	Negative and positive	Task-relevant	
	17	20		Childhood stressors^b^				
	17	20		Healthy				
Cromheeke and Mueller (2016)^DR^	33	14	Both	Healthy	*n*-back	Negative and positive	Task-relevant	
	37	21		Healthy				
Dai, Rahman, Lau, Sook Kim, and Deldin (2015)	34	19	RT	Healthy	*n*-back	Negative and positive	Task-relevant	
	33	19		Dysphoric				
De Lissnyder, Koster, and De Raedt (2012)	37	20	RT^e^	Healthy	S. span	Negative	Task-relevant	
De Lissnyder et al. (2012)	20	45	RT^e^	Healthy	S. span	Negative	Task-relevant	
	20	40		Depression^1^	S. span	Negative	Task-relevant	
De Lissnyder et al. (2012)^DR^	50	19	RT^e^	Healthy	S. span	Negative	Task-relevant	
Demeyer, De Lissnyder, Koster, and De Raedt (2012)	30	47	RT^e^	Depression remitted^1, c^	S. span	Negative	Task-relevant	
Denkova et al. (2010)	18	23	Accuracy	Healthy	DMTS	Negative	Task-irrelevant	Reported*
Diaz et al. (2011)^DR^	17	24	Both	Healthy	DMTS	Negative	Task-irrelevant	Reported*
	11	33		Schizophrenia^1^				
Doallo, Holguín, and Cadaveira (2006)^DR^	10	26	Both	Healthy	DMTS	Negative	Task-irrelevant	
Döhnel et al. (2008)^DR^	16	63	Both	Healthy	*n*-back	Negative and positive	Task-relevant	Reported*
	16	63		Mild cognitive impairment^2^				
Dolcos and McCarthy (2006)^DR^	18	22	Accuracy	Healthy	DMTS	Negative	Task-irrelevant	Reported*
Dolcos, Kragel, Wang, and McCarthy (2006)	15	22	Accuracy	Healthy	DMTS	Negative	Task-irrelevant	Reported*
Dolcos, Diaz-Granados, Wang, and McCarthy (2008)	14	25	Accuracy	Healthy	DMTS	Negative	Task-irrelevant	Reported*
Dolcos et al. (2013)^DR^	17	27	Accuracy	Healthy	DMTS	Negative	Task-irrelevant	Reported*
Edelstein (2006)	255	—	Accuracy	Healthy	C. span	Negative and positive (mixed) and attachment-related words	Task-relevant	
Erk, Kleczar, and Walter (2007)	12	25	Both	Healthy	Sternberg	Negative	Task-irrelevant	Reported*
Evans, Craig, Oliver, and Drobes (2011)	24	30	RT	Smokers	*n*-back	Smoking cues	Task-relevant	
	16	28		Healthy				
Fairfield, Mammarella, and Di Domenico (2015)^DR^	40	22	Accuracy	Healthy	S. span	Negative and positive	Task-relevant	
Fairfield et al. (2015)	35	25	Both	Healthy	*n*-back	Positive	Task-relevant	
	35	70		Healthy				
Fales, Becerril, Luking, and Barch (2010)^DR^	29	36	Both	Healthy	*n*-back	Negative and positive	Task-relevant	Reported*
Ferré (2002)	21	22	Accuracy	Healthy	S. span	Negative and positive (mixed)	Task-relevant	
García-Pacios, Del Río, and Maestú (2014)^DR^	34	22	Accuracy	Healthy	DMTS	Negative	Task-irrelevant	
García-Pacios, Del Río, Villalobos, Ruiz-Vargas, and Maestú (2015)^DR^	30	21	Both	Healthy	DMTS	Negative and positive	Task-irrelevant	
	43	22		Healthy				
	26	21		Healthy				
García-Pacios et al. (2015)^DR^	15	20	Accuracy	Healthy	DMTS	Negative and positive	Task-irrelevant	
Gathmann, Pawlikowski, Schöler, and Brand (2014)	194	27	Both	Healthy	*n*-back	Negative and positive	Task-relevant	
Giles et al. (2015)	36	20	Both	Healthy	DMTS	Negative	Task-irrelevant	
	36	20		Healthy				
Gläscher, Rose, and Büchel (2007)^DR^	23	24	Both	Healthy	*n*-back	Negative	Task-relevant	Reported*
González-Garrido, López-Franco, Gómez-Velázquez, Ramos-Loyo, and Sequeira (2015)	20	26	Both	Healthy	S. span	Negative and positive	Task-relevant	
Gonzalez-Garrido et al. (2007)	14	29	Both	Healthy	DMTS	Negative and positive	Task-irrelevant	
Gooding and Tallent (2003)	39	19	Both	Healthy	DMTS	Negative	Task-relevant	
	43	19		Social anhedonia^1,c^				
Gooding and Tallent (2004)	36	58	Both	Schizophrenia and	DMTS	Negative	Task-relevant	
				Schizoaffective disorder^1^				
	29	41		Healthy				
Goolsby, Shapiro, and Raymond (2009)	34	24	Both	Healthy	DMTS	^g^	Task-relevant	
Gotoh (2012)	26	21	Accuracy	Healthy	S. span	Negative and positive	Task-relevant	
Grecucci, Soto, Rumiati, Humphreys, and Rotshtein (2010)^DR^	12	24	Both	Healthy	DMTS	Negative and positive	Task-irrelevant	
Grimm et al. (2015)^DR^	541	46	Accuracy	Healthy	*n*-back	Negative and positive	Task-relevant	
Grimm, Weigand, Kazzer, Jacobs, and Bajbouj (2012)^DR^	20	24	Both	Healthy	*n*-back	Negative and positive	Task-relevant	Reported*
Hadley and MacKay (2006)	28	20	Accuracy	Healthy	S. span	Negative	Task-relevant	
Han et al. (2016)	20	26	Both	Obsessive-compulsive disorder	DMTS	Negative	Task-irrelevant	Reported*
	21	23		Healthy				
Hubbard, Hutchison, Hambrick, and Rypma (2016)^DR^	53		Accuracy	Healthy	C. span	Negative	Task-irrelevant	
	31			Dysphoric^1, c^				
Hubbard et al. (2016)^DR^	45		Accuracy	Healthy	C. span	Negative	Task-irrelevant	
	30			Dysphoric^1, c^				
Iordan, Dolcos, Denkova, and Dolcos (2013)^DR^	36	23	Accuracy	Healthy	DMTS	Negative	Task-irrelevant	Reported*
Joormann, Levens, and Gotlib (2011)	27	38	RT	Healthy	S. span	Negative and positive	Task-relevant	
	26	47		Depression^1^				
Kellermann et al. (2012)	36	25	Accuracy	Healthy	S. span	Negative and positive	Task-irrelevant	Reported*
Kensinger and Corkin (2003)	178	24	Both	Healthy	+	Negative and positive	Task-relevant	
Kerestes et al. (2012)	20	36	Both	Healthy	*n*-back	Negative and positive	Task-relevant	Reported*
	19	34		Remitted depression^1, c^				
Kessel et al. (2016)	23	22	Both	Healthy	*n*-back	Negative and positive	Task-relevant	
King and Schaefer (2011)	73	22	Both	Healthy	DMTS	Negative	Task-irrelevant	
Kopf, Dresler, Reicherts, Herrmann, and Reif (2013)	30	24	Accuracy	Healthy	*n*-back	Negative and positive	Task-relevant	
Koster, De Lissnyder, and De Raedt (2013)^DR^	30	21	RT^e^	Healthy	S. span	Negative	Task-relevant	
	40	19		Healthy	S. span	Negative	Task-relevant	
Krämer, Mohammadi, Doñamayor, Samii, and Münte (2010)	17	28	Imaging	Healthy	DMTS	Negative	Task-relevant	Reported*
Krause-Utz et al. (2012)	22	28	Both	Borderline PD^1^	Sternberg	Negative	Task-irrelevant	Reported*
	22	27		Healthy				
Krause-Utz et al. (2014)	28	31	Both	Borderline PD^1^	Sternberg	Negative	Task-irrelevant	
	28	31		Healthy				
Ladouceur et al. (2005)	17	12	Both	Anxiety disorders^1^	*n*-back	Negative and positive	Task-irrelevant	
	16	15		Depression^1^				
	24	13		Anxiety and depression^1^				
	18	12		Healthy				
Ladouceur et al. (2009)	26	16	Both	Healthy	*n*-back	Negative and positive	Task-irrelevant	
	31	15		High trait anxiety^1^				
Ladouceur et al. (2013)	16	14	Both	High risk bipolar disorder^1^	*n*-back	Negative and positive	Task-irrelevant	Reported*
	15	14		Healthy				
Laier, Schulte, and Brand (2013)	28	26	Both	Healthy	*n-*back	Negative, positive high arousal and positive low arousal	Task-relevant	
Lamm, Pine, and Fox (2013)^DR^	32	20	Both	Healthy	AX-CPT	Negative	Task-irrelevant	
LeMoult, Carver, Johnson, and Joormann (2015)	167	18	Accuracy	Healthy	C. span	Negative	Task-relevant	
Levens and Gotlib (2010)	29	37	Both	Healthy	*n-*back	Negative and positive	Task-relevant	
	29	42		Depression^1^				
Levens and Gotlib (2012)	40	—	Both	Healthy	*n*-back	Negative and positive	Task-relevant	
Levens and Gotlib (2015)	24	37	Both	Healthy	*n*-back	Negative and positive	Task-relevant	
	23	41		Remitted depression^1, c^				
Li, Li, and Luo (2006)^DR^	15	22	Both	Healthy	*n*-back	Negative	Task-irrelevant	
Li et al. (2009)	23	15	Imaging	Healthy	*n*-back	Negative	Task-irrelevant	Reported*
	33	15		Prenatal cocaine exposure^2^				
Lim, Bruce, and Aupperle (2014)	38	31	Accuracy	Healthy	DMTS	Negative	Task-relevant	
Lindström and Bohlin (2011)	55	24	Accuracy	Healthy	*n*-back	Negative and positive	Task-relevant	
Luciana, Burgund, Berman, and Hanson (2001)	19	22	Accuracy	Healthy	DMTS^h^	Negative	Task-relevant	
Luksys et al. (2015)	1,239	—	Accuracy	Healthy	S. span	Negative and positive (DR)	Task-relevant	
	526	22		Healthy				
MacLean, Nichols, LeBreton, and Wilson (2016)	17	27	Accuracy	Healthy	DMTS	Positive	Task-irrelevant	
MacNamara, Ferri, and Hajcak (2011)	45	—	Both	Healthy	S. span	Negative	Task-irrelevant	
MacNamara, Schmidt, Zelinsky, and Hajcak (2012)^DR^	16	—	Accuracy	Healthy	S. span	Negative	Task-irrelevant	
MacNamara and Proudfit (2014)^DR^	35	24	Accuracy	Healthy	S. span	Negative	Task-irrelevant	
	71	24		Generalized anxiety disorder^1^				
Mammarella et al. (2012)	22	46	Accuracy	Schizophrenia	C. span	Negative and positive	Task-relevant	
	22	44		Healthy				
Mammarella, Borella, Carretti, Leonardi, and Fairfield (2013)^DR^	35	25	Accuracy	Healthy	C. span	Negative and positive	Task-relevant	
	37	65		Healthy				
	37	78		Healthy				
Mammarella et al. (2016)	91	21	Accuracy	Healthy	C. span	Negative and positive	Task-relevant	
	121	20		Healthy				
Mano et al. (2013)	60	22	RT	Healthy	DMTS	Negative and positive	Task-irrelevant	
	29	19		Healthy				
Marx et al. (2011)	40	25	Both	Healthy	*n*-back	Negative	Task-irrelevant	
	39	29		ADHD^1^				
Marx, Krause, Berger, and Häßler (2014)^DR^	30	30	Both	Healthy	*n*-back	Negative	Task-irrelevant	
	16	33		Alcohol dependence^1^				
	22	28		ADHD^1^				
Mather et al. (2006)^DR^	46	18	Both	Healthy	S. span	Negative	Task-relevant	Reported*
Meule, Skirde, Freund, Vögele, and Kübler (2012)	56	24	Both	Healthy	*n-*back	Positive	Task-relevant	
Mikels, Larkin, Reuter-Lorenz, and Cartensen (2005)	40	48	Accuracy	Healthy	*n-*back	Negative and positive	Task-relevant	
Mikels, Reuter-Lorenz, Beyer, and Fredrickson (2008)^DR^	64	20	Accuracy	Healthy	*n-*back	Negative	Task-relevant	
Mitchell, Mather, Johnson, Raye, and Greene (2006)	19	21	Accuracy	Healthy	S. span	Negative	Task-relevant	Reported*
Moon and Jeong (2015)	18	37	Both	Generalized anxiety disorder	DMTS	Negative	Task-irrelevant	Reported
	18	37		Healthy				
Moreno et al. (2015)	20	27	RT^e^	Healthy	S. span	Negative	Task-relevant	
	20	32		Depression^1^				
	20	27		Healthy after tDCS^2^				
	20	32		Depression after tDCS^1^				
Morey et al. (2009)^DR^	20	34	Accuracy	Healthy	DMTS	Negative	Task-irrelevant	Reported*
	22	34		PTSD^1^				
Morey et al. (2011)^DR^	20	37	Both	Healthy				Reported
	22	31		PTSD^1^				
Mueller et al. (2015)^DR^	22	13	Both	Healthy	DMTS	Negative	Task-relevant	
	33	12		Anxiety disorder				
Mullin et al. (2012)	22	32	Both	Bipolar disorder^1^	*n-*back	Negative and positive	Task-irrelevant	Reported
	19	33		Healthy				
Neta and Whalen (2011)^DR^	18	19	Both	Healthy	*n-*back	Negative and positive	Task-relevant	Reported*
Noreen and Ridout (2010)^DR^	22	23	Accuracy	Healthy	DMTS	Negative and positive	Task-relevant	
	29	23		Dysphoric^1^				
Oei, Tollenaar, Spinhoven, and Elzinga (2009)	27	20	Both	Healthy	Sternberg	Negative	Task-irrelevant	
	27	22		Hydrocortisone administration^2^				
Oei, Tollenaar, Elzinga, and Spinhoven (2010)	27	20	Both	Healthy	Sternberg	Negative	Task-irrelevant	
	27	20		Propranolol administration^2^				
Oei et al. (2012)	16	24	Both	Healthy	Sternberg	Negative	Task-irrelevant	Reported*
	16	24		Stress induction^2^				
Onraedt and Koster (2014)	45	21	Both	Healthy	*n-*back	Negative and positive	Task-relevant	
Osaka, Yaoi, Minamoto, and Osaka (2013)^DR^	26	24	Imaging^f^	Healthy	S. span	Negative and positive	Task-relevant	Reported*
Pallesen, Brattico, Bailey, Korvenoja, and Gjedde (2009)^DR^	10	25	Accuracy^i^	Healthy	*n-*back	Negative and positive	Task-relevant	Reported*
Pallesen et al. (2010)	21	25	RT	Healthy	*n-*back	Negative and positive	Task-relevant	
Park, Kim, Jeong, Chung, and Yang (2016)	15	36	Accuracy	Generalized anxiety disorder	DMTS	Negative	Task-irrelevant	Reported
Passarotti, Sweeney, and Pavuluri (2010)^DR^	19	13	Both	Healthy	*n-*back	Negative and positive	Task-relevant	Reported*
	23	13		Bipolar disorder^1^				
	14	13		ADHD^1^				
Passarotti, Sweeney, and Pavuluri (2011)	13	14	Both	Healthy	*n-*back	Negative and positive	Task-relevant	Reported
	17	14		Bipolar disorder^1^				
Passarotti, Ellis, Wegbreit, Stevens, and Pavuluri (2012)	41	14	Both	Healthy	*n*-back	Negative	Task-relevant	Reported
	16	15		Bipolar disorder^1^				
Pavuluri, Passarotti, Fitzgerald, Wegbreit, and Sweeney (2012)^DR^	15	14	Both	Healthy	*n-*back	Negative and positive	Task-relevant	Reported
	21	13		ADHD^1^				
Pehlivanoglu, Jain, Ariel, and Verhaeghen (2014)	21	71	Both	Healthy	*n-*back	Negative and positive	Task-relevant	
	21	20		Healthy				
Perlstein, Elbert, and Stenger (2002)	10	25	Imaging^d^	Healthy	S. span	Negative and positive	Task-relevant	Reported*
Phillips et al. (2011)^DR^	21	26	Both	Healthy	*n*-back	Negative and positive	Task-relevant	
	22	25		First degree relative with schizophrenia^1c^				
Prehn et al. (2013)	17	29	Both	Healthy	*n*-back	Negative	Task-irrelevant	
	15	28		Antisocial and borderline PD^1^				
Putman, Hermans, and van Honk (2007)	18	20	Accuracy	Healthy	S. span	Negative and positive	Task-relevant	
	18	20		Hydrocortisone administration^2^				
Rebetez, Rochat, Billieux, Gay, and Van der Linden (2015)	88	23	Both	Healthy	S. span	Negative and positive	Task-relevant	
Reinecke, Rinck, and Becker (2006)	23	21	Accuracy	Spider fearful^1^	S. span	Negative		
	23	21		Healthy				
Reinecke, Becker, and Rinck (2009)	23	21	Accuracy	Spider fearful^1^	S. span	Negative		
	24	22		Healthy				
	18	21		Spider fearful^1^				
	19	21		Healthy				
Reinecke, Soltau, Hoyer, Becker, and Rinck (2012)^DR^	29	31	Accuracy	Spider fearful^1^	S. span	Negative		
Richter et al. (2013)	18	24	Both	Healthy	*n*-back	Negative	Task-relevant	Reported
	16	24		Healthy				
Robinaugh, Crane, Enock, and McNally (2016)	30	26	Both	Healthy	S. span	Negative and positive	Task-relevant	
	30	26		Healthy				
Román et al. (2015)^DR^	51	20	Both	Healthy	*n*-back	Negative and positive	Task-relevant	
	52	20		Healthy				
Sabharwal et al. (2016)^DR^	46	45	Both	Psychotic disorders^1^	DMTS	Negative	Task-irrelevant	Reported
	23	45		Currently psychotic^1^				
	27	47		Healthy				
Schenkel, Passarotti, Sweeney, and Pavuluri (2012)	31	13	Both	Healthy	*n*-back	Negative and positive	Task-relevant	
	23	13		Bipolar disorder I^1^				
	16	15		Bipolar disorder II^1^				
Schweizer and Dalgleish (2011)	33	47	Accuracy	Healthy	C. span	Negative	Task-irrelevant	
	20	46		PTSD^1^				
Schweizer and Dalgleish (2016)	31	23	Accuracy	Healthy	C. span	Negative	Task-irrelevant	
	59	45		Healthy				
	15	46		Healthy				
	12	46		PTSD^1^				
Schweizer et al. (2011)	45	25	Accuracy	Healthy	D. *n-*back	Negative	Task-relevant	
Schweizer et al. (2013)	34	23	Accuracy	Healthy	D. *n-*back	Negative	Task-relevant	Reported
Schweizer et al. (2018)	123	41	Accuracy	Healthy	C. span	Negative	Task-relevant	
	14	51		Healthy				
	21	52		Depression^1^				
	20	37		Healthy				
	27	44		Depression^1^				
	23	51		Remitted depression^1c^				
Segal, Kessler, and Anholt (2015)^DR^	34	23	Accuracy	Healthy	*n-*back	Negative and positive	Task-relevant	
	31	23		High social anxiety^1^				
Shi, Gao, and Zhou (2014)	53	20	Accuracy	High test anxiety^1^	C. span	Negative	Task-irrelevant	
	58	20		Healthy				
Simione et al. (2014)^DR^	22	23	Both	Healthy	DMTS	Negative and positive	Task-relevant	
	20	27	Both	Healthy	DMTS	Negative and positive	Task-relevant	
Simon et al. (2015)^DR^	18	27	Both	Healthy	*n-*back	Negative and positive	Task-irrelevant	Reported*
	18	27		Sleep-deprived^2^				
Stiernströmer, Wolgast, and Johansson (2016)	38	26	Both	Healthy	*n-*back	Negative and positive	Task-relevant	
Tamm, Kreegipuu, Harro, and Cowan (2017)^DR^	507	25	Both	Healthy	*n-*back	Negative and positive	Task-relevant	
Tavares, Logie, and Mitchell (2016)	39	22	Accuracy	Healthy	DMTS	Negative	Task-irrelevant	
	53	21		Healthy				
Tavitian et al. (2014)^DR^	22	16	Accuracy	Depression^1^	*n-*back	Negative and positive	Task-irrelevant	
	21	15		Healthy				
Tempesta, De Gennaro, Presaghi, and Ferrara (2014)^DR^	25	24	Both	Healthy	*n-*back	Negative and positive	Task-relevant	
	25	24		Sleep-deprived^2^				
Terfehr et al. (2011)	27	34	Accuracy	Depression^1^	S. span	Negative	Task-relevant	
	30	34		Depression after hydrocortisone administration^1^				
	29	32		Healthy				
	27	32		Healthy after hydrocortisone administration^2^				
Truong and Yang (2014)	36	20	Both	Healthy	DMTS	Negative and positive	Task-relevant	
	36	73		Healthy				
Uher, Brooks, Bartholdy, Tchanturia, and Campbell (2014)	31	25	Accuracy	Healthy	*n-*back	Negative and positive	Task-relevant	
Vanderhasselt, Brunoni, Loeys, Boggio, and De Raedt (2013)	22	22	Accuracy	Healthy	S. span	Negative	Task-relevant	
	22	22		Healthy after tDCS^2^				
Vermeulen, Niedenthal, Pleyers, Bayot, and Corneille (2014)^DR^	33	19	Both	Healthy	*n-*back	Negative and positive	Task-relevant	
Visu-Petra, Ţincaş, Cheie, and Benga (2010)	30	6	Accuracy	Healthy	S. span	Negative and positive (mixed)	Task-relevant	
	30	6		Healthy				
Wanmaker, Geraerts, and Franken (2015)	49	46	RT^e^	Depression and anxiety^1^	S. span	Negative	Task-relevant	
	49	47		Depression and anxiety^1^				
Weigand et al. (2013)	26	26	Both	Healthy	*n-*back	Negative	Task-relevant	
Weigand et al. (2013)	15	25	Both	Healthy	*n-*back	Negative	Task-relevant	
Wilson, Sayette, Fiez, and Brough (2007)	23	34	Accuracy	Smokers	DMTS	Smoking-related cues		
Xin and Lei (2015)^DR^	33	22	Both	Healthy	*n-*back	Negative and positive	Task-relevant	Reported
Yang, Wang, Jin, and Li (2015)	14	24	Accuracy	Healthy	DMTS	Negative and positive	Task-irrelevant	
Zhang et al. (2013)	20	32	Accuracy	Healthy	DMTS	Negative	Task-irrelevant	Reported
	20	33		PTSD^1^				
*Note*. The effect sizes for the studies represented in Table 1 are summarized in two separate forest plots (Figure S1A and B) in the online supplementary materials. Age = Average age of the study participants; Data = type of data extracted from the study (i.e., accuracy, RT = reaction time, or both); Population = Participant sample included (i.e., healthy, psychopathology, denoted as ^1^; or neurological disorder and altered neurological state group, denoted as ^2^); Task = task design reported in the study; Valence = valence of the affective stimuli (i.e., negative and/or positive); Task-relevance = task-relevance of affective stimuli (i.e., task-irrelevant distractors or task-relevant targets); Imaging = this column states “Reported” for studies that included neuroimaging data on the affective WM task and an asterisk (*) indicates that the study was included in the neuroimaging meta-analysis. DR = data requested from the authors; PTSD = posttraumatic stress disorder; PD = personality disorder; DMTS = delayed-match-to-sample task; S. span = simple span tasks; C. span = complex span tasks; D. *n*-back = dual *n*-back; + means that study included several experiments with different tasks including the *n*-back task, AX-CPT = AX continuous performance task required the continuous updating of cue and probes with distractors presented in between the presentation of cues and distractors.								
^a^ Women with reported child abuse were included in the psychopathology and analogue group due to high levels of depression and anxiety symptoms. ^b^ Women with reported childhood stressors (other than abuse) were included in the healthy group due to nonclinical levels of self-reported depression and anxiety symptoms. ^c^ We included these participants in the psychopathology and analogue group, however, as they were currently asymptomatic we also ran all analyses with them classified as healthy, which did not change the pattern of results. ^d^ Authors had no longer access to the behavioral data. ^e^ In consultation with the authors no accuracy data were included for studies administering the internal shift task as this measure assess accuracy on a block rather than trial basis leading to frequent missing data and accuracy scores that are not directly comparable with other measures of WM (correspondence with Koster is available upon request). ^f^ We included only the neuroimaging data from this study as the authors did not reply to our request for behavioral data before our analysis deadline. ^g^ The study compared WM for face and houses with faces constituting the emotional condition. ^h^ There was no distraction in the delay interval; however, the probe was only an extract of the sample requiring active reconstruction of the original stimulus and was therefore included. ^i^ RT data included in Pallesen, Brattico, Bailey, Korvenoja, and Gjedde (2009).								

**Table 2 tbl2:** Effect Sizes for Each Type of Stimulus Across Task-Relevance (Task-Relevant and Irrelevant) and Valence (Positive and Negative) for WM Accuracy and WM Reaction Time

Stimulus type	*k*	*d̂*	95% CI [LB, UB]	*SEM*	*Q*
Accuracy					
Task-irrelevant distractors					
Positive	22	0.11	−0.10, 0.51	0.16	85.23***
Negative	112	−0.07	−0.22, 0.07	0.07	547.71***
Task-relevant targets					
Positive	95	0.09^†^	−0.00, 0.20	0.05	454.76***
Negative	156	0.11*	0.00, 0.23	0.06	1350.91***
Reaction time					
Task-irrelevant distractors					
Positive	22	0.11*	0.01, 0.21	0.05	39.06***
Negative	81	0.05	−0.03, 0.13	0.04	123.26***
Task-relevant targets					
Positive	86	−0.04	−0.14, 0.06	0.05	172.03***
Negative	116	0.11**	0.03, 0.18	0.04	175.41***
*Note*. The table reports effect sizes on WM accuracy and reaction time of the comparison between affective stimuli of a certain task-relevance and valence and neutral stimuli of the same task-relevance.					
^†^ ≤ .10. * *p* < .05. ** *p* < .01. *** *p* ≤ .001.					

**Table 3 tbl3:** Brain Regions Consistently Engaged During Affective Compared With Neutral Stimulus Conditions in WM Tasks

Region	L/R	Cluster size (voxels)	Subcluster size (voxels)	Maximum	x/y/z
Negative > Neutral Stimuli					
vlPFC/OFC	L	228		.31	−39/33/−6
			71		−39/36/3
Amygdala	L	338		.35	−27/−3/−18
			103		−24/−12/−18
			80		−18/0/−18
			46		−21/−6/−6
Temporal lobe (including amygdalo-hippocampal complex)	R	419		.50	21/−6/−18
			48		36/0/−24
			51		39/−3/−15
Temporo-occipital lobe (including fusiform gyrus)	L	648		.29	−39/−57/−12
			192		−42/−78/0
			93		−45/−75/12
			110		−36/−51/−21
			36		−51/−63/6
			34		−48/−51/3
Temporo-occipital lobe (including fusiform gyrus)	R	512		.33	42/−54/−18
			110		39/−75/−12
			87		42/−45/−12
			70		42/−72/0
			45		48/−69/−12
			33		42/−54/−6
			60		54/−69/6
Neutral > Negative stimuli					
dlPFC	R	505		.49	36/42/30
			180		36/33/33
			34		27/51/12
			51		33/54/18
			44		33/39/42
			21		27/30/51
*Note*. Table 3 reports brain regions that were significantly more frequently activated in response to one condition compared with another. Peak activations for each (sub)cluster are reported as well as the maximum statistic, which reflects the analysis of the distribution of maximum values corrected for multiple comparisons at a FWER of .05 (Salimi-Khorshidi, Smith, Keltner, Wager, and Nichols, 2009; Wager, Lindquist, and Kaplan, 2007). dl = dorsolateral; vl = ventrolateral; PFC = prefrontal cortex; OFC = orbitofrontal cortex; L = left; R = right; Maximum = maximum of the *z*-field. The negative > neutral comparison was based on 211 coordinates from 24 contrasts; the neutral > negative comparison was based on 144 coordinates from 20 contrasts.					

## Footnotes

1 There is a multitude of competing theories of WM, generating vibrant debate around definitions and underlying mechanisms (cf. Miyake & Shah, 1999) which we acknowledge. An indebt discussion of these debates, however, is beyond the scope of this review.

2 It should be noted that the arousal-biased competition model (Mather & Sutherland, 2011), qualifies as an exception to this enhancement. This theory suggests that WM performance for multiple task-relevant items will be impaired for affective compared with neutral targets. The rationale is that, while maintaining affective stimuli in WM, the prioritized attentional processing afforded to the active affective item increases the costs for the competing stimuli more than for less arousing neutral items.

3 However, see Brosch, Sander, Pourtois, and Scherer (2008) for highly similar patterns of attentional capture for both negative and positive pictures.

4 Studies that simply presented a delay interval without distractors or a secondary task were not included as we consider them to be better conceptualized as perceptual, short-term or long-term memory tasks depending on the delay interval.

5 There is no published review protocol for the two meta-analyses reported in this article.

6 The asterisks in the search term combinations denote so-called wild cards in the database search. That is, every permutation of the term is entered and searched within the database (e.g., emoti* performs searches including emotive, emotion, emotional, etc., . . .).

7 Some studies included neuropsychological populations. Data from these samples were excluded from the moderation analyses, as they were not part of the investigation of interest. The healthy control samples from those studies, however, were included in the moderation analyses.

8 It was not possible to look at the comparisons between neutral and affective target stimuli, because there were insufficient studies reporting the contrast neutral > affective.

9 There were insufficient contrasts including neutral memoranda to look at the effects of affective versus neutral stimuli across task-relevant and task-irrelevant material.

10 It should be noted here that theorists have argued that there is no affect-free cognition (Barrett, 2006, 2009; Lindquist, 2013) and seemingly well-validated “neutral” stimuli elicit significant amounts of ambivalence, which is related to arousal (Schneider, Veenstra, van Harreveld, Schwarz, & Koole, 2016).
