# Supplementary material for: The Impact of Affective Information on Working Memory: A Pair of Meta-Analytic Reviews of Behavioral and Neuroimaging Evidence
Source: Psychol Bull. 2019 Apr 25;145(6):566–609. doi: 10.1037/bul0000193 (PMC6526745; doi:10.1037/bul0000193)
Supplement: Supplementary file 1 [file bul0000193_Supplementary-Materials.docx]

**Supplementary Materials**

The impact of affective information on working memory: A pair of meta-analytic reviews of behavioral and neuroimaging evidence

## SM1: Note on the effects of affective information on attention and long-term memory

Affective stimuli have shown robust effects on a range of cognitive functions including attention (Vuilleumier, 2005; Vuilleumier & Huang, 2009) and long-term memory (LaBar & Cabeza, 2006). Compared to neutral stimuli, affective stimuli are preferentially processed in attention with dissociable effects on performance on attentional measures as a function of the affective stimulus’ task-relevance. Given the competition of perceptual information for limited attentional processing resources (Desimone & Duncan, 1995; J. Duncan, 2006) task-relevant affective material enhances performance on measures of attention, whereas task-irrelevant affective distractors reduce performance relative to neutral stimuli (Carretié, 2014; Vuilleumier, 2005). In the long-term memory literature there is unequivocal evidence for an affective memory enhancement, which is dependent on increased (relative to neutral) activity of the amygdala and its interactions with other brain regions in particular the hippocampal complex (Buchanan & Adolphs, 2002; Paz & Pare, 2013; Phelps, 2006).

## SM2: Preferred Reporting Items for Systematic Reviews and Meta-Analyses (PRISMA) Checklist

### Table S1. PRISMA Checklist

| **Section/topic** | **#** | **Checklist item** | **Reported on page #** |
| --- | --- | --- | --- |
| **TITLE** | | |  |
| Title | 1 | Identify the report as a systematic review, meta-analysis, or both. | 1 |
| **ABSTRACT** | | |  |
| Structured summary | 2 | Provide a structured summary including, as applicable: background; objectives; data sources; study eligibility criteria, participants, and interventions; study appraisal and synthesis methods; results; limitations; conclusions and implications of key findings; systematic review registration number. | 2 |
| **INTRODUCTION** | | |  |
| Rationale | 3 | Describe the rationale for the review in the context of what is already known. | 3-17 |
| Objectives | 4 | Provide an explicit statement of questions being addressed with reference to participants, interventions, comparisons, outcomes, and study design (PICOS). | 16 |
| **METHODS** | | |  |
| Protocol and registration | 5 | Indicate if a review protocol exists, if and where it can be accessed (e.g., Web address), and, if available, provide registration information including registration number. | 17 |
| Eligibility criteria | 6 | Specify study characteristics (e.g., PICOS, length of follow-up) and report characteristics (e.g., years considered, language, publication status) used as criteria for eligibility, giving rationale. | 17-18 |
| Information sources | 7 | Describe all information sources (e.g., databases with dates of coverage, contact with study authors to identify additional studies) in the search and date last searched. | 17 |
| Search | 8 | Present full electronic search strategy for at least one database, including any limits used, such that it could be repeated. | SM 3. pp. 6-14 |
| Study selection | 9 | State the process for selecting studies (i.e., screening, eligibility, included in systematic review, and, if applicable, included in the meta-analysis). | Figure 2 (behavioral) and Figure S2 (neuroimaging) |
| Data collection process | 10 | Describe method of data extraction from reports (e.g., piloted forms, independently, in duplicate) and any processes for obtaining and confirming data from investigators. | 17-18 |
| Data items | 11 | List and define all variables for which data were sought (e.g., PICOS, funding sources) and any assumptions and simplifications made. | 18-20 |
| Risk of bias in individual studies | 12 | Describe methods used for assessing risk of bias of individual studies (including specification of whether this was done at the study or outcome level), and how this information is to be used in any data synthesis. | 18-19 |
| Summary measures | 13 | State the principal summary measures (e.g., risk ratio, difference in means). | 18-20 |
| Synthesis of results | 14 | Describe the methods of handling data and combining results of studies, if done, including measures of consistency (e.g., I^2^) for each meta-analysis. | 18-20 |

## SM3: Electronic search strategy

In accordance with the PRISMA guidelines we report an example of the electronic search strategies for our first search term combination searched in PubMed, the PsycInfo equivalent was used for the search in that database.

### Search combination I

(emotion[All Fields] OR emotion'[All Fields] OR emotion's[All Fields] OR emotion1[All Fields] OR emotiona[All Fields] OR emotionaal[All Fields] OR emotionaalinen[All Fields] OR emotionaaliset[All Fields] OR emotionaalisten[All Fields] OR emotionability[All Fields] OR emotionai[All Fields] OR emotional[All Fields] OR emotional'[All Fields] OR emotional''[All Fields] OR emotional'nogo[All Fields] OR emotional'nom[All Fields] OR emotional'nomu[All Fields] OR emotional'nykh[All Fields] OR emotionalbrain[All Fields] OR emotionale[All Fields] OR emotionalem[All Fields] OR emotionalen[All Fields] OR emotionaleninvolviertheit[All Fields] OR emotionaler[All Fields] OR emotionales[All Fields] OR emotionalinstability[All Fields] OR emotionalised[All Fields] OR emotionalisera[All Fields] OR emotionalisieren[All Fields] OR emotionalisieren'[All Fields] OR emotionalisierung[All Fields] OR emotionalism[All Fields] OR emotionalist[All Fields] OR emotionalistic[All Fields] OR emotionalitaet[All Fields] OR emotionalitat[All Fields] OR emotionalitatsinventar[All Fields] OR emotionaliteit[All Fields] OR emotionalities[All Fields] OR emotionality[All Fields] OR emotionality'[All Fields] OR emotionalization[All Fields] OR emotionalize[All Fields] OR emotionalized[All Fields] OR emotionalizes[All Fields] OR emotionalizing[All Fields] OR emotionallly[All Fields] OR emotionallogiccentre[All Fields] OR emotionally[All Fields] OR emotionally'[All Fields] OR emotionalneglect[All Fields] OR emotionalpersonality[All Fields] OR emotionals[All Fields] OR emotionalstress[All Fields] OR emotioncognition[All Fields] OR emotiondetect[All Fields] OR emotiondiary[All Fields] OR emotione[All Fields] OR emotioned[All Fields] OR emotioneel[All Fields] OR emotionele[All Fields] OR emotionell[All Fields] OR emotionella[All Fields] OR emotionelle[All Fields] OR emotionellem[All Fields] OR emotionellen[All Fields] OR emotioneller[All Fields] OR emotionelles[All Fields] OR emotionellt[All Fields] OR emotionen[All Fields] OR emotioner[All Fields] OR emotionful[All Fields] OR emotionful'[All Fields] OR emotiongenic[All Fields] OR emotioning[All Fields] OR emotionis[All Fields] OR emotionl[All Fields] OR emotionlab[All Fields] OR emotionladen[All Fields] OR emotionless[All Fields] OR emotionlike[All Fields] OR emotionmemory[All Fields] OR emotionnal[All Fields] OR emotionnalite[All Fields] OR emotionnel[All Fields] OR emotionnelle[All Fields] OR emotionnelles[All Fields] OR emotionnels[All Fields] OR emotiono[All Fields] OR emotionogenic[All Fields] OR emotionogennykh[All Fields] OR emotionol[All Fields] OR emotionology[All Fields] OR emotionregulation[All Fields] OR emotionregulationlab[All Fields] OR emotions[All Fields] OR emotions'[All Fields] OR emotions,[All Fields] OR emotionsausdruck[All Fields] OR emotionsbegriffen[All Fields] OR emotionsby[All Fields] OR emotionsdysregulation[All Fields] OR emotionserkennung[All Fields] OR emotionserleben[All Fields] OR emotionserlebens[All Fields] OR emotionsfokussierte[All Fields] OR emotionsfokussierter[All Fields] OR emotionsforschung[All Fields] OR emotionsforskningen[All Fields] OR emotionsgehalt[All Fields] OR emotionsinduktion[All Fields] OR emotionsliste[All Fields] OR emotionspsychologie[All Fields] OR emotionspsychose[All Fields] OR emotionspsychosen[All Fields] OR emotionsregulation[All Fields] OR emotionsregulationsstrategien[All Fields] OR emotionsregulative[All Fields] OR emotionsregulierung[All Fields] OR emotionssozialisation[All Fields] OR emotionsstorungen[All Fields] OR emotionsstress[All Fields] OR emotionssuppression[All Fields] OR emotionstheorie[All Fields] OR emotionstheorien[All Fields] OR emotionsverarbeitung[All Fields] OR emotionsverstandnis[All Fields] OR emotionswissens[All Fields] OR emotiontional[All Fields] OR emotiontropic[All Fields] OR emotionxgroup[All Fields] OR emotionxorder[All Fields]) AND (cogniti[All Fields] OR cognitia[All Fields] OR cognitial[All Fields] OR cognitian[All Fields] OR cognitica[All Fields] OR cognitice[All Fields] OR cognitician[All Fields] OR cognitie[All Fields] OR cognitief[All Fields] OR cognitieffunctioneren[All Fields] OR cognities[All Fields] OR cognitieve[All Fields] OR cognitif[All Fields] OR cognitifs[All Fields] OR cognitil[All Fields] OR cognitin[All Fields] OR cognitin's[All Fields] OR cognitins[All Fields] OR cognitio[All Fields] OR cognitio'[All Fields] OR cognitioin[All Fields] OR cognition[All Fields] OR cognition'[All Fields] OR cognition's[All Fields] OR cognition,[All Fields] OR cognition2[All Fields] OR cognition93[All Fields] OR cognitional[All Fields] OR cognitionand[All Fields] OR cognitionbehavior[All Fields] OR cognitioncleveland[All Fields] OR cognitiondisability[All Fields] OR cognitione[All Fields] OR cognitionemotion[All Fields] OR cognitionincarnation[All Fields] OR cognitionis[All Fields] OR cognitionleiden[All Fields] OR cognitionleidenthe[All Fields] OR cognitionm[All Fields] OR cognitionmarseille[All Fields] OR cognitionmaster[All Fields] OR cognitionmetrics[All Fields] OR cognitionnetherlands[All Fields] OR cognitionrelated[All Fields] OR cognitionresponse[All Fields] OR cognitions[All Fields] OR cognitions'[All Fields] OR cognitionstudy[All Fields] OR cognitiontoulouse[All Fields] OR cognitionumr[All Fields] OR cognitiori[All Fields] OR cognitiove[All Fields] OR cognitique[All Fields] OR cognitition[All Fields] OR cognititve[All Fields] OR cognitiu[All Fields] OR cognitius[All Fields] OR cognitiv[All Fields] OR cognitiva[All Fields] OR cognitivas[All Fields] OR cognitive[All Fields] OR cognitive'[All Fields] OR cognitiveability[All Fields] OR cognitiveatlas[All Fields] OR cognitivebehavior[All Fields] OR cognitivebehavioral[All Fields] OR cognitivebehavioural[All Fields] OR cognitivebehaviourtherapy[All Fields] OR cognitiveconsilience[All Fields] OR cognitivedrugresearch[All Fields] OR cognitivedysfunction[All Fields] OR cognitiveflexibility[All Fields] OR cognitivefunctioning[All Fields] OR cognitivegroup[All Fields] OR cognitiveion[All Fields] OR cognitiveiy[All Fields] OR cognitiveliberty[All Fields] OR cognitively[All Fields] OR cognitively'[All Fields] OR cognitivem[All Fields] OR cognitivement[All Fields] OR cognitiveness[All Fields] OR cognitiveneuroscience[All Fields] OR cognitiveperformance[All Fields] OR cognitiver[All Fields] OR cognitives[All Fields] OR cognitives'[All Fields] OR cognitivescience[All Fields] OR cognitivesocial[All Fields] OR cognitivestrategies[All Fields] OR cognitivesystemsdesign[All Fields] OR cognitivetesting[All Fields] OR cognitiveview[All Fields] OR cognitivi[All Fields] OR cognitivie[All Fields] OR cognitivism[All Fields] OR cognitivismo[All Fields] OR cognitivist[All Fields] OR cognitivist'[All Fields] OR cognitivist's[All Fields] OR cognitivista[All Fields] OR cognitiviste[All Fields] OR cognitivistic[All Fields] OR cognitivists[All Fields] OR cognitivists'[All Fields] OR cognitivity[All Fields] OR cognitivnogo[All Fields] OR cognitivo[All Fields] OR cognitivobehavioral[All Fields] OR cognitivocomportementale[All Fields] OR cognitivoemotional[All Fields] OR cognitivoform[All Fields] OR cognitivos[All Fields] OR cognitivr[All Fields] OR cognitivre[All Fields] OR cognitivterapi[All Fields]) AND (function[All Fields] OR function'[All Fields] OR function''[All Fields] OR function'alis[All Fields] OR function'in[All Fields] OR function's[All Fields] OR function'were[All Fields] OR function,[All Fields] OR function1[All Fields] OR function2[All Fields] OR function26[All Fields] OR function28[All Fields] OR function2a[All Fields] OR function2gene[All Fields] OR function490[All Fields] OR function647[All Fields] OR functiona[All Fields] OR functionaal[All Fields] OR functionabilities[All Fields] OR functionability[All Fields] OR functionable[All Fields] OR functionaction[All Fields] OR functionae[All Fields] OR functionai[All Fields] OR functionaing[All Fields] OR functionaires[All Fields] OR functionais[All Fields] OR functionaities[All Fields] OR functionakimageanalysis[All Fields] OR functional[All Fields] OR functional'[All Fields] OR functional''[All Fields] OR functional'noe[All Fields] OR functional'proximal[All Fields] OR functional's[All Fields] OR functionala[All Fields] OR functionalability[All Fields] OR functionalactivity[All Fields] OR functionalal[All Fields] OR functionalanalysis[All Fields] OR functionalanalytical[All Fields] OR functionaland[All Fields] OR functionalbeta[All Fields] OR functionalbiological[All Fields] OR functionalchanges[All Fields] OR functionalclass[All Fields] OR functionalconstipation[All Fields] OR functionaldiagnostic[All Fields] OR functionaldog1[All Fields] OR functionale[All Fields] OR functionalefficiency[All Fields] OR functionalenzyme[All Fields] OR functionales[All Fields] OR functionalflow[All Fields] OR functionalgastroenterology[All Fields] OR functionalgenomics[All Fields] OR functionalghd7allele[All Fields] OR functionalglycomics[All Fields] OR functionalgrazers[All Fields] OR functionalgroup[All Fields] OR functionalgroupentity[All Fields] OR functionalgroups[All Fields] OR functionali[All Fields] OR functionaliation[All Fields] OR functionalied[All Fields] OR functionalil6[All Fields] OR functionalimaging[All Fields] OR functionalin[All Fields] OR functionalinsts[All Fields] OR functionalis[All Fields] OR functionalisable[All Fields] OR functionalisation[All Fields] OR functionalisations[All Fields] OR functionalisative[All Fields] OR functionalise[All Fields] OR functionalised[All Fields] OR functionalised'[All Fields] OR functionalises[All Fields] OR functionalising[All Fields] OR functionalism[All Fields] OR functionalismo[All Fields] OR functionalisms[All Fields] OR functionalist[All Fields] OR functionalist'[All Fields] OR functionalist's[All Fields] OR functionalistic[All Fields] OR functionalistion[All Fields] OR functionalists[All Fields] OR functionalitate[All Fields] OR functionalitatea[All Fields] OR functionalitatii[All Fields] OR functionalitation[All Fields] OR functionalites[All Fields] OR functionalities[All Fields] OR functionalities'[All Fields] OR functionalitites[All Fields] OR functionality[All Fields] OR functionality'[All Fields] OR functionality's[All Fields] OR functionalizability[All Fields] OR functionalizable[All Fields] OR functionalizaed[All Fields] OR functionalizarea[All Fields] OR functionalizated[All Fields] OR functionalizating[All Fields] OR functionalization[All Fields] OR functionalization'[All Fields] OR functionalization's[All Fields] OR functionalization5[All Fields] OR functionalizationed[All Fields] OR functionalizationof[All Fields] OR functionalizations[All Fields] OR functionalizatlon[All Fields] OR functionalizaton[All Fields] OR functionalizd[All Fields] OR functionalize[All Fields] OR functionalizeable[All Fields] OR functionalized[All Fields] OR functionalizers[All Fields] OR functionalizes[All Fields] OR functionalizes'[All Fields] OR functionaliziation[All Fields] OR functionalizied[All Fields] OR functionalizing[All Fields] OR functionalizion[All Fields] OR functionaliztion[All Fields] OR functionallaczgene[All Fields] OR functionallimitations[All Fields] OR functionallized[All Fields] OR functionallly[All Fields] OR functionally[All Fields] OR functionally'[All Fields] OR functionallydependent[All Fields] OR functionallyidentical[All Fields] OR functionallywise[All Fields] OR functionalmedicine[All Fields] OR functionalmifpromoter[All Fields] OR functionalmitral[All Fields] OR functionalmolecular[All Fields] OR functionalmorphology[All Fields] OR functionalmri[All Fields] OR functionalnature[All Fields] OR functionalnet[All Fields] OR functionalnoda[All Fields] OR functionaloutcomes[All Fields] OR functionalpolarities[All Fields] OR functionalproteins[All Fields] OR functionalroles[All Fields] OR functionals[All Fields] OR functionals'[All Fields] OR functionalsimilarity[All Fields] OR functionalskill[All Fields] OR functionalskills[All Fields] OR functionalsolutions[All Fields] OR functionalstructure[All Fields] OR functionalstudies[All Fields] OR functionalties[All Fields] OR functionalvoice[All Fields] OR functionalvs[All Fields] OR functionaly[All Fields] OR functionalysed[All Fields] OR functionalyzed[All Fields] OR functionalzation[All Fields] OR functionalziation[All Fields] OR functionalzied[All Fields] OR functionamento[All Fields] OR functionamiento[All Fields] OR functionanalyzer[All Fields] OR functionand[All Fields] OR functionante[All Fields] OR functionantes[All Fields] OR functionare[All Fields] OR functionarea[All Fields] OR functionaresse[All Fields] OR functionaries[All Fields] OR functionaries'[All Fields] OR functionarii[All Fields] OR functionarios[All Fields] OR functionaris[All Fields] OR functionarissen[All Fields] OR functionarization[All Fields] OR functionary[All Fields] OR functionary'[All Fields] OR functionas[All Fields] OR functionassociated[All Fields] OR functionate[All Fields] OR functionates[All Fields] OR functionating[All Fields] OR functionation[All Fields] OR functionator[All Fields] OR functionbody[All Fields] OR functionbrix[All Fields] OR functionc[All Fields] OR functioncdots[All Fields] OR functiond[All Fields] OR functiondagger[All Fields] OR functionde[All Fields] OR functione[All Fields] OR functioneal[All Fields] OR functioned[All Fields] OR functioneel[All Fields] OR functioneelanatomisch[All Fields] OR functioneert[All Fields] OR functionel[All Fields] OR functionele[All Fields] OR functionelisa[All Fields] OR functionelle[All Fields] OR functionellen[All Fields] OR functioneller[All Fields] OR functionelles[All Fields] OR functionellipsis[All Fields] OR functionellipsis'[All Fields] OR functionem[All Fields] OR functionen[All Fields] OR functioner[All Fields] OR functioneren[All Fields] OR functionerend[All Fields] OR functionerende[All Fields] OR functionerings[All Fields] OR functiones[All Fields] OR functionfor[All Fields] OR functionfunctions[All Fields] OR functiong[All Fields] OR functionh[All Fields] OR functionht1[All Fields] OR functionht1mutants[All Fields] OR functioni[All Fields] OR functionialized[All Fields] OR functionialy[All Fields] OR functionig[All Fields] OR functionilized[All Fields] OR functionimg[All Fields] OR functionin[All Fields] OR functioning[All Fields] OR functioning'[All Fields] OR functioning's[All Fields] OR functioninga[All Fields] OR functioningand[All Fields] OR functioningfeatures[All Fields] OR functioningin[All Fields] OR functionings[All Fields] OR functionings'[All Fields] OR functioningt[All Fields] OR functioningthe[All Fields] OR functionioning[All Fields] OR functionisable[All Fields] OR functionization[All Fields] OR functionized[All Fields] OR functionl[All Fields] OR functionla[All Fields] OR functionlaized[All Fields] OR functionless[All Fields] OR functionless'[All Fields] OR functionlessness[All Fields] OR functionlike[All Fields] OR functionlization[All Fields] OR functionlize[All Fields] OR functionlized[All Fields] OR functionlmnagene[All Fields] OR functionly[All Fields] OR functionment[All Fields] OR functionmuscle[All Fields] OR functionn[All Fields] OR functionnal[All Fields] OR functionnale[All Fields] OR functionnalization[All Fields] OR functionnalized[All Fields] OR functionnally[All Fields] OR functionnaly[All Fields] OR functionned[All Fields] OR functionnel[All Fields] OR functionnele[All Fields] OR functionnelle[All Fields] OR functionnelles[All Fields] OR functionnels[All Fields] OR functionnement[All Fields] OR functionnes[All Fields] OR functionning[All Fields] OR functionnlrp3allele[All Fields] OR functiono[All Fields] OR functionof[All Fields] OR functionograph[All Fields] OR functionography[All Fields] OR functionome[All Fields] OR functionome'[All Fields] OR functionomes[All Fields] OR functionometric[All Fields] OR functionomic[All Fields] OR functionomics[All Fields] OR functionomics'[All Fields] OR functionpattern[All Fields] OR functionpc[All Fields] OR functionpiso[All Fields] OR functionplasma[All Fields] OR functionplaster[All Fields] OR functionpost[All Fields] OR functionprediction[All Fields] OR functionpsi[All Fields] OR functionrelated[All Fields] OR functionrelationship[All Fields] OR functionroi[All Fields] OR functions[All Fields] OR functions'[All Fields] OR functions's[All Fields] OR functionsal[All Fields] OR functionsalk[All Fields] OR functionsand[All Fields] OR functionsaving[All Fields] OR functionse[All Fields] OR functionsf[All Fields] OR functionship[All Fields] OR functionsin[All Fields] OR functionsit[All Fields] OR functionskane[All Fields] OR functionsleuphana[All Fields] OR functionsmduring[All Fields] OR functionsmeasured[All Fields] OR functionsrelated[All Fields] OR functionsreproduce[All Fields] OR functionsstorungen[All Fields] OR functionstate[All Fields] OR functionsuccess[All Fields] OR functionsurg[All Fields] OR functionsviaepigenetic[All Fields] OR functionswechsel[All Fields] OR functionswechsels[All Fields] OR functionswere[All Fields] OR functionswithin[All Fields] OR functiont[All Fields] OR functiontesting[All Fields] OR functionthat[All Fields] OR functionthere[All Fields] OR functiontional[All Fields] OR functiontrpc[All Fields] OR functionum[All Fields] OR functionvariants[All Fields] OR functionviaactivation[All Fields] OR functionwere[All Fields] OR functionwhiles[All Fields] OR functionwise[All Fields] OR functionx[All Fields] OR functiony[All Fields] OR functionzetaunder[All Fields]) AND (("1900/01/01"[PDAT] : "2017/02/28"[PDAT]) AND "humans"[MeSH Terms] AND English[lang]).

## SM4: Reasons for exclusion in the behavioral meta-analysis

Supplementary data file available in online supplementary materials.

## SM5: Supplementary methods and results for sensitivity analyses

It should be noted here that regression tests and funnel plots are relatively crude estimates of publication biases. An important advance has been made by introducing sensitivity analyses to the field of data syntheses (Coburn & Vevea, 2015b; Vevea & Woods, 2005b). These sensitivity analyses, however, are problematic for the current investigations because the model estimates included in the computation of sensitivity analyses assume the input of a singular, “homogenous” data type such as behavioral data, physiological data, or genetic data. The integration of data across both behavioral and neural levels of analysis in the current study creates a problem for sensitivity analyses because imaging data will be published in the absence of significant behavioral findings, which renders the standard weighting of the sensitivity analysis less sensitive. Additionally, the current study also included comparisons across groups with different neuropsychological profiles (incl., altered sleep states, neurological disorders) and mental health status (individuals suffering from psychopathology compared to healthy individuals). Such studies will be published on the basis of group differences and not on the overall comparison of the effects of affective relative to neutral information. These difficulties preclude a valid estimation of sensitivity analysis weightings, and so we opted against the use of sensitivity analyses here.

## Figure S1A: Forest plot WM accuracy


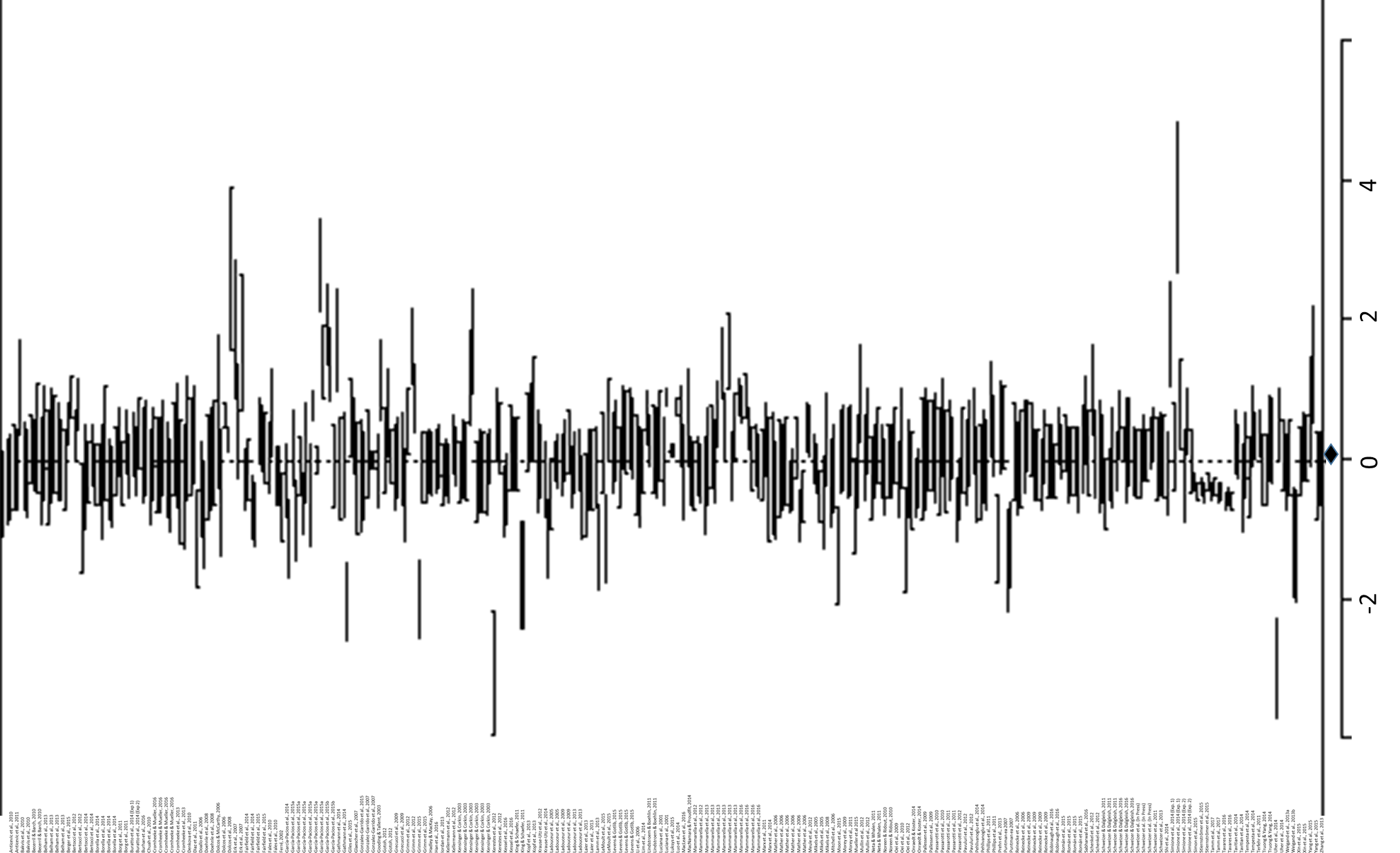


## Figure S1B: Forest plot WM reaction time


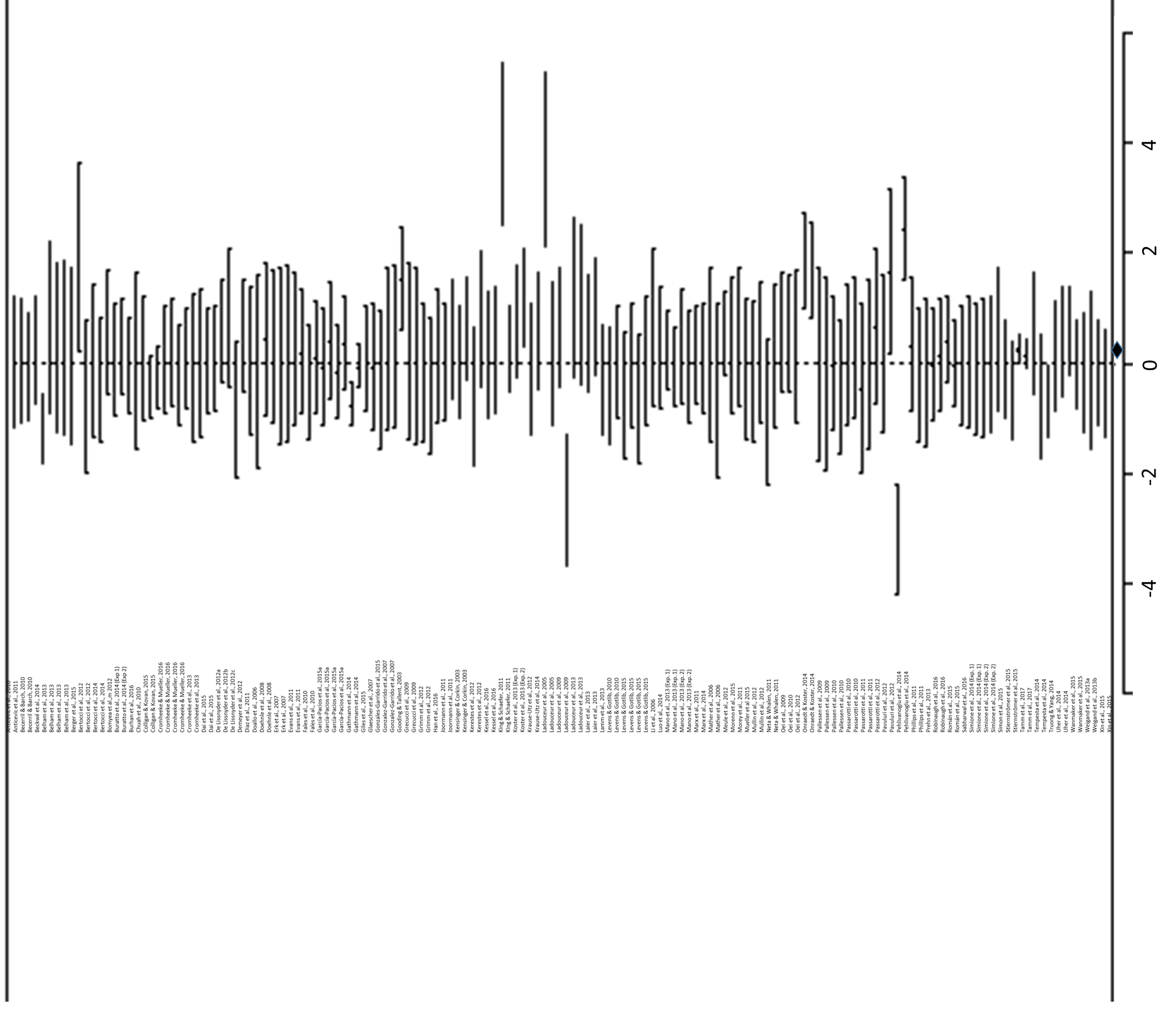


*Figure S1.* The panels represent the effect sizes for each study where effect sizes across valence (negative, positive) were pooled for studies including multiple emotions per valence (e.g., sad, angry and fearful for negative) for WM accuracy (S1A) and reaction time (S1B). The black diamond represents the overall pooled effect size.

## SM6. Exploratory analyses of the effect of emotion type and valence

***Emotion type.*** There was no significant effect of emotion-type on WM accuracy, *k* = 88, *Q_M_* (3) = 2.83, *p* = .419. In contrast, emotion-type had a significant moderating effect on WM reaction time, *k* = 73, *Q_M_* (3) = 16.54, *p* ≤ .001. This effect was due to WM reaction time being faster in tasks including happiness-related stimuli compared to neutral stimuli, *k* = 30, $\hat{d}$_Happy_ = −0.19. CI 95% [‒0.38, 0.00], *SEM* = 0.10, *p* = .050. In contrast, anger, *k* = 13, $\hat{d}$_Anger_ = 0.16, CI 95% [‒0.20, 0.51], *SEM* = 0.18, *p* = .39; fear, *k* = 18, $\hat{d}$_Fear_ = 0.05, CI 95% [‒0.09, 0.18], *SEM* = 0.07, *p* = .503; and sadness-related stimuli, *k* = 11 $\hat{d}$_Sadness_ = 0.11, CI 95% [‒0.04, 0.26], *SEM* = 0.08, *p* = .141, slowed reaction times relative to neutral stimuli.

***Load.*** Results showed no main effect of load on either WM accuracy or reaction time, *p*’s > .648. However, for WM accuracy (not reaction time, *p* = .701) there was a significant interaction between load and task-relevance, *k* = 213, $\hat{d}$ = ‒0.20, CI 95% [‒0.32, ‒0.08], *SEM* = 0.06, *p* = .001, Q_M_ (3) = 19.11, *p* < .001. Breaking down this interaction, we investigated separately the effects of load for task-relevant stimuli, *k* = 132, $\hat{d}$ = 0.07, CI 95% [‒0.03, 0.17], *SEM* = 0.05, *p* = .175, *Q_M_* (1) = 1.83, *p =* .175 and task-irrelevant stimuli, *k* = 81, $\hat{d}$ = ‒0.02, CI 95% [‒0.10, 0.05], *SEM* = 0.04, p = .597, *Q_M_* (1) = 0.28, *p* = .597. While the moderator analyses were not significant, the impact of affective information (relative to neutral) on WM accuracy did show a significant association with load for task-relevant information, *r* (130) = .24, CI 95% [.07, .39], *p* = .006, but not for task-irrelevant distractors, *r* (79) = .07, CI 95% [‒.15, .28], *p* = .533. There were insufficient effect sizes including stimuli of positive valence to investigate the potential interacting effect of task-load and task valence.

## Table S2. Main effects of valence and task-relevance for WM accuracy

|  | *k* | $\hat{d}$ | CI95%[LB,UB] | *SEM* | *Q* |
| --- | --- | --- | --- | --- | --- |
| Valence main effect |  |  |  |  |  |
| Positive | 117 | 0.12* | .02, 0.21 | 0.05 | 549.30*** |
| Negative | 268 | 0.04 | –0.05, 0.13 | 0.05 | 547.71*** |
| Task-relevance main effect |  |  |  |  |  |
| Task-relevant targets | 257 | 0.08 | ‒0.03, 0.18 | 0.05 | 1966.49*** |
| Task-irrelevant distractors | 134 | 0.08 | ‒0.18, 0.10 | 0.06 | 642.97*** |

*Table S2.* *** < .0001, * < .05

## SM7: Supplementary results reporting the effect of valence in task-relevant and task-irrelevant stimuli separately

To explore these effect of task-relevance and valence on WM accuracy and reaction time further we looked at the effect of valence separately in both distractors and targets (Table 2).

*Accuracy.* WM accuracy was subject to a moderating effect of valence for both task-irrelevant affective distractors, *k* = 134, $\hat{d}$ = –0.32, CI 95% [–0.50, –0.13], *SEM* = 0.09, *p* = .0007, *Q_M_* (1) = 11.41, *p* = .006 and task-relevant affective targets, *k* = 248, $\hat{d}$ = 0.18, CI 95% [0.13, 0.22], *SEM* = 0.02, *p* < .0001, *Q_M_* (1) = 53.58, *p* < .0001. In Table 2 we break down these effects further into the univariate comparisons, which showed improved WM accuracy for targets, especially negative targets. However, the effects were small and significant variance was accounted for by study-specific variations.

*Reaction time.*  For reaction time there was again a moderating effect of valence for both task-irrelevant affective distractors, *k* = 134, $\hat{d}$ = –0.10, CI 95% [–0.23, –0.03], *SEM* = 0.06, *p* = .122, *Q_M_* (1) = 2.39, *p* = .122. Task-relevant affective targets, *k* = 202, $\hat{d}$ = 0.06, CI 95% [0.01, 0.12], *SEM* = 0.03, *p* < .040, *Q_M_* (1) = 53.58, *p* < .0001, however, showed a significant effect of valence. In Table 2 we break down this effect further into the univariate comparisons, which showed slowed WM accuracy for negative targets.

**Discussion of these effects.** In an attempt to account for some of study-specific heterogeneity in the findings we ran an exploratory analysis of load, which showed an interactive effect of load and task-relevance on WM accuracy. Task-relevant material, with affective memoranda at low levels (2-4 items) of WM load reducing accuracy but improving performance at high levels (5-6 items) of WM load. Again the absence of support for an effect of load in studies with task-irrelevant affective distractors is surprising given that the load theory of selective attention and cognitive control (Lavie, Hirst, de Fockert, & Viding, 2004), would predict affective distractors at high levels of cognitive load to have the greatest impact. Together these findings suggest that there are negligible effects of affective material on WM accuracy with a significant proportion of the variance being attributable to study-specific factors in healthy individuals. This is arguably primarily due to the lack of affective significance of the affective stimuli typically used in experimental research.

## Table S3. Effects of valence and task-relevance in individuals with mental health problems

|  | *k* | $\hat{d}$ | CI 95% [LB, UB] | *SEM* | *Q* |
| --- | --- | --- | --- | --- | --- |
| **Valence** |  |  |  |  |  |
| Positive | 26 | –0.25 | –0.57, 0.07 | 0.16 | 293.14^***^ |
| Negative | 88 | –0.20^*^ | –0.41, –0.00 | 0.10 | 479.89^***^ |
| **Task-relevance** |  |  |  |  |  |
| Task-relevant | 55 | –0.05 | –0.47, 0.36 | 0.21 | 571.37^***^ |
| Task-irrelevant | 59 | –0.24^†^ | –0.48, 0.00 | 0.12 | 217.41^***^ |

## Table S4. Effect of age across valence

|  | *k* | $\hat{d}$ | CI 95% [LB, UB] | *SEM* | *Q* |
| --- | --- | --- | --- | --- | --- |
| **Accuracy** |  |  |  |  |  |
| Positive | 106 | 0.01^†^ | –0.00, 0.01 | 0.00 | 3.43^†^ |
| Negative | 247 | 0.01^*^ | 0.00, 0.01 | 0.00 | 5.93^*^ |
| **Reaction time** |  |  |  |  |  |
| Positive | 109 | –0.01^**^ | –0.02, –0.00 | 0.00 | 9.15^**^ |
| Negative | 190 | 0.00 | –0.00, 0.01 | 0.00 | 1.85 |

## Figure S2: PRISMA diagram for the neuroimaging meta-analysis


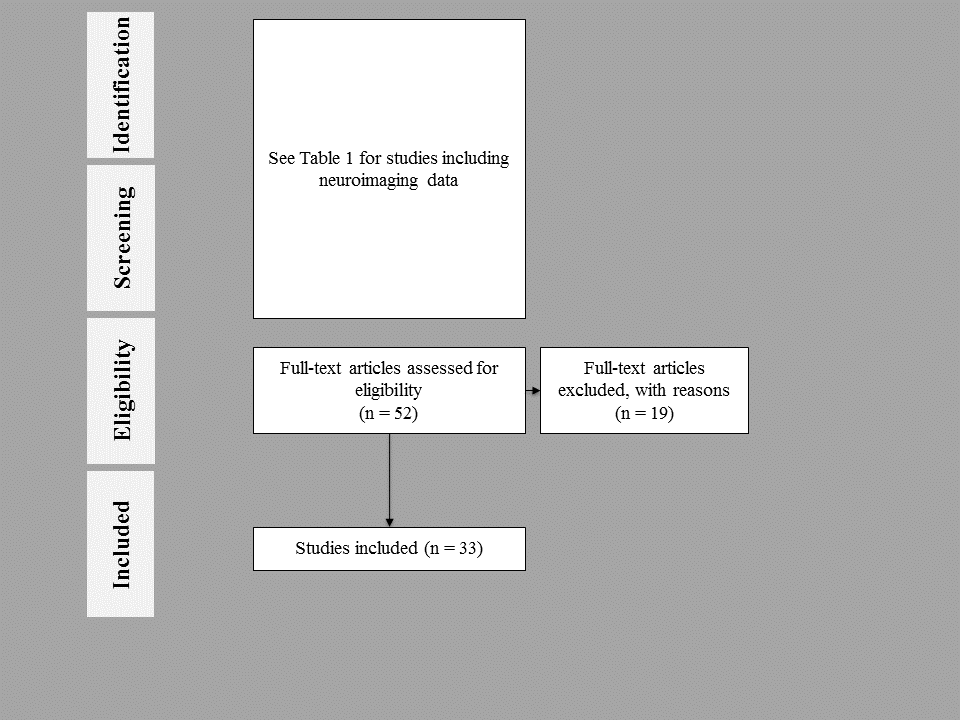


## SM8: Reasons for exclusion in the neuroimaging meta-analysis

Four studies were excluded because they did not report the contrasts of interest (Bertocci et al., 2012; Richter et al., 2013; Schweizer et al., 2013; Xin & Lei, 2015), twelve studies were excluded because they only reported the contrasts of interest within the context of interactions with mental health status (Burhan et al., 2016; Moon & Jeong, 2015; Morey et al., 2011; Mullin et al., 2012; Pallesen et al., 2010; Passarotti et al., 2012, 2011; Pavuluri et al., 2012; Sabharwal et al., 2016; Zhang et al., 2013), neurological disorders (Berger et al., 2015), or genotype (Luksys et al., 2014), Park et al. (2016) included only data from a patient group, Bertocci et al. (2014) showed no significant contrasts and data from one study were already included (Krause-Utz, Elzinga, Oei, Paret, et al., 2014).

### Table S5. Contrasts included in the neuroimaging meta-analysis

|  | Distractor | Memoranda |
| --- | --- | --- |
|  | Number of coordinates/contrasts/ studies | |
| **Affective > Neutral** |  |  |
| Positive > Neutral | 5/2/2 | 9/4/4 |
| Negative > Neutral | 151/16/15 | 62/8/8 |
| Positive & Negative > Neutral | 29/2/2 | 7/2/2 |
|  |  |  |
| **Neutral > Affective** |  |  |
| Neutral > Negative | 118/14/13 | 26/6/6 |
| Neutral > Positive | -/-/- | 30/4/4 |
| Neutral > Positive & Negative | -/-/- | 4/1/1 |
|  |  |  |
| **Others** |  |  |
| Negative & Neutral > Positive | -/-/- | 8/1/1 |
| Mixed > Neutral | -/-/- | 6/2/1 |
| Negative > Neutral & Positive | -/-/- | 1/1/1 |

### Table S6. Regions showing differential activation for affective vs. neutral stimuli during WM tasks

| Region | L/R | Cluster size (voxels) | Sub-cluster size (voxels) | Maximum | x/y/z |
| --- | --- | --- | --- | --- | --- |
| **Affective > Neutral Stimuli** |  |  |  |  |  |
| vlPFC/OFC | L | 371 |  | .28 | ‒42/30/‒6 |
|  |  |  | 348 |  | ‒42/30/‒6 |
|  |  |  | 23 |  | ‒30/30/6 |
| vlPFC | R | 452 |  | .22 | 51/36/9 |
|  |  |  | 200 |  | 51/36/9 |
|  |  |  | 148 |  | 39/33/‒9 |
|  |  |  | 68 |  | 42/15/30 |
|  |  |  | 36 |  | 48/24/21 |
| Amygdala | L | 267 |  | .27 | ‒24/‒3/‒18 |
|  |  |  | 231 |  | ‒24/‒3/‒18 |
|  |  |  | 36 |  | ‒21/‒6/‒6 |
| Temporal lobe (incl. amygdalo- | R | 593 |  | .40 | 21/‒6/‒18 |
| hippocampal complex) |  |  | 277 |  | 21/‒6/‒18 |
|  |  |  | 60 |  | 36/0/‒24 |
|  |  |  | 92 |  | 54/9/‒24 |
|  |  |  | 40 |  | 45/3/‒21 |
|  |  |  | 46 |  | 39/‒3/‒15 |
|  |  |  | 47 |  | 6/‒12/‒12 |
|  |  |  | 26 |  | 9/‒27/‒9 |
| Temporo-occipital lobe (including | L | 594 |  | .21 | ‒39/‒78/‒6 |
| fusiform gyrus) |  |  | 143 |  | ‒39/‒78/‒6 |
|  |  |  | 245 |  | ‒42/‒51/‒18 |
|  |  |  | 128 |  | ‒48/‒75/6 |
|  |  |  | 37 |  | ‒51/‒63/6 |
|  |  |  | 41 |  | ‒51/‒51/6 |
| Temporo-occipital lobe (including fusiform gyrus) | R | 591 |  | .28 | 42/‒54/‒18 |
|  |  |  | 188 |  | 42/‒54/‒18 |
|  |  |  | 200 |  | 39/‒75/‒12 |
|  |  |  | 161 |  | 54/‒69/6 |
|  |  |  | 42 |  | 42/‒54/‒6 |
| **Neutral > Affective Stimuli** |  |  |  |  |  |
| dlPFC | R | 480 |  | .46 | 36/33/33 |
|  |  |  | 229 |  | 36/33/33 |
|  |  |  | 166 |  | 36/42/30 |
|  |  |  | 34 |  | 27/51/12 |
|  |  |  | 51 |  | 33/54/18 |
| Precuneus | L | 403 |  | .21 | ‒3/‒69/60 |
|  |  |  | 61 |  | ‒3/‒69/60 |
|  |  |  | 60 |  | ‒6/‒72/51 |
|  | R |  | 47 |  | 6/‒66/60 |
|  |  |  | 89 |  | 0/‒72/24 |
|  |  |  | 43 |  | 12/‒66/45 |
|  |  |  | 36 |  | 3/‒69/36 |
|  |  |  | 42 |  | 12/‒63/33 |
|  |  |  | 25 |  | 18/‒63/66 |

*Table S6.* The affective > neutral comparison included 266 coordinates 36 contrasts. The neutral > affective included 178 coordinates from 25 contrasts. dl = dorsolateral, vl = ventrolateral, PFC = prefrontal cortex, L = left, R= right. Maximum = maximum of the *z* field.

### Table S7. Regions showing differential activation for affective vs. neutral task-irrelevant distractors

| Region | L/R | Cluster size (voxels) | Sub-cluster size (voxels) | Maximum | x/y/z |
| --- | --- | --- | --- | --- | --- |
| **Affective > neutral task-** |  |  |  |  |  |
| **irrelevant stimuli** |  |  |  |  |  |
| vlPFC | R | 398 |  | .33 | 51/36/9 |
|  |  |  | 179 |  | 51/36/9 |
|  |  |  | 103 |  | 42/15/30 |
|  |  |  | 39 |  | 48/24/21 |
|  |  |  | 20 |  | 39/30/18 |
|  |  |  | 57 |  | 48/9/39 |
| Temporal lobe (incl. amygdalo- | L | 310 |  | .41 | ‒24/0/‒18 |
| hippocampal complex) |  |  |  |  |  |
|  | R | 625 |  | .71 | 24/0/‒24 |
|  |  |  | 368 |  | 24/0/‒24 |
|  |  |  | 84 |  | 36/0/‒24 |
|  |  |  | 80 |  | 45/3/‒21 |
|  |  |  | 55 |  | 39/‒3/‒15 |
|  |  |  | 54 |  | 54/9/‒24 |
|  |  |  | 39 |  | 45/‒3/‒12 |
| Temporo-occipital lobe (including | L | 576 |  | .31 | ‒39/‒51/‒15 |
| fusiform gyrus) |  |  | 139 |  | ‒39/‒51/‒15 |
|  |  |  | 133 |  | ‒42/‒66/‒12 |
|  |  |  | 108 |  | ‒42/‒72/15 |
|  |  |  | 41 |  | ‒48/‒63/9 |
|  | R | 503 |  | .41 | 42/‒54/‒18 |
|  |  |  | 176 |  | 42/‒54/‒18 |
|  |  |  | 130 |  | 45/‒75/‒9 |
|  |  |  | 147 |  | 51/‒75/0 |
|  |  |  | 50 |  | 42/‒54/‒6 |
| **Neutral > affective task-** |  |  |  |  |  |
| **irrelevant stimuli** |  |  |  |  |  |
| dlPFC | L | 271 |  | .38 | ‒27/27/48 |
|  |  |  | 208 |  | ‒27/27/48 |
|  |  |  | 47 |  | ‒27/39/30 |
|  | R | 533 |  | .62 | 36/42/30 |
|  |  |  | 100 |  | 36/42/30 |
|  |  |  | 98 |  | 36/33/33 |
|  |  |  | 144 |  | 27/51/12 |
|  |  |  | 74 |  | 45/48/24 |
|  |  |  | 64 |  | 48/33/33 |
|  |  |  | 46 |  | 33/39/42 |

*Table S7.* The affective > neutral comparison included 183 coordinates from 20 contrasts. The neutral > affective included 63 coordinates from 14 contrasts. vl = ventrolateral, dl = dorsolateral; PFC = prefrontal cortex, L = left, R= right. Maximum = maximum of the *z* field.

## Supplementary References

Allen, R. J., Schaefer, A., & Falcon, T. (2014). Recollecting positive and negative autobiographical memories disrupts working memory. *Acta Psychologica*, *151*, 237–43. http://doi.org/10.1016/j.actpsy.2014.07.003

Alonso-Recio, L., Martin-Plasencia, P., Loeches-Alonso, A., & Serrano-Rodriguez, J. M. (2014). Working memory and facial expression recognition in patients with Parkinson’s disease. *Journal of the International Neuropsychological Society*, *20*(5), 496–505. http://doi.org/10.1017/s1355617714000265

Aronen, E. T., Vuontela, V., Steenari, M. R., Salmi, J., & Carlson, S. (2005). Working memory, psychiatric symptoms, and academic performance at school. *Neurobiology of Learning and Memory*, *83*(1), 33–42. http://doi.org/10.1016/j.nlm.2004.06.010

Augusti, E.-M., Torheim, H. K., & Melinder, A. (2014). The effect of emotional facial expressions on children’s working memory: Associations with age and behavior. *Child Neuropsychology*, *20*(1), 86–105. http://doi.org/10.1080/09297049.2012.749225

Avery, R. E., Smillie, L. D., & de Fockert, J. W. (2013). The role of working memory in achievement goal pursuit. *Acta Psychologica*, *144*(2), 361–72. http://doi.org/10.1016/j.actpsy.2013.07.012

Balodis, I. M., Johnsrude, I. S., & Olmstead, M. C. (2007). Intact preference conditioning in acute intoxication despite deficient declarative knowledge and working memory. *Alcoholism, Clinical and Experimental Research*, *31*(11), 1800–10. http://doi.org/10.1111/j.1530-0277.2007.00482.x

Banks, J. B., Tartar, J. L., & Tamayo, B. A. (2015). Examining factors involved in stress-related working memory impairments: Independent or conditional effects? *Emotion*, *15*(6), 827–36. http://doi.org/10.1037/emo0000096

Bauer, I. E., Jordan, G., Soares, J. C., & Meyer, T. D. (2015). The role of negative mood induction on working memory capacity in individuals putatively at risk for bipolar disorder: A pilot study. *Journal of Affective Disorders*, *185*, 60–6. http://doi.org/10.1016/j.jad.2015.05.068

Beneventi, H., Barndon, R., Ersland, L., & Hugdahl, K. (2007). An fMRI study of working memory for schematic facial expressions. *Scandinavian Journal of Psychology*, *48*(2), 81–86. http://doi.org/10.1111/j.1467-9450.2007.00536.x

Bennett, D. S., Mohamed, F. B., Carmody, D. P., Malik, M., Faro, S. H., & Lewis, M. (2013). Prenatal tobacco exposure predicts differential brain function during working memory in early adolescence: a preliminary investigation. *Brain Imaging and Behavior*, *7*(1), 49–59. http://doi.org/10.1007/s11682-012-9192-1

Bergmann, H. C., Rijpkema, M., Fernandez, G., & Kessels, R. P. (2012). The effects of valence and arousal on associative working memory and long-term memory. *PLoS One*, *7*(12), e52616. http://doi.org/10.1371/journal.pone.0052616

Berman, M., Nee, D., Casement, M., Kim, H., Deldin, P., Kross, E., … Jonides, J. (2011). Neural and behavioral effects of interference resolution in depression and rumination. *Cognitive, Affective, & Behavioral Neuroscience*, *11*(1), 85–96. http://doi.org/10.3758/s13415-010-0014-x

Brose, A., Lovden, M., & Schmiedek, F. (2014). Daily fluctuations in positive affect positively co-vary with working memory performance. *Emotion*, *14*(1), 1–6. http://doi.org/10.1037/a0035210

Brose, A., Schmiedek, F., Lovden, M., & Lindenberger, U. (2012). Daily variability in working memory is coupled with negative affect: the role of attention and motivation. *Emotion*, *12*(3), 605–17. http://doi.org/10.1037/a0024436

Buckert, M., Kudielka, B. M., Reuter, M., & Fiebach, C. J. (2012). The COMT Val158Met polymorphism modulates working memory performance under acute stress. *Psychoneuroendocrinology*, *37*(11), 1810–21. http://doi.org/10.1016/j.psyneuen.2012.03.014

Cao, H., Plichta, M. M., Schafer, A., Haddad, L., Grimm, O., Schneider, M., … Tost, H. (2014). Test-retest reliability of fMRI-based graph theoretical properties during working memory, emotion processing, and resting state. *NeuroImage*, *84*, 888–900. http://doi.org/10.1016/j.neuroimage.2013.09.013

Chen, Y., Norton, D., McBain, R., Ongur, D., & Heckers, S. (2009). Visual and cognitive processing of face information in schizophrenia: Detection, discrimination and working memory. *Schizophrenia Research*, *107*(1), 92–98. http://doi.org/10.1016/j.schres.2008.09.010

Cook, I. A., Bookheimer, S. Y., Mickes, L., Leuchter, A. F., & Kumar, A. (2007). Aging and brain activation with working memory tasks: an fMRI study of connectivity. *International Journal of Geriatric Psychiatry*, *22*(4), 332–42. http://doi.org/10.1002/gps.1678

Curci, A., Lanciano, T., Soleti, E., & Rime, B. (2013). Negative emotional experiences arouse rumination and affect working memory capacity. *Emotion*, *13*(5), 867–80. http://doi.org/10.1037/a0032492

Deckersbach, T., Rauch, S. L., Buhlmann, U., Ostacher, M. J., Beucke, J.-C., Nierenberg, A. A., … Dougherty, D. D. (2008). An fMRI investigation of working memory and sadness in females with bipolar disorder: a brief report. *Bipolar Disorders*, *10*(8), 928–942. http://doi.org/10.1111/j.1399-5618.2008.00633.x

DeYoung, C. G., Shamosh, N. A., Green, A. E., Braver, T. S., & Gray, J. R. (2009). Intellect as distinct from Openness: differences revealed by fMRI of working memory. *Journal of Personality and Social Psychology*, *97*(5), 883–92. http://doi.org/10.1037/a0016615

Dilworth-Bart, J., Poehlmann, J., Hilgendorf, A. E., Miller, K., & Lambert, H. (2010). Maternal scaffolding and preterm toddlers’ visual-spatial processing and emerging working memory. *Journal of Pediatric Psychology*, *35*(2), 209–20. http://doi.org/10.1093/jpepsy/jsp048

Dorahy, M. J., Irwin, H. J., & Middleton, W. (2004). Assessing markers of working memory function in dissociative identity disorder using neutral stimuli: a comparison with clinical and general population samples. *The Australian and New Zealand Journal of Psychiatry*, *38*(1–2), 47–55.

Dowson, J. H., Blackwell, A. D., Turner, D. C., Harvey, E., Malhotra, T., Robbins, T. W., & Sahakian, B. J. (2007). Questionnaire ratings of attention-deficit/hyperactivity disorder (ADHD) in adults are associated with spatial working memory. *European Psychiatry*, *22*(4), 256–63. http://doi.org/10.1016/j.eurpsy.2006.08.005

Drapier, D., Surguladze, S., Marshall, N., Schulze, K., Fern, A., Hall, M. H., … McDonald, C. (2008). Genetic liability for bipolar disorder is characterized by excess frontal activation in response to a working memory task. *Biological Psychiatry*, *64*(6), 513–20. http://doi.org/10.1016/j.biopsych.2008.04.038

Dretsch, M. N., & Tipples, J. (2008). Working memory involved in predicting future outcomes based on past experiences. *Brain and Cognition*, *66*(1), 83–90. http://doi.org/10.1016/j.bandc.2007.05.006

El-Hage, W., Gaillard, P., Isingrini, M., & Belzung, C. (2006). Trauma-related deficits in working memory. *Cognitive Neuropsychiatry*, *11*(1), 33–46. http://doi.org/10.1080/13546800444000164

Engelhard, I. M., van den Hout, M. A., Dek, E. C. P., Giele, C. L., van der Wielen, J.-W., Reijnen, M. J., & van Roij, B. (2011). Reducing vividness and emotional intensity of recurrent ‘flashforwards’ by taxing working memory: An analogue study. *Journal of Anxiety Disorders*, *25*(4), 599–603. http://doi.org/10.1016/j.janxdis.2011.01.009

Engelhard, I. M., van den Hout, M. A., & Smeets, M. A. M. (2011). Taxing working memory reduces vividness and emotional intensity of images about the Queen’s Day tragedy. *Journal of Behavior Therapy and Experimental Psychiatry*, *42*(1), 32–37.

Epperson, C. N., Amin, Z., Ruparel, K., Gur, R., & Loughead, J. (2012). Interactive effects of estrogen and serotonin on brain activation during working memory and affective processing in menopausal women. *Psychoneuroendocrinology*, *37*(3), 372–82. http://doi.org/10.1016/j.psyneuen.2011.07.007

Faridi, N., Karama, S., Burgaleta, M., White, M. T., Evans, A. C., Fonov, V., … Waber, D. P. (2015). Neuroanatomical correlates of behavioral rating versus performance measures of working memory in typically developing children and adolescents. *Neuropsychology*, *29*(1), 82–91. http://doi.org/10.1037/neu0000079

Freeman, D., Startup, H., Dunn, G., Cernis, E., Wingham, G., Pugh, K., … Kingdon, D. (2013). The interaction of affective with psychotic processes: a test of the effects of worrying on working memory, jumping to conclusions, and anomalies of experience in patients with persecutory delusions. *J Psychiatr Res*, *47*(12), 1837–42. http://doi.org/10.1016/j.jpsychires.2013.06.016

Fuge, P., Aust, S., Fan, Y., Weigand, A., Gartner, M., Feeser, M., … Grimm, S. (2014). Interaction of early life stress and corticotropin-releasing hormone receptor gene: effects on working memory. *Biol Psychiatry*, *76*(11), 888–94. http://doi.org/10.1016/j.biopsych.2014.04.016

Gartner, M., Rohde-Liebenau, L., Grimm, S., & Bajbouj, M. (2014). Working memory-related frontal theta activity is decreased under acute stress. *Psychoneuroendocrinology*, *43*, 105–13. http://doi.org/10.1016/j.psyneuen.2014.02.009

Gohier, B., Ferracci, L., Surguladze, S. A., Lawrence, E., El Hage, W., Kefi, M. Z., … Le Gall, D. (2009). Cognitive inhibition and working memory in unipolar depression. *Journal of Affective Disorders*, *116*(1–2), 100–105. http://doi.org/10.1016/j.jad.2008.10.028

Gokcen, S., Bora, E., Erermis, S., Kesikci, H., & Aydin, C. (2009). Theory of mind and verbal working memory deficits in parents of autistic children. *Psychiatry Research*, *166*(1), 46–53. http://doi.org/10.1016/j.psychres.2007.11.016

Gonzalez-Garrido, A. A., Ramos-Loyo, J., Gomez-Velazquez, F. R., Alarcón, M. A., & Moises de la Serna Tuya, J. (2007). Visual verbal working memory processing may be interfered by previously seen faces. *International Journal of Psychophysiology*, *65*(2), 141–151. http://doi.org/10.1016/j.ijpsycho.2007.04.005

Gotoh, F. (2008). Influence of affective valence on working memory processes. *\international Journal of Psychology*, *43*(1), 59–71. http://doi.org/10.1080/00207590701318306

Grimm, S., Gartner, M., Fuge, P., Fan, Y., Weigand, A., Feeser, M., … Bajbouj, M. (2015). Variation in the corticotropin-releasing hormone receptor 1 (CRHR1) gene modulates age effects on working memory. *Journal of Psychiatric Research*, *61*, 57–63. http://doi.org/10.1016/j.jpsychires.2014.12.001

Grunwald, M., Weiss, T., Mueller, S., & Rall, L. (2014). EEG changes caused by spontaneous facial self-touch may represent emotion regulating processes and working memory maintenance. *Brain Research*, *1557*, 111–26. http://doi.org/10.1016/j.brainres.2014.02.002

Habel, U., Koch, K., Pauly, K., Kellermann, T., Reske, M., Backes, V., … Schneider, F. (2007). The influence of olfactory-induced negative emotion on verbal working memory: Individual differences in neurobehavioral findings. *Brain Research*, *1152*(4), 158–170. http://doi.org/10.1016/j.brainres.2007.03.048

Haldane, M., Jogia, J., Cobb, A., Kozuch, E., Kumari, V., & Frangou, S. (2008). Changes in brain activation during working memory and facial recognition tasks in patients with bipolar disorder with Lamotrigine monotherapy. *European Neuropsychopharmacology*, *18*(1), 48–54. http://doi.org/10.1016/j.euroneuro.2007.05.009

Hillary, F. G., Chiaravalloti, N. D., Ricker, J. H., Steffener, J., Bly, B. M., Lange, G., … DeLuca, J. (2003). An investigation of working memory rehearsal in multiple sclerosis using fMRI. *Journal of Clinical and Experimental Neuropsychology*, *25*(7), 965–78. http://doi.org/10.1076/jcen.25.7.965.16490

Hood, A., Pulvers, K., Spady, T. J., Kliebenstein, A., & Bachand, J. (2015). Anxiety mediates the effect of acute stress on working memory performance when cortisol levels are high: a moderated mediation analysis. *Anxiety, Stress, and Coping*, *28*(5), 545–62. http://doi.org/10.1080/10615806.2014.1000880

Huang, J., Tan, S. P., Walsh, S. C., Spriggens, L. K., Neumann, D. L., Shum, D. H., & Chan, R. C. (2014). Working memory dysfunctions predict social problem solving skills in schizophrenia. *Psychiatry Research*, *220*(1–2), 96–101. http://doi.org/10.1016/j.psychres.2014.07.043

Jackson, M. C., Linden, D. E., & Raymond, J. E. (2014). Angry expressions strengthen the encoding and maintenance of face identity representations in visual working memory. *Cogn Emot*, *28*(2), 278–97. http://doi.org/10.1080/02699931.2013.816655

Jeffries, S., & Everatt, J. (2004). Working memory: its role in dyslexia and other specific learning difficulties. *Dyslexia*, *10*(3), 196–214. http://doi.org/10.1002/dys.278

Jha, A. P., Stanley, E. A., Kiyonaga, A., Wong, L., & Gelfand, L. (2010). Examining the protective effects of mindfulness training on working memory capacity and affective experience. *Emotion*, *10*(1), 54–64. http://doi.org/10.1037/a0018438

Joormann, J., & Gotlib, I. H. (2008). Updating the contents of working memory in depression: Interference from irrelevant negative material. *Journal of Abnormal Psychology*, *117*(1), 182–192. http://doi.org/10.1037/0021-843X.117.1.182

Judah, M. R., Grant, D. M., Lechner, W. V., & Mills, A. C. (2013). Working memory load moderates late attentional bias in social anxiety. *Cognition & Emotion*, *27*(3), 502–11. http://doi.org/10.1080/02699931.2012.719490

Kane, M. J., Brown, L. H., McVay, J. C., Silvia, P. J., Myin-Germeys, I., & Kwapil, T. R. (2007). For whom the mind wanders, and when: An experience-sampling study of working memory and cognitive control in daily life. *Psychological Science*, *18*(7), 614–621.

Kerns, J. G., & Becker, T. M. (2008). Communication disturbances, working memory, and emotion in people with elevated disorganized schizotypy. *Schizophrenia Research*, *100*(1–3), 172–80. http://doi.org/10.1016/j.schres.2007.11.005

Klemen, J., Buchel, C., Buhler, M., Menz, M. M., & Rose, M. (2010). Auditory working memory load impairs visual ventral stream processing: toward a unified model of attentional load. *Journal of Cognitive Neuroscience*, *22*(3), 437–46. http://doi.org/10.1162/jocn.2009.21204

Konen, T., Dirk, J., & Schmiedek, F. (2015). Cognitive benefits of last night’s sleep: daily variations in children’s sleep behavior are related to working memory fluctuations. *Journal of Child Psychology and Psychiatry, and Allied Disciplines*, *56*(2), 171–82. http://doi.org/10.1111/jcpp.12296

Kostandov, E. A., Kurova, N. S., Cheremushkin, E. A., Petrenko, N. E., Ashkinazi, M. L., & Yakovenko, I. A. (2009). Relationship between the plasticity of a set to an emotional facial expression and the load on working memory. *Neuroscience and Behavioral Physiology*, *39*(3), 223–9. http://doi.org/10.1007/s11055-009-9126-6

Levens, S. M., Devinsky, O., & Phelps, E. A. (2011). Role of the left amygdala and right orbital frontal cortex in emotional interference resolution facilitation in working memory. *Neuropsychologia*, *49*(12), 3201–12. http://doi.org/10.1016/j.neuropsychologia.2011.07.021

Levens, S. M., & Gotlib, I. H. (2009). Impaired selection of relevant positive information in depression. *Depression and Anxiety*, *26*(5), 403–410.

Levens, S. M., & Phelps, E. A. (2008). Emotion processing effects on interference resolution in working memory. *Emotion*, *8*(2), 267–280.

Levens, S. M., & Phelps, E. A. (2010). Insula and Orbital Frontal Cortex Activity Underlying Emotion Interference Resolution in Working Memory. *Journal of Cognitive Neuroscience*, *22*(12), 2790–2803. http://doi.org/10.1162/jocn.2010.21428

Li, X., Chan, R. C., & Luo, Y. J. (2010). Stage effects of negative emotion on spatial and verbal working memory. *BMC Neuroscience*, *11*, 60. http://doi.org/10.1186/1471-2202-11-60

Lilley, S. A., Andrade, J., Turpin, G., Sabin-Farrell, R., & Holmes, E. A. (2009). Visuospatial working memory interference with recollections of trauma. *British Journal of Clinical Psychology*, *48*(Pt 3), 309–21. http://doi.org/10.1348/014466508x398943

Lim, S. L., Bruce, A. S., & Aupperle, R. L. (2014). The influence of a working memory task on affective perception of facial expressions. *PLoS One*, *9*(10), e111074. http://doi.org/10.1371/journal.pone.0111074

Linden, D. E., Lancaster, T. M., Wolf, C., Baird, A., Jackson, M. C., Johnston, S. J., … Thome, J. (2013). ZNF804A genotype modulates neural activity during working memory for faces. *Neuropsychobiology*, *67*(2), 84–92. http://doi.org/10.1159/000344001

Linden, S. C., Jackson, M. C., Subramanian, L., Healy, D., & Linden, D. E. J. (2011). Sad benefit in face working memory: An emotional bias of melancholic depression. *Journal of Affective Disorders*, *135*, 251–257.

Lindström, B. R., & Bohlin, G. (2012). Threat-relevance impairs executive functions: Negative impact on working memory and response inhibition. *Emotion*, *12*(2), 384–393. http://doi.org/10.1037/a0027305

LoPresti, M. L., Schon, K., Tricarico, M. D., Swisher, J. D., Celone, K. A., & Stern, C. E. (2008). Working memory for social cues recruits orbitofrontal cortex and amygdala: A functional magnetic resonance imaging study of delayed matching to sample for emotional expressions. *Journal of Neuroscience*, *28*(14), 3718–3728. http://doi.org/10.1523/JNEUROSCI.0464-08.2008

Lowe, J., MacLean, P. C., Shaffer, M. L., & Watterberg, K. (2009). Early working memory in children born with extremely low birth weight: assessed by object permanence. *Journal of Child Neurology*, *24*(4), 410–5. http://doi.org/10.1177/0883073808324533

Mammarella, N., Fairfield, B., Frisullo, E., & Di Domenico, A. (2013). Saying it with a natural child’s voice! When affective auditory manipulations increase working memory in aging. *Aging & Mental Health*, *17*(7), 853–62. http://doi.org/10.1080/13607863.2013.790929

Mano, Q. R., Brown, G. G., Mirzakhanian, H., Bolden, K., Cadenhead, K. S., & Light, G. A. (2014). Not all distraction is bad: working memory vulnerability to implicit socioemotional distraction correlates with negative symptoms and functional impairment in psychosis. *Schizophrenia Research and Treatment*, *2014*, 320948. http://doi.org/10.1155/2014/320948

Martin, G. N., & Chaudry, A. (2014). Working memory performance and exposure to pleasant and unpleasant ambient odor: is spatial span special? *International Journal of Neuroscience*, *124*(11), 806–11. http://doi.org/10.3109/00207454.2014.890619

Mattarella-Micke, A., Mateo, J., Kozak, M. N., Foster, K., & Beilock, S. L. (2011). Choke or thrive? The relation between salivary cortisol and math performance depends on individual differences in working memory and math-anxiety. *Emotion*, *11*(4), 1000–5. http://doi.org/10.1037/a0023224

Mehta, M. A., Hinton, E. C., Montgomery, A. J., Bantick, R. A., & Grasby, P. M. (2005). Sulpiride and mnemonic function: effects of a dopamine D2 receptor antagonist on working memory, emotional memory and long-term memory in healthy volunteers. *Journal of Psychopharmacology*, *19*(1), 29–38. http://doi.org/10.1177/0269881105048889

Mitchell, R. L. C. (2007). fMRI delineation of working memory for emotional prosody in the brain: commonalities with the lexico-semantic emotion network. *NeuroImage*, *36*(3), 1015–1025. http://doi.org/10.1016/j.neuroimage.2007.03.016

Monfort, V., Bernardin, F., Grosdemange, A., Ducrocq, X., Mathieu, P., & Bolmont, B. (2013). Paradoxical state anxiety and working memory in a patient with acute stroke. *Cognitive and Behavioral Neurology*, *26*(4), 195–207. http://doi.org/10.1097/wnn.0000000000000010

Moore, A. B., Clark, B. A., & Kane, M. J. (2008). Who shalt not kill? Individual differences in working memory capacity, executive control, and moral judgment. *Psychological Science*, *19*(6), 549–57. http://doi.org/10.1111/j.1467-9280.2008.02122.x

Moreno, M. L., Vanderhasselt, M. A., Carvalho, A. F., Moffa, A. H., Lotufo, P. A., Bensenor, I. M., & Brunoni, A. R. (2015). Effects of acute transcranial direct current stimulation in hot and cold working memory tasks in healthy and depressed subjects. *Neuroscience Letters*, *591*, 126–31. http://doi.org/10.1016/j.neulet.2015.02.036

Morgan, B., Terburg, D., Thornton, H. B., Stein, D. J., & van Honk, J. (2012). Paradoxical facilitation of working memory after basolateral amygdala damage. *PLoS One*, *7*(6), e38116. http://doi.org/10.1371/journal.pone.0038116

Moriya, J., Koster, E. H., & De Raedt, R. (2014). The influence of working memory on visual search for emotional facial expressions. *Journal of Experimental Psychology: Human Perception and Performance*, *40*(5), 1874–90. http://doi.org/10.1037/a0037295

Morra, S., Parrella, I., & Camba, R. (2011). The role of working memory in the development of emotion comprehension. *British Journal of Developmental Psychology*, *29*(4), 744–64. http://doi.org/10.1348/2044-835x.002006

Mu, Y. G., Huang, L. J., Li, S. Y., Ke, C., Chen, Y., Jin, Y., & Chen, Z. P. (2012). Working memory and the identification of facial expression in patients with left frontal glioma. *Neuro-Oncology*, *14 Suppl 4*, iv81-9. http://doi.org/10.1093/neuonc/nos215

Mueller, S. C., Shechner, T., Rosen, D., Nelson, E. E., Pine, D. S., & Ernst, M. (2015). Incidental threat during visuospatial working memory in adolescent anxiety: an emotional memory-guided saccade task. *Depression and Anxiety*, *32*(4), 289–95. http://doi.org/10.1002/da.22350

Mulder, H., Pitchford, N. J., & Marlow, N. (2011). Inattentive behaviour is associated with poor working memory and slow processing speed in very pre-term children in middle childhood. *British Journal of Educational Psychology*, *81*(Pt 1), 147–60. http://doi.org/10.1348/000709910x505527

Muller, U., Mottweiler, E., & Bublak, P. (2005). Noradrenergic blockade and numeric working memory in humans. *Journal of Psychopharmacology*, *19*(1), 21–8. http://doi.org/10.1177/0269881105048888

Oei, N. Y., Everaerd, W. T., Elzinga, B. M., van Well, S., & Bermond, B. (2006). Psychosocial stress impairs working memory at high loads: an association with cortisol levels and memory retrieval. *Stress*, *9*(3), 133–41. http://doi.org/10.1080/10253890600965773

Opmeer, E. M., Kortekaas, R., van Tol, M. J., van der Wee, N. J., Woudstra, S., van Buchem, M. A., … Aleman, A. (2013). Influence of COMT val158met genotype on the depressed brain during emotional processing and working memory. *PLoS One*, *8*(9), e73290. http://doi.org/10.1371/journal.pone.0073290

Park, S., Gibson, C., & McMichael, T. (2006). Socioaffective factors modulate working memory in schizophrenia patients. *Neuroscience*, *139*(1), 373–84. http://doi.org/10.1016/j.neuroscience.2005.06.034

Pauly, K., Seiferth, N. Y., Kellermann, T., Ruhrmann, S., Daumann, B., Backes, V., … Habel, U. (2010). The interaction of working memory and emotion in persons clinically at risk for psychosis: an fMRI pilot study. *Schizophrenia Research*, *120*(1–3), 167–76. http://doi.org/10.1016/j.schres.2009.12.008

Pe, M. L., Koval, P., & Kuppens, P. (2013). Executive well-being: Updating of positive stimuli in working memory is associated with subjective well-being. *Cognition*, *126*(2), 335–340.

Pe, M. L., Raes, F., & Kuppens, P. (2013). The cognitive building blocks of emotion regulation: ability to update working memory moderates the efficacy of rumination and reappraisal on emotion. *PloS One*, *8*(7), e69071.

Pecchinenda, A., Dretsch, M., & Chapman, P. (2006). Working memory involvement in emotion-based processes underlying choosing advantageously. *Experimental Psychology*, *53*(3), 191–7. http://doi.org/10.1027/1618-3169.53.3.191

Perlstein, W. M., Elbert, T., & Stenger, V. A. (2002). Dissociation in human prefrontal cortex of affective influences on working memory-related activity. *Proceedings of the National Academy of Sciences*, *99*(3), 1736–1741. http://doi.org/10.1073/pnas.241650598

Phillips, L. H., Channon, S., Tunstall, M., Hedenstrom, A., & Lyons, K. (2008). The role of working memory in decoding emotions. *Emotion*, *8*(2), 184–91. http://doi.org/10.1037/1528-3542.8.2.184

Putman, P., Hermans, E. J., & van Honk, J. (2007). Exogenous cortisol shifts a motivated bias from fear to anger in spatial working memory for facial expressions. *Psychoneuroendocrinology*, *32*(1), 14–21. http://doi.org/10.1016/j.psyneuen.2006.09.010

Qi, S., Ding, C., & Li, H. (2014). Neural correlates of inefficient filtering of emotionally neutral distractors from working memory in trait anxiety. *Cognitive, Affective & Behavioral Neuroscience*, *14*(1), 253–65. http://doi.org/10.3758/s13415-013-0203-5

Reinecke, A., Rinck, M., & Becker, E. S. (2006). Spiders crawl easily through the bottleneck: Visual working memory for negative stimuli. *Emotion*, *6*(3), 438–449. http://doi.org/10.1037/1528-3542.6.3.438

Robinson, K. E., Pearson, M. M., Cannistraci, C. J., Anderson, A. W., Kuttesch, J. F., Jr., Wymer, K., … Compas, B. E. (2015). Functional neuroimaging of working memory in survivors of childhood brain tumors and healthy children: Associations with coping and psychosocial outcomes. *Child Neuropsychology*, *21*(6), 779–802. http://doi.org/10.1080/09297049.2014.924492

Rosenberg, H., Dethier, M., Kessels, R. P., Westbrook, R. F., & McDonald, S. (2015). Emotion perception after moderate-severe traumatic brain injury: The valence effect and the role of working memory, processing speed, and nonverbal reasoning. *Neuropsychology*, *29*(4), 509–21. http://doi.org/10.1037/neu0000171

Salvadore, G., Cornwell, B. R., Sambataro, F., Latov, D., Colon-Rosario, V., Carver, F., … Zarate, C. A., Jr. (2010). Anterior cingulate desynchronization and functional connectivity with the amygdala during a working memory task predict rapid antidepressant response to ketamine. *Neuropsychopharmacology*, *35*(7), 1415–22. http://doi.org/10.1038/npp.2010.24

Saunders, N., Downham, R., Turman, B., Kropotov, J., Clark, R., Yumash, R., & Szatmary, A. (2015). Working memory training with tDCS improves behavioral and neurophysiological symptoms in pilot group with post-traumatic stress disorder (PTSD) and with poor working memory. *Neurocase*, *21*(3), 271–8. http://doi.org/10.1080/13554794.2014.890727

Schaefer, A., Braver, T. S., Reynolds, J. R., Burgess, G. C., Yarkoni, T., & Gray, J. R. (2006). Individual differences in amygdala activity predict response speed during working memory. *Journal of Neuroscience*, *26*(40), 10120–8. http://doi.org/10.1523/jneurosci.2567-06.2006

Schenkel, L. S., Passarotti, A. M., Sweeney, J. A., & Pavuluri, M. N. (2012). Negative emotion impairs working memory in pediatric patients with bipolar disorder type I. *Psychological Medicine*, *42*(12), 2567–2577. http://doi.org/10.1017/S0033291712000797

Schmeichel, B. J., Volokhov, R. N., & Demaree, H. A. (2008). Working memory capacity and the self-regulation of emotional expression and experience. *Journal of Personality and Social Psychology*, *95*(6), 1526–40. http://doi.org/10.1037/a0013345

Schneider, F., Koch, K., Reske, M., Kellermann, T., Seiferth, N., Stocker, T., … Habel, U. (2006). Interaction of negative olfactory stimulation and working memory in schizophrenia patients: development and evaluation of a behavioral neuroimaging task. *Psychiatry Research*, *144*(2–3), 123–30. http://doi.org/10.1016/j.psychres.2004.12.013

Sessa, P., Luria, R., Gotler, A., Jolicoeur, P., & Dell’acqua, R. (2011). Interhemispheric ERP asymmetries over inferior parietal cortex reveal differential visual working memory maintenance for fearful versus neutral facial identities. *Psychophysiology*, *48*(2), 187–97. http://doi.org/10.1111/j.1469-8986.2010.01046.x

Shackman, A. J., Sarinopoulos, I., Maxwell, J. S., Pizzagalli, D. A., Lavric, A., & Davidson, R. J. (2006). Anxiety selectively disrupts visuospatial working memory. *Emotion*, *6*(1), 40–61. http://doi.org/10.1037/1528-3542.6.1.40

Sharbanee, J. M., Stritzke, W. G., Wiers, R. W., Young, P., Rinck, M., & MacLeod, C. (2013). The interaction of approach-alcohol action tendencies, working memory capacity, and current task goals predicts the inability to regulate drinking behavior. *Psychology of Adictive Behaviors*, *27*(3), 649–61. http://doi.org/10.1037/a0029982

Shi, Z., Gao, X., & Zhou, R. (2015). Frontal theta activity during working memory in test anxiety. *Neuroreport*, *26*(4), 228–32. http://doi.org/10.1097/wnr.0000000000000334

Silver, H., & Feldman, P. (2005). Evidence for sustained attention and working memory in schizophrenia sharing a common mechanism. *Journal of Neuropsychiatry and Clinical Neurosciences*, *17*(3), 391–8. http://doi.org/10.1176/jnp.17.3.391

Silver, H., Feldman, P., Bilker, W., & Gur, R. C. (2003). Working memory deficit as a core neuropsychological dysfunction in schizophrenia. *American Journal of Psychiatry*, *160*(10), 1809–16. http://doi.org/10.1176/appi.ajp.160.10.1809

Skodol, A. E., Oldham, J. M., Bender, D. S., Dyck, I. R., Stout, R. L., Morey, L. C., … others. (2005). Dimensional representations of DSM-IV personality disorders: relationships to functional impairment. *American Journal of Psychiatry*, *162*(10), 1919.

Smith, M. J., Horan, W. P., Cobia, D. J., Karpouzian, T. M., Fox, J. M., Reilly, J. L., & Breiter, H. C. (2014). Performance-based empathy mediates the influence of working memory on social competence in schizophrenia. *Schizophrenia Bulletin*, *40*(4), 824–34. http://doi.org/10.1093/schbul/sbt084

Spachtholz, P., Kuhbandner, C., & Pekrun, R. (2014). Negative affect improves the quality of memories: trading capacity for precision in sensory and working memory. *Journal of Experimental Psychology: General*, *143*(4), 1450–6. http://doi.org/10.1037/xge0000012

Spitzer, B., Gloel, M., Schmidt, T. T., & Blankenburg, F. (2014). Working memory coding of analog stimulus properties in the human prefrontal cortex. *Cerebral Cortex*, *24*(8), 2229–36. http://doi.org/10.1093/cercor/bht084

Stegmayer, K., Usher, J., Trost, S., Henseler, I., Tost, H., Rietschel, M., … Gruber, O. (2015). Disturbed cortico-amygdalar functional connectivity as pathophysiological correlate of working memory deficits in bipolar affective disorder. *European Archives of Psychiatry and Clinical Neuroscience*, *265*(4), 303–11. http://doi.org/10.1007/s00406-014-0517-5

Storbeck, J., Davidson, N. A., Dahl, C. F., Blass, S., & Yung, E. (2015). Emotion, working memory task demands and individual differences predict behavior, cognitive effort and negative affect. *Cognition & Emotion*, *29*(1), 95–117. http://doi.org/10.1080/02699931.2014.904222

Storbeck, J., & Watson, P. (2014). Verbal makes it positive, spatial makes it negative: working memory biases judgments, attention, and moods. *Emotion*, *14*(6), 1072–86. http://doi.org/10.1037/a0037327

Stout, D. M., Shackman, A. J., Johnson, J. S., & Larson, C. L. (2015). Worry is associated with impaired gating of threat from working memory. *Emotion*, *15*(1), 6–11. http://doi.org/10.1037/emo0000015

Straube, T., Trippe, R., Schmidt, S., Weiss, T., Hecht, H., & Miltner, W. H. (2011). Dissociation of acquisition and expression of fear conditioned responses under working memory load. *Emotion*, *11*(1), 209–13. http://doi.org/10.1037/a0021157

Strauss, G. P., Lee, B. G., Waltz, J. A., Robinson, B. M., Brown, J. K., & Gold, J. M. (2012). Cognition-emotion interactions are modulated by working memory capacity in individuals with schizophrenia. *Schizophrenia Research*, *141*(2–3), 257–61. http://doi.org/10.1016/j.schres.2012.08.010

Takahashi, H., Yamada, M., & Suhara, T. (2012). Functional significance of central D1 receptors in cognition: beyond working memory. *Journal of Cerebral Blood Flow and Metabolism*, *32*(7), 1248–58. http://doi.org/10.1038/jcbfm.2011.194

Thermenos, H. W., Goldstein, J. M., Milanovic, S. M., Whitfield-Gabrieli, S., Makris, N., Laviolette, P., … Seidman, L. J. (2010). An fMRI study of working memory in persons with bipolar disorder or at genetic risk for bipolar disorder. *American Journal of Medical Genetics. Part B, Neuropsychiatric Genetics: The Official Publication of the International Society of Psychiatric Genetics*, *153b*(1), 120–31. http://doi.org/10.1002/ajmg.b.30964

Thermenos, H. W., Makris, N., Whitfield-Gabrieli, S., Brown, A. B., Giuliano, A. J., Lee, E. H., … Seidman, L. J. (2011). A functional MRI study of working memory in adolescents and young adults at genetic risk for bipolar disorder: preliminary findings. *Bipolar Disorders*, *13*(3), 272–86. http://doi.org/10.1111/j.1399-5618.2011.00920.x

Thiruchselvam, R., Hajcak, G., & Gross, J. J. (2012). Looking inward: shifting attention within working memory representations alters emotional responses. *Psychological Science*, *23*(12), 1461–6. http://doi.org/10.1177/0956797612449838

Thomas, P. M., Jackson, M. C., & Raymond, J. E. (2014). A threatening face in the crowd: effects of emotional singletons on visual working memory. *Journal of Experimental Psychology: Human Perception and Performance*, *40*(1), 253–63. http://doi.org/10.1037/a0033970

Tomasi, D., Ernst, T., Caparelli, E. C., & Chang, L. (2006). Common deactivation patterns during working memory and visual attention tasks: an intra-subject fMRI study at 4 Tesla. *Human Brain Mapping*, *27*(8), 694–705. http://doi.org/10.1002/hbm.20211

Trezise, K., & Reeve, R. A. (2014). Working memory, worry, and algebraic ability. *J Exp Child Psychol*, *121*, 120–36. http://doi.org/10.1016/j.jecp.2013.12.001

Tsai, C., & McNally, R. J. (2014). Effects of emotionally valenced working memory taxation on negative memories. *Journal of Behavior Therapy and Experimental Psychiatry*, *45*(1), 15–9. http://doi.org/10.1016/j.jbtep.2013.07.004

van den Hout, M. A., Eidhof, M. B., Verboom, J., Littel, M., & Engelhard, I. M. (2014). Blurring of emotional and non-emotional memories by taxing working memory during recall. *Cognition & Emotion*, *28*(4), 717–27. http://doi.org/10.1080/02699931.2013.848785

van den Hout, M. A., Engelhard, I. M., Beetsma, D., Slofstra, C., Hornsveld, H., Houtveen, J., & Leer, A. (2011). EMDR and mindfulness. Eye movements and attentional breathing tax working memory and reduce vividness and emotionality of aversive ideation. *Journal of Behavior Therapy and Experimental Psychiatry*, *42*(4), 423–431. http://doi.org/10.1016/j.jbtep.2011.03.004

Van Dillen, L. F., & Derks, B. (2012). Working memory load reduces facilitated processing of threatening faces: an ERP study. *Emotion*, *12*(6), 1340–9. http://doi.org/10.1037/a0028624

Van Dillen, L. F., & Koole, S. L. (2007). Clearing the mind: a working memory model of distraction from negative mood. *Emotion*, *7*(4), 715–23. http://doi.org/10.1037/1528-3542.7.4.715

Vugs, B., Hendriks, M., Cuperus, J., & Verhoeven, L. (2014). Working memory performance and executive function behaviors in young children with SLI. *Research in Developmental Disabilities*, *35*(1), 62–74. http://doi.org/10.1016/j.ridd.2013.10.022

Vuontela, V., Carlson, S., Troberg, A. M., Fontell, T., Simola, P., Saarinen, S., & Aronen, E. T. (2013). Working memory, attention, inhibition, and their relation to adaptive functioning and behavioral/emotional symptoms in school-aged children. *Child Psychiatry and Human Development*, *44*(1), 105–22. http://doi.org/10.1007/s10578-012-0313-2

Wang, M., & Saudino, K. J. (2013). Genetic and environmental influences on individual differences in emotion regulation and its relation to working memory in toddlerhood. *Emotion*, *13*(6), 1055–67. http://doi.org/10.1037/a0033784

Wolf, C., Jackson, M. C., Kissling, C., Thome, J., & Linden, D. E. J. (2011). Dysbindin-1 genotype effects on emotional working memory. *Molecular Psychiatry*, *16*(2), 145–155.

Wolf, C., Linden, S., Jackson, M. C., Healy, D., Baird, A., Linden, D. E., & Thome, J. (2011). Brain activity supporting working memory accuracy in patients with paranoid schizophrenia: a functional magnetic resonance imaging study. *Neuropsychobiology*, *64*(2), 93–101. http://doi.org/10.1159/000323800

Wolfe, C. D., & Bell, M. A. (2007). The integration of cognition and emotion during infancy and early childhood: Regulatory processes associated with the development of working memory. *Brain and Cognition*, *65*(1), 3–13.

Yogo, M., & Fujihara, S. (2008). Working memory capacity can be improved by expressive writing: a randomized experiment in a Japanese sample. *British Journal of Health Psychology*, *13*(1), 77–80. http://doi.org/10.1348/135910707x252440

Yoon, K. L., LeMoult, J., & Joormann, J. (2014). Updating emotional content in working memory: a depression-specific deficit? *Journal of Behavior Therapy and Experimental Psychiatry*, *45*(3), 368–74. http://doi.org/10.1016/j.jbtep.2014.03.004

Ziaei, M., Peira, N., & Persson, J. (2014). Brain systems underlying attentional control and emotional distraction during working memory encoding. *NeuroImage*, *87*, 276–86. http://doi.org/10.1016/j.neuroimage.2013.10.048
